# Identification of expressed genes during compatible interaction between stripe rust (*Puccinia striiformis*) and wheat using a cDNA library

**DOI:** 10.1186/1471-2164-10-586

**Published:** 2009-12-08

**Authors:** Jinbiao Ma, Xueling Huang, Xiaojie Wang, Xianming Chen, Zhipeng Qu, Lili Huang, Zhensheng Kang

**Affiliations:** 1 College of Life Sciences, Northwest A&F University, Yangling, Shaanxi, 712100, PR China; 2 College of Plant Protection and Shaanxi Key Laboratory of Molecular Biology for Agriculture, Northwest A&F University, Yangling, Shaanxi, 712100, PR China; 3 USDA-ARS and Department of Plant Pathology, Washington State University, Pullman, WA 99164-6430, USA

## Abstract

**Background:**

Wheat stripe rust, caused by *Puccinia striiformis *f. sp. *tritici *(*Pst*), is one of the most destructive diseases of wheat worldwide. To establish compatibility with the host, *Pst *forms special infection structures to invade the plant with minimal damage to host cells. Although compatible interaction between wheat and *Pst *has been studied using various approaches, research on molecular mechanisms of the interaction is limited. The aim of this study was to develop an EST database of wheat infected by *Pst *in order to determine transcription profiles of genes involved in compatible wheat-*Pst *interaction.

**Results:**

Total RNA, extracted from susceptible infected wheat leaves harvested at 3, 5 and 8 days post inoculation (dpi), was used to create a cDNA library, from which 5,793 ESTs with high quality were obtained and clustered into 583 contigs and 2,160 singletons to give a set of 2,743 unisequences (GenBank accessions: GR302385 to GR305127). The BLASTx program was used to search for homologous genes of the unisequences in the GenBank non-redundant protein database. Of the 2,743 unisequences, 52.8% (the largest category) were highly homologous to plant genes; 16.3% to fungal genes and 30% of no-hit. The functional classification of all ESTs was established based on the database entry giving the best E-value using the Bevan's classification categories. About 50% of the ESTs were significantly homologous to genes encoding proteins with known functions; 20% were similar to genes encoding proteins with unknown functions and 30% did not have significant homology to any sequence in the database. The quantitative real-time PCR (qRT-PCR) analysis determined the transcription profiles and their involvement in the wheat-*Pst *interaction for seven of the gene.

**Conclusion:**

The cDNA library is useful for identifying the functional genes involved in the wheat-*Pst *compatible interaction, and established a new database for studying *Pst *pathogenesis genes and wheat defense genes. The transcription patterns of seven genes were confirmed by the qRT-PCR assay to be differentially expressed in wheat-*Pst *compatible and incompatible interaction.

## Background

Rust fungi are obligate biotrophic pathogens responsible for many economically important plant diseases, particularly on cereals. *Puccinia striiformis *Westend. f.sp. *tritici *Eriks. (*Pst*) causes wheat stripe rust, a devastating disease in many wheat growing areas of the world. Yield losses caused by stripe rust over a large area can be up to 50% [1-4]. Chemicals have been used to control the disease, but the most cost-effective strategy to reduce the threat of the disease is through growing resistant wheat cultivars. However, cultivars with race-specific resistance genes may become susceptible to the disease when new virulent races of the pathogen emerge. Novel methods to control the disease are needed to be developed, which requires a better understanding of the interactions between the host and pathogen, especially genes of the pathogen expressed in the course of the infection process of the compatible interaction to identify new targets for disease control. Traditionally, incompatible interactions have received much more attention than compatible interaction in order to understand the host resistance mechanism as well as to discover and use resistance genes [5,6]. Recently, studies have been reported on pathogen factors that promote compatible interactions and disease development in plant tissue [7,8].

Phenotypically, the compatible and incompatible interactions of wheat-*Pst *are obviously different. Necrotic spots or stripes can be observed after inoculation in the incompatible interaction and the ceasing development of infection hyphae at an early stage can be observed using a microscope [1,9,10]. This phenomenon is regarded as hypersensitive response (HR) and also termed programmed cell death (PCD) [11,12]. In an incompatible interaction the host tissue sacrifices the infected cell or several cells around the infection site to prevent further growth of the biotrophic pathogen. In a compatible interaction, the biotrophic fungus *Pst *[13] is armed with a complex strategy to avoid destructive effects in the host tissue during the processes of infection and spread. When urediniospores land on leaf surfaces of host plants, they germinate under suitable conditions of humidity and temperature, and the germ tubes grow toward stomata. Once germ tubes reach stomatal guard cells, they directly enter into the host leaf tissue through stomata or occasionally form appressoria on stomata [14]. From an appressorium, a penetration peg grows through the stomatal opening and develops into a vesicle in the substomatal cavity. Infection hyphae start growing in the intercellular spaces of the host tissue, and when the tip of an infection hypha reaches the cell wall of its host, a haustorial mother cell is formed. From the haustorial mother cell, a narrow haustorial neck penetrates the wall of the mesophyll cell and develops into a haustorium, which establishes an obligate biotrophic relationship with the living plant cell by redirecting the host's metabolism to meet nutritional needs of the pathogen without causing immediate death of host cells [15,16]. The lack of immediate host cell death in plant-pathogen interactions might be achieved by the pathogen through avoiding host recognition or expressing factors suppressing host defense responses. The *Pst *infection hyphae grow between host cells with haustoria in the pericellular space of host cells. It seems that specific host genes and proteins are targeted by biotrophic parasites to achieve these goals, and as such they may be considered as compatibility factors that are essential for successful pathogenesis.

In the recent years, the expressed sequence tags (ESTs) have been studied in many compatible interactions between rust fungi and their hosts. Numerous genes were reported as virulent or pathogenesis factors that may have distinct functions in the compatible plant-pathogen interaction. Hahn and Mendgen [17] isolated haustoria from *Vicia faba *leaves infected with *Uromyces fabae *and constructed a haustorium-specific cDNA library. From the library, they identified 31 in-planta induced genes (*PIGs*) that were specifically expressed in infected plants, but weakly or not expressed urediniospores. In an effort to interpret the molecular basis of the leaf rust pathogen-wheat compatible interaction, the suppression subtractive hybridization and cDNA-AFLP techniques were used [18,19]. The ESTs originating from infected wheat leaves were obtained and a proportion of them were likely of fungal origin. The differential expression analysis indicated that most of these genes were specific in rust-infected leaves, and the majority of induced genes showed homology to known *PIGs *or virulence genes in other fungi. It is predicted that these genes have common functions when the parasites infect their hosts. Recently, an EST library was constructed from fully susceptible wheat leaves inoculated with *P. graminis *f.sp. *tritici *(the wheat stem rust pathogen) and most ESTs showed similarities to fungal sequences. Quantitative RT-PCR (qRT-PCR) was used to test the expression patterns of fungal ESTs from different organisms including rust-infected leaves [20]. To identify genes expressed at a special physiological stage, a cDNA library of germinating *Phakopsora pachyrhizi *urediniospores was constructed and 52% of a total of 908 ESTs were found no significant similarity to the entries in the public protein databases [21]. The result indicates that our knowledge of genes expressed in biotrophic pathogens is still limited.

*Puccinia striiformis *f. sp. *tritici *causes severe yield losses in wheat, but the information of gene expressions in infected susceptible wheat cultivars is scanty. To obtain insights into the molecular basis of the compatible interaction between wheat and *Pst*, we report here an EST library constructed from fully susceptible wheat leaves infected with a highly virulent pathotype of *Pst*. In total, 5,793 ESTs and from which 2,743 unisequences were obtained. The qRT-PCR analysis of a number of the ESTs revealed the expression patterns of some important genes involved in the compatible interaction. The results indicate that the EST library is useful for exploring pathogen genes expressed during pathogenesis and plant genes responding to the pathogen in the infection course.

## Results and Discussion

### Construction of a cDNA library from wheat leaves challenged by Pst

**Figure 1:**
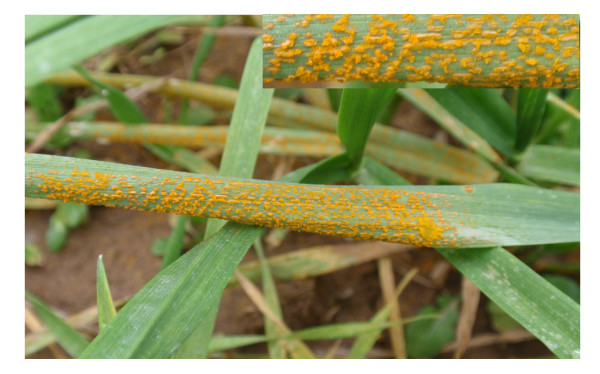
**A fully susceptible symptom of *Puccinia striiformis *f. sp. *tritici*-wheat compatible interaction**. The 15 dpi wheat leaves from wheat plants Suwon 11 inoculated with *P. striiforms *f. sp. *tritici *race CYR31, which generates a compatible interaction with heavy sporulation from rust uredia.

To expand the knowledge of potential genes involved in the wheat-*Pst *compatible interaction, a cDNA library from the wheat genotype Suwon 11 inoculated with Chinese *Pst *race CYR31 was constructed. This interaction generated severe disease symptoms (Fig. 1). Three developmental stages of *Pst *in the infected wheat leaves were selected. 1) From 1 to 3 days post inoculation (dpi). During this early infection stage, *Pst *sequentially forms the infection peg, infection hyphae, haustorial mother cell and primary haustoria that initiate the pathogen biotrophic stage. From this point, the further life cycle of the pathogen completely depends on the metabolism of the host tissue [1]. 2) The growth of *Pst *infectious hyphae takes place inside its host and continues from 3 to 7 dpi. Based on a study on leaf rust of barley, 81-85% of the volumes of the fungus were mycelium and 15-19% haustoria. Visible symptoms of infection become apparent by 7 dpi as macroscopic chlorotic patches [22]. 3) In these chlorotic patches the fungus starts sporulating 8 dpi. To representing the important infection stages, infected wheat leaves were harvested at 3, 5 and 8 dpi and used for total RNA extraction. 6.3 × 10^6 ^pfumL^-1 ^for primary library titer, 9.7 × 10^9 ^pfumL^-1 ^for amplified library titer, and 98% recombinants were obtained. Inserted cDNA lengths ranged from 0.5 kb to 2.2 kb.

### cDNA sequencing and analyses

**Figure 2:**
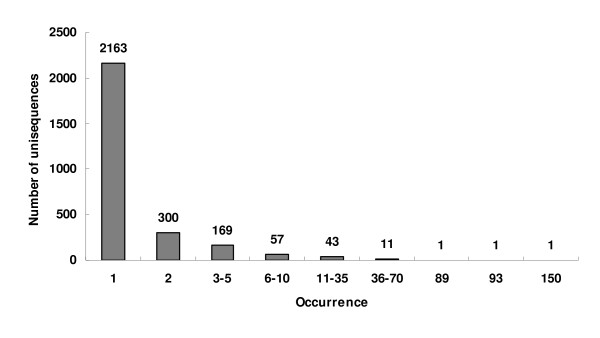
**Frequencies of EST clones derived from a *Puccinia striiformis *f. sp. *tritici*-wheat compatible interaction**. The number of ESTs is shown above the histograph for each number of occurrences. After sequencing 6,002 clones, 5,793 ESTs with high quality were obtained and clustered into 583 contigs and 2,163 singletons, resulting in 2,746 unique sequences (unisequences).

From 6,002 randomly picked clones that were sequenced from 5' end, 5,793 ESTs with high quality were obtained and clustered into 583 contigs and 2,160 singletons to give a set of 2,743 unique sequences (unisequences) (Fig. 2). EST similarity search using the BLASTx program was carried out by comparing sequences in the non-redundant protein database of the GenBank. About 50% of the ESTs showed significant matches to genes encoding proteins with known functions; 20% of the ESTs were similar to genes encoding proteins with unknown functions; and 30% of the ESTs did not have significant similarities to any sequences in the database.

In the library, 52.8% ESTs, which was the largest category, had the highest homology with plant genes (Additional file 1) 16.3% ESTs had significant homology with fungal genes (Additional file 2 and 30% had no hits (Additional file 3). Of the 812 ESTs in the no-hit category, 101 were isolated more than twice. WRIC_47 was isolated 32 times and WRIC_557 18 times. The high frequencies of these genes in the EST library might indicate their involvement in pathogenicity and virulence. In some cases, the lack of similarity to protein database entries could be due to the sequence being derived from the 5' non-translated region of the cDNA [23].

### Functional annotation and analysis

**Figure 3:**
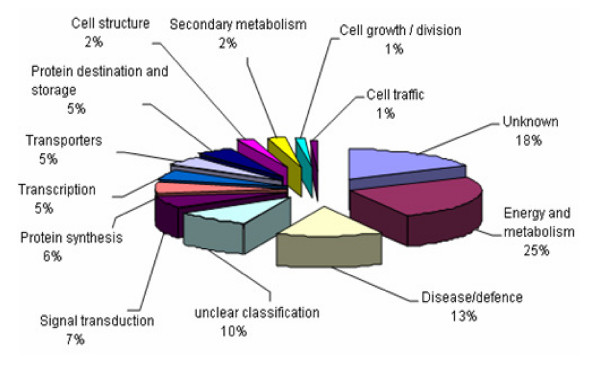
**The functional classification of ESTs from *Puccinia striiformis *f. sp. *tritici*-wheat compatible interaction with significant similarities to plant genes in the GenBank database**. The homologies of unigenes to plant genes were classified into 13 functional categories based on their cellular roles. About 26% of the ESTs showed significant homology to enzymes involved in energy and metabolism, representing the largest category. 18% ESTs showed homologies to genes of unknown function and hypothetic proteins in the Genbank database.

To conduct functional classification of the wheat-*Pst *compatible interaction, each unisequence EST was compared with the non-redundant database from the NCBI using the BLASTX algorithms [24]. The functional classification of all ESTs was established based on the database entry giving the best E-value and the categories defined by Bevan *et al*. [25]. The unigenes homologous to plant genes were classified into 13 functional categories based on the cellular roles of expressed genes (Fig. 3). The largest category counted for 26% of the classified ESTs with putative functions as enzymes of energy and metabolism. The second largest was the category of enzymes with unknown and hypothetic protein, counting for 18% of the classified ESTs. The third category was disease/defense proteins, counting for 13% of the ESTs.

### Genes homologous to those with known functions in other fungal pathogens

Among the 2,743 unisequences, 446 showed homologies to fungal genes from model fungal species, such as *Cryptococcus neoformans*, *Neurospora crassa*, *Aspergillus nidulans*, *Saccharomyces cerevisiae *and *Candida albicans*, and plant pathogenic fungi such as *Ustilago maydis*, *P. triticina*, *Magnaporthe grisea*, *Eremothecium gossypii*, *Uromyces appendiculatus *and *P. graminis*. From the fungal ESTs involved in the wheat-*Pst *compatible interaction, we identified 15 genes homologous to other rust fungal genes (Table 1).

**Table 1:** *Puccinia striiformis *f.sp. *tritici *ESTs with significant homologies to fungal pathogenicity/virulence factors

User ID	Accession No.	Copies	Length	Putative function and Species	E-value
WRIC_312	GR302696	2	951	rust transferred protein (*Uromyces striatus*)	6.00E-27
WRIC_322	GR302706	9	978	differentiation-related protein Infp (*Uromyces appendiculatus*)	1.00E-14
WRIC_348	GR302732	10	785	PIG28 (*Uromyces fabae*)	2.00E-53
WRIC_365	GR302749	2	633	alcohol dehydrogenase (*Puccinia triticina*)	6.00E-70
WRIC_406	GR302790	2	750	chitin deacetylase (*Flammulina velutipes*)	1.00E-111
WRIC_495	GR304563	7	1333	elongation factor (*Puccinia graminis*)	0.00E+00
WRIS_1741	GR303261	1	666	CAP1 (*Cryptococcus gattii*)	1.00E-36
WRIS_1608	GR303211	1	604	THI2p (*Uromyces viciae-fabae*)	8.00E-75
WRIS_1773	GR303275	1	739	actin (*Puccinia graminis*)	1.00E-114
WRIS_4517	GR304357	1	347	ATP-binding cassette transporter ABC2 (*Venturia inaequalis*)	2.00E-30
WRIS_1781	GR303282	1	643	actin (*Puccinia graminis*)	1.00E-51
WRIS_2031	GR303386	1	751	chitinase (*Puccinia triticina*)	2.00E-25
WRIS_316	GR303865	1	619	PIG1 (*Uromyces fabae*)	0.00E+00
WRIS_3246	GR303896	1	695	hypothetical class II chitin synthase (*Puccinia graminis*)	9.00E-29
WRIS_3511	GR304003	1	790	chitinase (*Puccinia triticina*)	4.00E-19
WRIS_4318	GR304280	1	524	HSS1 (*Puccinia graminis*)	0.00E+00
WRIS_574	GR304877	1	574	alcohol dehydrogenase (*Puccinia triticina*)	7.00E-82
WRIS_4003	GR304167	1	699	Ras2 (*C. neoformans*)	1.00E-74
WRIS_3617	GR304047	1	597	ABC transporter (*Candida albicans*)	2.00E-27

Three genes described as PIGs as defined by Hahn and Mendgen [17] were detected. WRIC_348 was highly homologous to *PIG28*, which was reported to be continuously up-regulated in infected broad bean leaves from 12- or 18-h infection structures and culminate in haustoria and infected leaves infected with *U. fabae*. WRIC_348, which was detected 10 times in the wheat-*Pst *compatible interaction library, is predicted to encode a protein strongly resembling peptidyl-prolyl cis-trans isomerases (PPIase) and cyclophilin. PPIase is considered to catalyze the isomerization of proline-containing peptide bonds and assists in the folding of proteins [26]. The cytoplasmic PPIase also was induced by heat shock in yeast [27]. Gene encoding cyclophilins were also specifically expressed in *U. fabae *and *P. triticina *[17,19].

Two ESTs, WRIS_316 and WRIS_1608, were similar to genes *THI1 *(*PIG1*) and *THI2 *(*PIG4*) expressed in haustoria of *U. fabae *[28]. *THI1 *and *THI2 *were found to be highly expressed in haustoria of *U. fabae*, representing approximately 5% of the haustorial mRNA and homologous to genes involved in thiamine (vitamin B1) biosynthesis [17,29]. This indicates that haustoria not only function in uptaking nutrients from plants but also involved in biosynthesis of fungal metabolites [28,30], an additional role for haustoria in the biosynthesis of fundamental metabolites that might not be provided in sufficient amounts by the host [28]. The expression patterns of *THI1 *and *THI2 *were very similar. Their transcription levels increased in germinated urediniospores *in vitro*, and were also accumulated to very high levels in haustoria and rust-infected host leaves. No expression was observed in healthy plants, urediniospores, and early infection structures of the pathogens. Also the isolation of such genes from the wheat leaves infected by *P. triticina *has been reported [19]. Our results together with the previous reports indicate that the functions of these genes are essential in obligate biotrophic rust fungi.

WRIS_2031 and WRIS_3511 were predicted to encode chitinase because of their best homology to the fungal chitinase gene of *P. triticina*. Both genes are up-regulated from 5 dpi [17,18]. Since chitin represents a main component of the cell wall of *Pst*, it may be assumed that chitinases play an essential role in cell wall synthesis in the tips of intercellularly spreading hyphae.

WRIC_312 was assembled with two ESTs and predicted to be a rust transferred protein (RTP) because of its similarity to genes in *Uromyces striatus*. *RTP *was originally described as *PIG7 *among the 31 *PIGs *isolated from the broad bean leaves infected by *U. fabae *[17]. An immuno-fluorescence staining test provided evidence that RTP protein represents a secreted protein which is transferred from the rust haustoria into the nucleus of the infected cells of *V. faba*. This was the first report demonstrating that a fungal protein was transferred to the nucleus of its host. It has been suggested that the RTP protein plays an important role in the biotrophic interaction between the parasite and its host [31]. This finding is similar to the bacterial type III secretion system which is well-known for the transfer of virulence proteins into its host cells [32].

The sequence of WRIS_1741 is similar to the gene encoding a cyclase-associated protein 1 (CAP1) from *Cryptococcus gattii*. CAP1 has three domains. The N-terminal domain binds to adenylyl cyclase and is involved in *Ras*-responsiveness [33]. The C-terminal domain binds to G-actin and strongly inhibits actin polymerization [34]. The function of the internal, proline-rich domain of CAP is still unclear. Over-expression of an Arabidopsis CAP homologue (AtCAP1) in transgenic tobacco plants reduced expansion of epidermal and mesophyll cells in leaf tissues [35,36]. A *CAP1 *gene of *Candida albicans *was identified as a virulence factor when it interacts with its host, and was required for bud-hypha transitions, filamentous growth, and regulation of cAMP levels. *C. albicans *strains with the *CAP1 *gene were virulent when they infect their mice host, but the *cap1 *mutant was avirulent [37].

WRIC_406 was homologous to the chitin deacetylase, which catalyzes deacetylation of chitin to yield chitosan. The chitin deacetylase was specifically regulated in infection structures of *U. viciae-fabae *and seems to help to counter hyphal degradation by plant chitinases [38]. More importantly, chitosan is present in hyphal cell walls of *Pst *immediately at the beginning of the plant-pathogen interaction [39]. This indicates that the gene of chitin deacetylase is transcribed specifically at the intimate contact stage of the pathogen with its host tissue. Thus, deacetylation of chitin may protect hyphal cell walls or other infection structures from degrading by plant chitinases [40].

ATP-binding cassette (ABC) transporters are membrane proteins which drive the transport of compounds over the biological membranes. WRIS_3617 and WRIS_4517 are two genes homologous to ABC transporters of *Candida albicans *and *Venturia inaequalis*, respectively. ABC transporters are known to be involved in drug resistance which has been described in filamentous fungi [41]. In plant pathogenic fungi ABC transporters may act as virulence factors which mediate secretion of pathogenicity factors or protecting the fungi against defense compounds produced by host plants [42,43]. Thus, ABC transporters may play a dual role in pathogenic fungi resulting in enhancing their virulence. These results strongly suggest that *Pst *requires the up-regulation of specific ABC transporters for pathogenesis and protection against plant defense mechanisms. Recently, the cloned *Lr34*/*Yr18 *for durable resistance to leaf rust and stripe rust in wheat was found to be a putative ABC transporter [44]. Thus, WRIS_3617 and WRIS_4517 are also likely wheat genes involved in the basal defense during the compatible interaction.

In our cDNA library, WRIS_1157 had a similar sequence to that of a *Cryptococcus neoformans *transketolase gene, which is an enzyme involved in primary carbohydrate metabolism. The finding of WRIS_1157 may provide evidence for the normal biosynthetic capabilities of rust fungi when it intrudes into the plant tissue as previously hypothesized [17].

Actin is an important framework compound and ubiquitous in various organisms. We identified the high occurrence of WRIS_1773 and WRIS_1781, which were predicted to encode actin proteins based on their high sequence similarities (E-value were 1e-144 and 1e-51, respectively) to those of *P. graminis*. This provided evidence that both *Pst *and *P. graminis *have developed common structures in susceptible wheat cultivars.

### Quantitative real-time PCR (qRT-PCR) analysis of candidate pathogenicity-related factors

**Figure 4:**
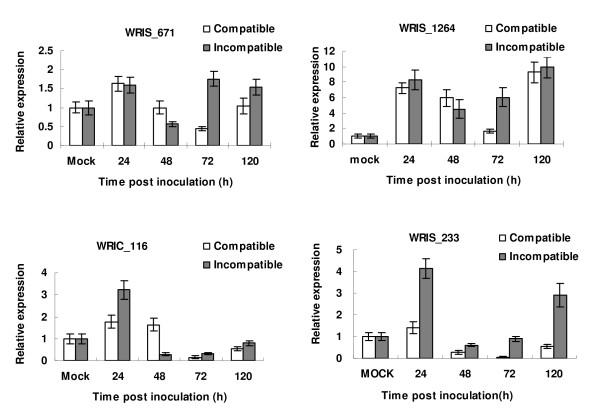
**Quantitative RT-PCR assay for the transcription levels of four cell death suppressor-related proteins in the compatible and incompatible interactions of wheat- *P. striiformis *f. sp. *tritici***. RNA derived from wheat-*P. striiformis *f. sp. *tritici. *compatible (wheat cultivar Suwon 11 inoculated with CYR31) and incompatible (wheat cultivar Suwon 11 inoculated with CYR23) interactions was extracted from infected wheat leaves harvested at 0, 24, 48, 72 and 120 hpi. The Y ax shows relative expressions as folds of the mean expression level of the mock treatment. The mock values for compatible and incompatible interactions are mean values for all time-points as there was no significant difference among the different time points.

Seven genes were selected to conduct the qRT-PCR analysis for transcription levels at different developmental and infection stages (Table 2). Four of these genes were found from wheat and three from *Pst *based on the results of the BLAST search. The four wheat genes, WRIS_671 (BAX inhibitor 1), WRIS_233 (cyclophilin), WRIS_1264 (defender against cell death 2) and WRIC_116 (putative cell death suppressor) were predicted to be related to defense against cell death at different infection stages of wheat-*Pst *interaction based on the expression patterns (Fig. 4). Bax inhibitor 1 (*BI-1*) was reported as the cell death suppressor of Bax-induced cell death in mammals and plants [45,46]. The expression of *BI-1 *gene was rapidly up-regulated during abiotic and biotic stresses [47]. The over-expression and knock-down of the *BI-1 *in *Arabidopsis *plants did not affect the plant growth under general growth conditions [48]. These results demonstrate that *BI-1 *is specifically regulated by some environmental factors. In the compatible and incompatible interactions of wheat and *Pst*, *BI-1 *had a similar transcription expression pattern. It was up-regulated at 24 hpi after inoculation with nearly equal transcription levels and then slightly decreased at 48 hpi and reached the lowest levels by 72 hpi in the compatible interaction, but decreased drastically at 48 hpi in the incompatible interaction. However, it was again up-regulated abruptly 72 hpi in the incompatible interaction.

**Table 2:** Sequences of primers used for qRT-PCR

Unigenes ID	Primer sequences(5'--3')
WRIS_671	Forward GGCTACGACTCCCTCAAGAAC
	Reverse TAGACTGGCACCGAGAACATC

WRIS_233	Forward GGGAGAGATACCAACGGAT
	Reverse CGCTTCTACAACGGAAACTA

WRIS_1264	Forward ACGCCCACGAATCTCAAG
	Reverse AGGCAAACACCAAGCACTG

WRIC_116	Forward CAGCCTGACTGGTATGGAAG
	Reverse ATGCTTCTGGCGGTCGTAG

WRIC_316	Forward TCTCTGTCCTTCTCAACTGGC
	Reverse TGGATCATAGCTTTGCATCC

WRIC_1608	Forward TCGCACCCATCAAGGAGCAT
	Reverse GCCACCCAACCAACAACCAC

WRIC_406	Forward CCATACCCACAGCGAGATT
	Reverse CCAGTTATTGCCGTTCACA

Wheat 18s RNA	Forward CTGAGAAACGGCTACCACAT
	Reverse CCCAAGGTCCAACTACGAG

*P. striiformis *actin	Forward CACACTTTCCACAACGAGC
	Reverse GGAGGCATACAAGGAGAGC

In the compatible interaction, WRIS_233 (cyclophilin) was up-regulated slightly at 24 hpi, but subsequently down-regulated, while in the incompatible interaction, the gene was drastically up-regulated at 24 and 120 hpi. These results suggest that cyclophilin has more functional activities in the incompatible interaction. In contrast, in the compatible interaction, cyclophilin was down-regulated at 24 hpi. WRIS_1264 is a defender against cell death 2 (DAD2), which is different from DAD1 at seven amino acid residues. DAD1 is a negative regulator of programmed cell death (PCD) identified as a part of the oligo saccharyltransferase (OST) complex. It has been reported also as a cell death suppressor protein, whose defect at restrictive temperature causes PCD in a hamster mutant cell line [49]. PCD results in a hypersensitive response of plants in which cells invaded by the pathogen and surrounding cells die in order to limit pathogen spread [50,51]. PCD is one of the vital characters of incompatible plant-pathogen interactions. DAD2 was highly expressed in the wheat-*Pst *interaction as two peaks during the early infection stage at 24 and 120 hpi. In the compatible interaction, it dropped to the lowest level at 72 hpi. It is unclear whether DAD modulates the process of PCD in the wheat-*Pst *compatible interaction. However, DAD2 expression at high levels at the infection stages indicates that it plays likely a crucial but so far an unknown role. Another gene (WRIC_116) was labeled as putative cell death suppressor, which doesn't have a clear function annotation. It was up-regulated at 24 and 48 hpi at high levels and down-regulated at 72 and 120 hpi in the compatible interaction, but up-regulated only at 48 hpi in the incompatible interaction. Cell death suppressor was an anti-apoptosis protein in transgenic plants and was demonstrated as *Bcl-2*, *Bcl-XL *and *Ced-9 *to provide protection from pathogens [52,53]. As a biotrophic pathogen, *Pst *intrudes into the plant tissue by avoiding damage to plant tissue in the compatible interaction, a voluntary cell death can restrict establishing infection and spreading of the pathogen.

**Figure 5:**
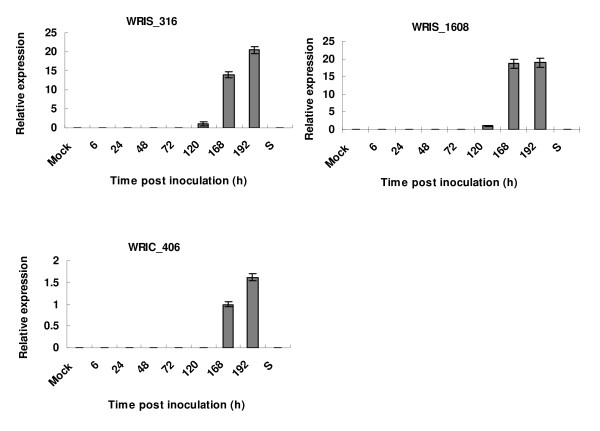
**Quantitative RT-PCR assay for the transcription levels of three ESTs with significant homologies to fungal genes in the compatible interactions of wheat- *Puccinia striiformis *f. sp. *tritici *and urediniospores**. RNA derived from compatible (wheat cultivar Suwon 11 inoculated with CYR31) interaction was extracted from infected wheat leaves harvested at 0, 6, 24, 48, 72, 120, 168, 192 hpi and urediniospores. The Y ax of the figure shows relative expressions of three fungal homologues genes compared with the mean expression level of the mock treatment. The mock values for compatible interactions are mean values for all time-points as there is no significant difference among the different time points. S, urediniospores.

The expression patterns of the three *Pst *genes are shown in Fig. 5. The sequences of WRIS_316 and WRIC_1608 were similar to the pathogenesis related proteins PIG1 (THI1p) and PIG4 (THI2p) from *U. fabae*, respectively. Two ESTs were homologous to genes involved in thiamine (vitamin B1) biosynthesis in yeast and have a very similar transcriptional pattern. These genes were reported to be mainly transcribed in haustoria and rust-infected broad bean leaves, but were not detected in urediniospores and in infection structures differentiating *in vitro *until 18 hpi [17,28]. *PIG1 *and *PIG4 *primers were designed according to the EST sequences generated from the cDNA library from wheat-*Pst *compatible interaction and the mRNA expression pattern of these two ESTs tested by qRT-PCR. Both genes were remarkably up-regulated at the later stage of *Pst *development, but no transcripts were detected in the early stage post inoculation and in urediniospores. According to the results of a previous study [54] and our findings, *PIG1 *and *PIG4 *have similar transcription patterns in the compatible rust-plant interaction and their expression begin at the stage of haustorial formation.

WRIC_406 is homologue to a chitin deacetylase gene from *Flammulina velutipes*. The enzyme plays a role in the formation of a protection compound against fungal infection s [55]. In the wheat-*Pst *compatible interaction, it was highly transcribed at the later stage of *Pst *development in wheat tissue, but no transcripts were detected in healthy plants, early stages after inoculation and in urediniospores (Fig. 5). According to the character of obligate biotrophic fungi [16], the reason might be that in the initial phase rust infection structures are not in close contact to the plant cells and expression is not triggered. When more infection hyphae and the rust haustoria are formed, a tight contact to plant cells is established and the real biotrophic phases operate. The host secrets higher activities of chitinases to degrade the chitin in fungal cell walls, however, synthesis of fungal chitin deacetylase is elicited to evade the action of host defense [38].

## Conclusion

From a total of 5,793 ESTs of high quality, 2,743 unisequences were obtained from a cDNA library of a compatible wheat-*Pst *interaction. The library is useful for identifying the functional genes involved in the wheat-*Pst *compatible interaction, and has established a new database for studying *Pst *pathogenesis genes and wheat defense genes. About 50% of the ESTs were significantly homologous to genes encoding proteins with known functions; 20% were similar to genes encoding proteins with unknown functions; and 30% did not have significant homology to any sequence in the database. 52.8% of the ESTs had the highest homology with plant genes and 16.3% had significant homology to fungal genes. The transcription patterns of seven genes were determined by qRT-PCR assays, revealing their differential expression patterns in wheat-*Pst *compatible and incompatible interaction.

## Methods

### Plant growth and inoculation

Chinese *Pst *races CYR31 and CRY23 used in this study were from the *P. striiformis *f. sp. *tritici *collection of the College of Plant Protection at Northwest A&F University. Wheat genotype Suwon 11 was grown in the growth chambers at 15-20°C. When primary leaves developed, wheat plants were inoculated separately with urediniospores of the pathotypes CYR31 and CYR23. CYR31 produces a compatible interaction and CRY23 produces an incompatible interaction on Suwon 11. The former combination was used for constructing the compatible interaction cDNA library and qRT-PCR experiments while the later combination was used only in the qRT-PCR analysis for comparison of transcriptional levels of selected genes. Post-inoculated and mock-inoculated plants were kept at high humidity in the dark for 24 h followed by the regular day-night rhythm in the growth chamber. Primary leaves were harvested at various days post-inoculation (dpi) and immediately frozen in liquid nitrogen.

### RNA extraction and cDNA library construction

Total RNA was extracted from sampled Suwon 11-CYR31 wheat leaves harvested at 3, 5 and 8 dpi using the RNeasy plant Mini kit from QIAGEN. The construction of the cDNA library was carried out using the Clontech SMART cDNA Library Construction Kit. Equal amounts of the total RNA samples from three samples were pooled as the initiating template of ss-cDNA synthesis, ds-cDNA was amplified using LD PCR and digested ds-cDNA was ligated into λTriplEx2 vector and packaged with MaxPlaxTM Lambda Packaging Extract from EPICENTRE according to the manufacturer's instructions. About 10,000 individual phage colonies were transferred to 0.5 mL micro-centrifuge tubes, containing 1 × Lambda dilution buffer supplement with 7 Dimethyl Sulphoxide (DMSO).

### cDNA sequencing and analysis

More than 6,000 clones were randomly selected, and the size of each insert was assessed by PCR amplification with the sequencing primers generated from the two ends of vector and subjected to electrophoresis using 1.5% agarose gels. Plasmid DNAs were prepared for sequencing reactions using the H.Q. & Q. plasmid mini kit. Purified plasmid DNA was sequenced from the 5' end using the Applied Biosystems (ABI) PRISM bigdye terminator kit (Perkin-Elmer) and the ABI Applied Biosystems 3700 *xl *DNA analyzer at the Biotechnology Center of Northwest A&F University (Yangling, China).

The Phred program was used to trim vector sequences and low-quality sequences from the raw sequencing data, then the processed sequences were clustered into contigs and singletons using the CAP3 software. Sequence similarity search for each unisequence was done using the BLASTX algorithm [25] to identify putative gene homologues in the non-redundant (nr) protein sequence database of the National Center for Biotechnology Information (NCBI).

### Real-time PCR

RNA from compatible (Suwon 11-CYR31) and incompatible (Suwon 11-CYR23) interactions was extracted from infected wheat leaves harvested at 0, 24, 48, 72 and 120 hpi for four plant homologue genes and 0, 6, 24, 48, 72, 120, 168, 192 hpi and urediniospores for *Pst *genes. The cDNA was synthesized from 2 μg total RNA with 500 ng of 18mer oligo-dT primers and M-MLV reverse transcriptase (Promega). Quantitative RT-PCR was performed on the iQ5 Real-Time PCR Detection System instrument (Bio-Rad), and the primers of quantitative PR-PCR were designed from the differentially expressed ESTs using the Primer 5.0 program. A 18s ribosomal RNA sequence from wheat (gi: 15982656) and an actin sequence from *P. graminis *(gi: 1703158) were selected as an endogenous control for plant genes and fungal genes, respectively, to normalize the input RNA and efficiency of reverse transcription between the samples. For quantitative RT-PCR, 3 μL of each template was used in each reaction with 0.12 μM of each primer using iQ SYBR Green Supermix (Bio-Rad). The following cycling program was used: 95°C for 60 sec, followed by 40 cycles (10 sec at 95°C, 30 sec at 55°C and 30 sec at 72°C). All products were subjected to the melting curve analysis between 55°C and 95°C to determine the specificity of the PCR reaction. Experiments included a non-template control. Quantification of the target gene was assessed using relative standard curves. The plasmid with the target gene was used to prepare standard curves that contained five serial dilutions (10^-1^, 10^-2^, 10^-3^, 10^-4 ^and 10^-5^) and gene quantity was determined by the standard curve. The correlation coefficients for the analysis of dilution curve were above 0.99.

Quantitative RT-PCR experiments were done with three biological replicates, the transcription level of target genes were expressed as endogenous control transcripts and the gene expression level in inoculated sample was compared to the average expression level of mock-inoculated sample from all time-points.

## Authors' contributions

JBM: designed experiments, analyzed data and wrote manuscript. XLH: conducted qRT-PCR and analyzed the data. XJW and ZPQ participated in EST sequencing and bioinformatics analysis, LLH: coordinated the experiments and data analyses. XMC: provided advices for experiments and revised manuscript. ZSK: conceived the project, designed the experiments and wrote manuscript. All authors read and approved the final manuscript.

## Supplementary Material

Additional file 1 **Unisequences from the compatible interaction between wheat and *Puccinia striiformis *f. sp. *tritici *showing significant similarities to plant genes in the GenBank database**. These data provided represent the original EST number and best hit. Click here for file

Additional file 2 **Unisequences from the compatible interaction between wheat and *Puccinia striiformis *f. sp. *tritici *showing significant similarities to fungi genes in the GenBank database**. These data provide the original EST number and best hit. Click here for file

Additional file 3 **Unisequences that do not have any hits in the GenBank databases**. These data provides the ESTs that do not have hits in the GenBank databases. Click here for file
