# Supplementary material for: Identification of expressed genes during compatible interaction between stripe rust (Puccinia striiformis) and wheat using a cDNA library
Source: BMC Genomics. 2009 Dec 8;10:586. doi: 10.1186/1471-2164-10-586 (PMC3087560; doi:10.1186/1471-2164-10-586)
Supplement: Additional file 1 — Unisequences from the compatible interaction between wheat and Puccinia striiformis f. sp. tritici showing significant similarities to plant genes in the GenBank database. These data provided represent the original EST number and best hit. [file 1471-2164-10-586-S1.DOC]

**Additional file 1: Unisequences from the compatible interaction between wheat and *Puccinia striiformis* f. sp. *tritici* showing significant similarities to plant genes in the GenBank database**

| **Clone.** | **Accession No.** | **Copy no.** | **Length** | **Similarity and Species** | **E-value** |
| --- | --- | --- | --- | --- | --- |
| **Disease/defence** | | | | | |
| WRIC_10 | GR302394 | 2 | 695 | Autophagy (*T. aestivum*) | 5.00E-61 |
| WRIC_13 | GR302397 | 2 | 710 | alanine aminotransferase (Deschampsia antarctica) | 1.00E-64 |
| WRIC_14 | GR302398 | 6 | 979 | MtN3-like (O. sativa) | 1.00E-85 |
| WRIC_38 | GR302422 | 2 | 805 | MtN3-like (O. sativa) | 3.00E-67 |
| WRIC_48 | GR302432 | 3 | 819 | putative heat shock protein (O. sativa) | 1.00E-07 |
| WRIC_49 | GR302433 | 2 | 709 | putative alanine aminotransferase (O. sativa) | 0.00E+00 |
| WRIC_58 | GR302442 | 3 | 848 | putative chloroplast drought-induced stress protein, 34 kD (O. sativa) | 3.00E-90 |
| WRIC_69 | GR302453 | 2 | 827 | putative MtN19 (O. sativa) | 1.00E-103 |
| WRIC_82 | GR302466 | 2 | 552 | subtilisin-chymotrypsin inhibitor 2（Hordeum vulgare） | 1.00E-26 |
| WRIC_116 | GR302500 | 2 | 699 | Putative cell death suppressor protein (O. sativa) | 6.00E-49 |
| WRIC_118 | GR302502 | 2 | 606 | putative ethylene-responsive transcriptional coactivator (O. sativa) | 4.00E-67 |
| WRIC_126 | GR302510 | 4 | 539 | hydrophobic polypeptide (O. sativa) | 9.00E-23 |
| WRIC_134 | GR302518 | 2 | 661 | putative wound inductive gene (O. sativa) | 3.00E-41 |
| WRIC_129 | GR302513 | 4 | 660 | thioredoxin h (Medicago sativa) | 5.00E-29 |
| WRIC_163 | GR302547 | 2 | 798 | translationally controlled tumor protein (T. aestivum) | 1.00E-84 |
| WRIC_164 | GR302548 | 2 | 907 | aminopropyl transferase (O. sativa) | 1.00E-145 |
| WRIC_178 | GR302562 | 3 | 758 | glutathione S-transferase 2 (O. sativa) | 5.00E-99 |
| WRIC_179 | GR302563 | 2 | 427 | thioredoxin H2 (Ipomoea batatas) | 2.00E-07 |
| WRIC_189 | GR302573 | 2 | 692 | putative cellulose synthase catalytic subunit（H. vulgare） | 7.00E-90 |
| WRIC_196 | GR302580 | 2 | 666 | putative tumor differentially expressed protein 1 (O. sativa) | 8.00E-56 |
| WRIC_237 | GR302621 | 2 | 673 | putative thioredoxin peroxidase 1 (O. sativa) | 1.00E-46 |
| WRIC_243 | GR302627 | 2 | 777 | putative monodehydroascorbate reductase (O. sativa) | 8.00E-82 |
| WRIC_250 | GR302634 | 2 | 810 | putative root border cell-specific protein (O. sativa) | 8.00E-73 |
| WRIC_253 | GR302637 | 2 | 578 | catalase 1（H. vulgare） | 1.00E-55 |
| WRIC_259 | GR302643 | 2 | 549 | MPI-maize protease inhibitor (Tripsacum dactyloides) | 4.00E-17 |
| WRIC_314 | GR302698 | 2 | 1103 | Glutathione S-transferase 2 (T. aestivum) | 0.00E+00 |
| WRIC_316 | GR302700 | 4 | 587 | Metallothionein-like protein 1 (T. aestivum) | 3.00E-11 |
| WRIC_329 | GR302713 | 2 | 725 | glutathione S-transferase 2 (O. sativa) | 1.00E-104 |
| WRIC_341 | GR302725 | 6 | 645 | cysteine proteinase inhibitor (T. aestivum) | 7.00E-61 |
| WRIC_345 | GR302729 | 3 | 877 | Superoxide dismutase [Cu-Zn] (Zea mays) | 1.00E-72 |
| WRIC_369 | GR302753 | 3 | 545 | WIR1A protein (T. aestivum) | 1.00E-34 |
| WRIC_376 | GR302760 | 2 | 386 | auxin-induced putative CP12 domain-containing protein (Arachishypogaea) | 3.00E-12 |
| WRIC_398 | GR302782 | 2 | 507 | Catalase-1 (T. aestivum) | 3.00E-35 |
| WRIC_402 | GR302786 | 10 | 677 | cytochrome P450 like (Nicotiana tabacum) | 5.00E-21 |
| WRIC_414 | GR302798 | 2 | 888 | putative LytB protein (O. sativa) | 6.00E-64 |
| WRIC_422 | GR302806 | 2 | 729 | thioredoxin family Trp26-like protein (O. sativa) | 0.00E+00 |
| WRIC_426 | GR302810 | 3 | 905 | stress responsive protein (T. aestivum) | 1.00E-116 |
| WRIC_446 | GR302830 | 3 | 877 | putative heat shock protein (O. sativa) | 3.00E-80 |
| WRIC_454 | GR302838 | 3 | 773 | putative calreticulin (O. sativa) | 2.00E-38 |
| WRIC_466 | GR302850 | 3 | 554 | Hydrophobic protein OSR8 (O. sativa) | 2.00E-21 |
| WRIC_483 | GR302867 | 5 | 593 | Metallothionein-like protein 1 (T. aestivum) | 3.00E-11 |
| WRIC_492 | GR302876 | 3 | 653 | cysteine proteinase inhibitor (T. aestivum) | 3.00E-63 |
| WRIC_464 | GR302848 | 2 | 491 | Ferredoxin-thioredoxin reductase | 5.00E-33 |
| WRIC_527 | GR302911 | 2 | 563 | WIR1B protein (T. aestivum) | 8.00E-06 |
| WRIC_529 | GR302913 | 2 | 580 | ozone-responsive stress-related protein-like (O. sativa) | 2.00E-29 |
| WRIC_535 | GR302919 | 2 | 811 | Thioredoxin M-type, chloroplast precursor (T. aestivum) | 4.00E-98 |
| WRIC_572 | GR302956 | 2 | 690 | putative MtN19 (O. sativa) | 3.00E-66 |
| WRIS_1063 | GR302994 | 1 | 649 | putative auxin-induced protein (O. sativa) | 5.00E-57 |
| WRIS_1067 | GR302996 | 1 | 381 | cyclophilin A-1 (T. aestivum) | 2.00E-06 |
| WRIS_1083 | GR303005 | 1 | 356 | putative leucine-rich repeat protein (O. sativa) | 5.00E-16 |
| WRIS_1272 | GR303080 | 1 | 511 | Hydrophobic protein OSR8 (O. sativa) | 2.00E-22 |
| WRIS_1264 | GR303076 | 1 | 613 | Defender against cell death 2 (DAD-2) （H. vulgare） | 9.00E-55 |
| WRIS_1273 | GR303081 | 1 | 361 | putative cytochrome P-450 like protein (O. sativa) | 1.00E-22 |
| WRIS_1282 | GR303086 | 1 | 660 | putative glutathione S-transferase (O. sativa) | 4.00E-59 |
| WRIS_1298 | GR303089 | 11 | 435 | putative LytB protein (O. sativa) | 9.00E-24 |
| WRIS_1275 | GR303082 | 1 | 721 | putative L-ascorbate peroxidase (O. sativa) | 3.00E-39 |
| WRIS_1335 | GR303106 | 1 | 300 | CONSTANS interacting protein 3 (Lycopersicon esculentum) | 1.00E-14 |
| WRIS_1385 | GR303128 | 1 | 640 | glutathione-S-transferase 19E50 (T. aestivum) | 1.00E-80 |
| WRIS_1419 | GR303140 | 1 | 657 | thioredoxin h (Medicago sativa) | 4.00E-29 |
| WRIS_155 | GR303194 | 1 | 496 | chitinase IV precursor (T. aestivum) | 3.00E-50 |
| WRIS_1631 | GR303225 | 1 | 378 | Hydroxyacylglutathione hydrolase (O. sativa) | 8.00E-18 |
| WRIS_1642 | GR303229 | 1 | 549 | glutathione S-transferase (T. aestivum) | 3.00E-70 |
| WRIS_1644 | GR303230 | 1 | 511 | heat shock protein 90 (T. aestivum) | 2.00E-53 |
| WRIS_1680 | GR303236 | 1 | 356 | putative pathogen induced protein 2-4 (O. sativa) | 3.00E-23 |
| WRIS_1702 | GR303247 | 1 | 341 | oxo-phytodienoic acid reductase (O. sativa) | 4.00E-37 |
| WRIS_1721 | GR303254 | 1 | 338 | putative auxin-induced protein | 3.00E-10 |
| WRIS_1790 | GR303288 | 1 | 688 | putative latex protein allergen (O. sativa) | 3.00E-55 |
| WRIS_1793 | GR303291 | 1 | 744 | putative cytochrome P450(Lolium rigidum) | 1.00E-111 |
| WRIS_180 | GR303295 | 1 | 681 | MatE family protein (O. sativa) | 2.00E-64 |
| WRIS_1834 | GR303308 | 1 | 424 | harpin binding protein 1 (T. aestivum) | 2.00E-45 |
| WRIS_1857 | GR303317 | 1 | 672 | Chalcone synthase 1 (Naringenin-chalcone synthase 1) | 2.00E-65 |
| WRIS_1874 | GR303326 | 1 | 439 | Zinc knuckle family protein (O. sativa) | 3.00E-27 |
| WRIS_1902 | GR303338 | 1 | 570 | metallothionein-like protein type 3（H. vulgare） | 3.00E-18 |
| WRIS_1970 | GR303364 | 1 | 678 | peroxidase 4 (T. aestivum) | 2.00E-72 |
| WRIS_2135 | GR303429 | 1 | 516 | putative glutathione S-transferase (O. sativa) | 3.00E-44 |
| WRIS_2180 | GR303443 | 1 | 456 | low temperature and salt responsive protein-like (O. sativa) | 2.00E-09 |
| WRIS_2304 | GR303495 | 1 | 566 | WIR1A protein (T. aestivum) | 6.00E-02 |
| WRIS_2318 | GR303500 | 1 | 442 | germin-like protein (T. aestivum) | 2.00E-38 |
| WRIS_2337 | GR303507 | 1 | 736 | putative negatively light-regulated protein (O. sativa) | 2.00E-48 |
| WRIS_233 | GR303508 | 1 | 482 | Cyclophilin (T. aestivum) | 1.00E-43 |
| WRIS_2346 | GR303512 | 1 | 455 | pathogenesis-related protein 10（H. vulgare） | 7.00E-21 |
| WRIS_2349 | GR303514 | 1 | 577 | cell death-related protein (O. sativa) | 6.00E-37 |
| WRIS_2425 | GR303549 | 1 | 724 | putative senescence-associated protein (O. sativa) | 1.00E-33 |
| WRIS_247 | GR303570 | 1 | 644 | immunophilin (Zea mays) | 5.00E-55 |
| WRIS_2592 | GR303615 | 1 | 497 | fibrillin-like protein (O. sativa) | 3.00E-24 |
| WRIS_2621 | GR303625 | 11 | 674 | putative glutathione S-transferase OsGSTT1 (O. sativa) | 3.00E-74 |
| WRIS_2660 | GR303637 | 1 | 737 | Ferredoxin-3, chloroplast precursor (Z. mays) | 9.00E-59 |
| WRIS_269 | GR303659 | 1 | 706 | Thioredoxin M-type, chloroplast precursor(T. aestivum) | 6.00E-99 |
| WRIS_2701 | GR303660 | 1 | 735 | Elicitor-responsive protein 3 (O. sativa) | 2.00E-57 |
| WRIS_2726 | GR303669 | 1 | 713 | wound-responsive protein-like (O. sativa) | 1.00E-43 |
| WRIS_2728 | GR303671 | 1 | 456 | iron/ascorbate-dependent oxidoreductase（H. vulgare） | 2.00E-53 |
| WRIS_2742 | GR303679 | 1 | 596 | putative tumor differentially expressed protein 1 (O. sativa) | 1.00E-63 |
| WRIS_277 | GR303695 | 1 | 655 | thioredoxin M-like (O. sativa) | 5.00E-30 |
| WRIS_2782 | GR303696 | 1 | 447 | IAA1 protein (T. aestivum) | 2.00E-28 |
| WRIS_2784 | GR303698 | 1 | 685 | WIR1A protein (T. aestivum) | 9.00E-23 |
| WRIS_2890 | GR303739 | 1 | 363 | putative apoptosis-related protein (O. sativa) | 3.00E-07 |
| WRIS_2899 | GR303744 | 1 | 415 | universal stress protein（H. vulgare） | 1.00E-20 |
| WRIS_2944 | GR303764 | 1 | 458 | cold acclimation protein COR413-TM1(T. aestivum) | 5.00E-40 |
| WRIS_2972 | GR303776 | 1 | 512 | cyclophilin A-1 (T. aestivum) | 3.00E-44 |
| WRIS_3035 | GR303807 | 1 | 692 | putative disease resistance protein (O. sativa) | 8.00E-46 |
| WRIS_3069 | GR303823 | 1 | 668 | putative glutathione S-transferase (O. sativa) | 4.00E-56 |
| WRIS_3073 | GR303825 | 1 | 514 | putative stress-induced protein sti1 (O. sativa) | 3.00E-47 |
| WRIS_3105 | GR303840 | 1 | 675 | Macrophage migration inhibitory factor family protein (O. sativa) | 2.00E-55 |
| WRIS_3120 | GR303849 | 1 | 568 | Two-component response regulator ARR8 (O. sativa) | 2.00E-13 |
| WRIS_3233 | GR303891 | 1 | 746 | Thioredoxin H-type (T. aestivum) | 2.00E-11 |
| WRIS_3254 | GR303899 | 1 | 758 | Superoxide dismutase 1 (O. sativa) | 3.00E-75 |
| WRIS_3322 | GR303927 | 1 | 788 | putative Glucan 1,3-beta-glucosidase precursor (O. sativa) | 1.00E-65 |
| WRIS_3332 | GR303931 | 1 | 703 | cytochrome P450 (T. aestivum) | 1.00E-129 |
| WRIS_3501 | GR304001 | 1 | 685 | putative elicitor-inducible cytochrome P450 (O. sativa) | 1.00E-94 |
| WRIS_3546 | GR304020 | 1 | 648 | putative Cf2/Cf5 disease resistance protein (O. sativa) | 3.00E-56 |
| WRIS_3829 | GR304119 | 1 | 427 | thioredoxin h-like protein（H. vulgare） | 9.00E-04 |
| WRIS_3921 | GR304148 | 1 | 521 | translationally controlled tumor protein (T. aestivum) | 2.00E-71 |
| WRIS_3974 | GR304160 | 1 | 535 | putative Avr9/Cf-9 rapidly elicited protein 276 (O. sativa) | 1.00E-06 |
| WRIS_4007 | GR304168 | 1 | 230 | beta-1,3-glucanase precursor (T. aestivum) | 1.00E-12 |
| WRIS_4018 | GR304173 | 1 | 348 | beta-1,3-glucanase precursor (T. aestivum) | 4.00E-24 |
| WRIS_4034 | GR304175 | 1 | 446 | universal stress protein/early nodulin ENOD18-like | 1.00E-43 |
| WRIS_4043 | GR304179 | 1 | 676 | heat shock protein 90 (O. sativa) | 7.00E-60 |
| WRIS_4069 | GR304184 | 1 | 578 | putative 4-coumarate-CoA ligase (O. sativa) | 7.00E-41 |
| WRIS_4081 | GR304189 | 1 | 640 | putative ethylene-responsive transcriptional coactivator | 9.00E-67 |
| WRIS_4095 | GR304196 | 1 | 522 | Aquaporin（H. vulgare） | 1.00E-51 |
| WRIS_4120 | GR304208 | 1 | 645 | callose synthase-like protein (O. sativa) | 6.00E-61 |
| WRIS_415 | GR304226 | 1 | 709 | disease resistance protein-like (O. sativa) | 1.00E-32 |
| WRIS_4171 | GR304231 | 1 | 585 | putative resistance protein（Triticum monococcum） |  |
| WRIS_4370 | GR304301 | 1 | 382 | Peroxiredoxin (O. sativa) | 6.00E-29 |
| WRIS_4386 | GR304306 | 1 | 514 | putative auxin-responsive factor (ARF1) (O. sativa) | 2.00E-24 |
| WRIS_4405 | GR304316 | 1 | 498 | putative heat shock factor binding protein (O. sativa) | 5.00E-37 |
| WRIS_4486 | GR304345 | 1 | 633 | Cu/Zn superoxide dismutase (T. aestivum) | 1.00E-54 |
| WRIS_44 | GR304351 | 1 | 488 | thylakoid-bound ascorbate peroxidase (T. aestivum) | 4.00E-52 |
| WRIS_4705 | GR304430 | 1 | 493 | Mitogen-activated protein kinase 2 (MAP kinase 2) (O. sativa) | 2.00E-27 |
| WRIS_4647 | GR304415 | 1 | 657 | manganese superoxide dismutase (T. aestivum) | 9.00E-86 |
| WRIS_486 | GR304520 | 1 | 409 | probable light-induced protein (T. aestivum) | 2.00E-14 |
| WRIS_4859 | GR304511 | 1 | 578 | cold acclimation protein WCOR413-like protein beta form (T. aestivum) | 4.00E-45 |
| WRIS_4914 | GR304545 | 1 | 510 | ascorbate peroxidase（H. vulgare） | 3.00E-27 |
| WRIS_5025 | GR304592 | 1 | 421 | Phosphomethylpyrimidine kinase/thiamin-phosphate pyrophosphorylase（Z. mays） | 4.00E-27 |
| WRIS_5040 | GR304601 | 1 | 725 | thioredoxin peroxidase（Secale cereale） | 2.00E-94 |
| WRIS_5179 | GR304659 | 1 | 831 | heat shock protein 90 (T. aestivum) | 6.00E-86 |
| WRIS_5180 | GR304660 | 1 | 715 | putative early-responsive to dehydration stress protein (O. sativa) | 6.00E-76 |
| WRIS_5317 | GR304712 | 1 | 650 | putative disease resistance gene (O. sativa) | 8.00E-26 |
| WRIS_5371 | GR304731 | 1 | 524 | beta-1,3-glucanase precursor (T. aestivum) | 5.00E-55 |
| WRIS_5374 | GR304732 | 1 | 645 | nonfunctional Rpg1（H. vulgare） | 4.00E-09 |
| WRIS_5453 | GR304764 | 1 | 772 | putative heat-shock protein (O. sativa) |  |
| WRIS_5462 | GR304765 | 1 | 421 | peroxisome type ascorbate peroxidase（H. vulgare） | 1.00E-70 |
| WRIS_5511 | GR304788 | 1 | 639 | Metallothionein-like protein 1 (T. aestivum) | 4.00E-11 |
| WRIS_5610 | GR304826 | 1 | 679 | glutathione synthetase (T. aestivum) | 3.00E-90 |
| WRIS_5657 | GR304848 | 1 | 724 | Glutathione S-transferase (GST class-zeta) (T. aestivum) | 1.00E-112 |
| WRIS_5659 | GR304850 | 1 | 548 | putative iron-stress related protein (O. sativa) | 2.00E-19 |
| WRIS_5705 | GR304862 | 1 | 534 | Salt stress-induced hydrophobic peptide ESI3 | 4.00E-19 |
| WRIS_589 | GR304948 | 1 | 440 | Glutathione S-transferase 2 (GST class-phi) (T. aestivum) | 1.00E-73 |
| WRIS_5971 | GR304975 | 1 | 441 | chitinase（H. vulgare） | 1.00E-49 |
| WRIS_5982 | GR304980 | 1 | 400 | Thaumatin-like protein PWIR2 precursor (T. aestivum) | 4.00E-23 |
| WRIS_5989 | GR304984 | 1 | 705 | cytosolic glutathione peroxidase (T. aestivum) | 1.00E-90 |
| WRIS_623 | GR305000 | 1 | 603 | monodehydroascorbate reductase（H. vulgare） | 1.00E-92 |
| WRIS_680 | GR305016 | 1 | 510 | P450 monooxygenase（H. vulgare） | 2.00E-87 |
| WRIS_717 | GR305028 | 1 | 240 | putative peroxisome assembly protein 2 (O. sativa) | 1.00E-24 |
| WRIS_718 | GR305029 | 1 | 435 | heat shock factor-binding protein 1(Z. mays) | 1.00E-08 |
| WRIS_807 | GR305054 | 1 | 582 | Metallothionein-like protein 1 (T. aestivum) | 3.00E-11 |
| WRIS_798 | GR305050 | 1 | 318 | glutathione transferase (T. aestivum) | 6.00E-10 |
| WRIS_956 | GR305107 | 1 | 656 | putative glutathione transferase (O. sativa) | 1.00E-95 |
| WRIS_958 | GR305109 | 1 | 377 | glutathione S-transferase (T. aestivum) | 3.00E-21 |
| WRIS_1910 | GR303342 | 11 | 559 | putative elicitor inducible beta-1,3-glucanase (O. sativa) | 7.00E-42 |
| WRIS_2274 | GR303480 | 1 | 330 | thaumatin -like protein (T. aestivum) | 2.00E-15 |
| WRIC_85 | GR302469 | 2 | 512 | multidomain cystatin (T. aestivum) | 3.00E-39 |
| WRIS_671 | GR305011 | 1 | 720 | BAX inhibitor 1（H. vulgare） | 0.00E+00 |
| **Energy and metabolism** | | | | | |
| WRIC_23 | GR302407 | 16 | 764 | photosystem II protein D1（Saccharum hybrid） | 1.00E-126 |
| WRIC_28 | GR302412 | 18 | 1185 | putative ATPase I subunit from chromosome 10 chloroplast insertion (O. sativa) | 5.00E-21 |
| WRIC_35 | GR302419 | 10 | 617 | sphosphate caAT Ribulose birboxylase small chain clone 512 (T. aestivum) | 5.00E-58 |
| WRIC_44 | GR302428 | 4 | 1306 | phosphoribulokinase (T. aestivum) | 1.00E-172 |
| WRIC_46 | GR302430 | 2 | 787 | putative Citrate synthase (O. sativa) | 9.00E-82 |
| WRIC_52 | GR302436 | 3 | 604 | Photosystem II reaction center protein H (T. aestivum) | 1.00E-33 |
| WRIC_55 | GR302439 | 8 | 616 | Ribulose bisphosphate carboxylase small chain (T. aestivum) | 6.00E-48 |
| WRIC_62 | GR302446 | 29 | 1039 | putative photosystem II 10K protein（Oryzasativa） | 4.00E-48 |
| WRIC_68 | GR302452 | 7 | 766 | Carbonic anhydrase (O. sativa) | 6.00E-80 |
| WRIC_76 | GR302460 | 11 | 892 | cytochrome b6 (O. sativa) | 1.00E-121 |
| WRIC_77 | GR302461 | 20 | 2262 | ATP synthase CF1 beta chain (T. aestivum) |  |
| WRIC_78 | GR302462 | 4 | 687 | ATP synthase beta subunit (T. aestivum) | 9.00E-60 |
| WRIC_83 | GR302467 | 3 | 724 | photosystem II protein D1（Saccharum hybrid cultivar） | 1.00E-123 |
| WRIC_86 | GR302470 | 21 | 845 | PSII inhibitor resistant D1 protein（Bromus tectorum） | 1.00E -137 |
| WRIC_89 | GR302473 | 66 | 583 | ribulose-1,5-bisphosphate carboxylase/oxygenase large subunit (T. aestivum) | 1.00E-78 |
| WRIC_98 | GR302482 | 55 | 1212 | photosystem II protein D1（Typha latifolia） | 0.00E+00 |
| WRIC_106 | GR302490 | 2 | 833 | putative glycine decarboxylase subunit (T. aestivum) | 9.00E-82 |
| WRIC_109 | GR302493 | 2 | 327 | Photosystem I reaction center subunit III (O. sativa) | 9.00E-08 |
| WRIC_120 | GR302504 | 2 | 685 | chlorophyll a/b-binding protein precursor（H. vulgare） | 1.00E-81 |
| WRIC_122 | GR302506 | 3 | 462 | plastocyanin precursor（H. vulgare） | 7.00E-50 |
| WRIC_130 | GR302514 | 2 | 523 | Glyceraldehyde-3-phosphate dehydrogenase | 1.00E-48 |
| WRIC_133 | GR302517 | 9 | 389 | putative glycolate oxidase (O. sativa) | 8.00E-17 |
| WRIC_138 | GR302522 | 2 | 906 | putative succinyl-CoA ligase (O. sativa) | 2.00E-98 |
| WRIC_139 | GR302523 | 11 | 625 | ribulose-1,5-bisphosphate carboxylase/oxygenase small subunit (T. aestivum) | 3.00E-68 |
| WRIC_149 | GR302533 | 2 | 725 | cytochrome b6/f complex subunit 4 （Lactuca sativa） | 3.00E-94 |
| WRIC_155 | GR302539 | 4 | 1209 | photosystem II 10 kDa polypeptide (O. sativa) | 7.00E-46 |
| WRIC_156 | GR302540 | 10 | 788 | ribulose-1,5-bisphosphate carboxylase/oxygenase large subunit（H. vulgare） | 2.00E-70 |
| WRIC_158 | GR302542 | 2 | 812 | pollen 2-phosphoglycerate dehydrogenase 2 precursor（Cynodondactylon） | 5.00E-88 |
| WRIC_159 | GR302543 | 15 | 618 | putative glycolate oxidase (O. sativa) | 2.00E-16 |
| WRIC_165 | GR302549 | 2 | 720 | cytochrome b561 （Zea mays） | 6.00E-81 |
| WRIC_167 | GR302551 | 2 | 369 | Chlorophyll a-b binding protein, chloroplast precursor (O. sativa) | 5.00E-61 |
| WRIC_174 | GR302558 | 5 | 1081 | putative photosystem II protein reaction center W (O. sativa) | 1.00E-54 |
| WRIC_182 | GR302566 | 75 | 1519 | ribulose 1,5-bisphosphate carboxylase/oxygenase large chain (T. aestivum) | 0.00E+00 |
| WRIC_186 | GR302570 | 3 | 552 | Photosystem I reaction center subunit II | 4.00E-60 |
| WRIC_191 | GR302575 | 34 | 927 | photosystem II M protein (O. sativa) | 2.00E-22 |
| WRIC_195 | GR302579 | 3 | 462 | putative 33kDa oxygen evolvingprotein of photosystem II (O. sativa) | 1.00E-33 |
| WRIC_198 | GR302582 | 2 | 1097 | putative transaldolase (O. sativa) | 1.00E -129 |
| WRIC_208 | GR302592 | 3 | 502 | Cytochrome c oxidase polypeptide Vc | 4.00E-27 |
| WRIC_210 | GR302594 | 2 | 394 | chlorophyll a/b binding protein（Brassica oleracea） | 4.00E-24 |
| WRIC_218 | GR302602 | 5 | 344 | putative rubisco small subunit (T. aestivum) | 8.00E-25 |
| WRIC_220 | GR302604 | 4 | 909 | Carbonic anhydrase, | 1.00E -114 |
| WRIC_222 | GR302606 | 12 | 1008 | photosystem II protein D1 (T. aestivum) | 1.00E -162 |
| WRIC_227 | GR302611 | 3 | 932 | Cytochrome b6-f complex iron-sulfur subunit | 1.00E -119 |
| WRIC_235 | GR302619 | 2 | 365 | glyceraldehyde-3-phosphate dehydrogenase | 4.00E-08 |
| WRIC_238 | GR302622 | 12 | 699 | ribulose-1,5-bisphosphate carboxylase/oxygenase small subunit (T. aestivum) | 1.00E-76 |
| WRIC_240 | GR302624 | 3 | 1167 | glyoxalase I (O. sativa) | 1.00E -156 |
| WRIC_247 | GR302631 | 2 | 947 | putative photosystem II 10K protein（Oryzasativa） | 5.00E-37 |
| WRIC_249 | GR302633 | 7 | 1385 | ATP synthase CF0 A chain (O. sativa) | 1.00E -130 |
| WRIC_252 | GR302636 | 2 | 859 | ribulose-bisphosphate carboxylase (EC 4.1.1.39) small chainprecursor (O. sativa) | 1.00E-79 |
| WRIC_255 | GR302639 | 3 | 660 | Photosystem I reaction center subunit IV (O. sativa) | 1.00E-39 |
| WRIC_267 | GR302651 | 2 | 771 | ribulose-1,5-bisphosphate carboxylase/oxygenase small subunit (T. aestivum) | 3.00E-87 |
| WRIC_268 | GR302652 | 2 | 670 | putative rubisco small subunit (T. aestivum) | 2.00E-54 |
| WRIC_270 | GR302654 | 7 | 739 | Ribulose bisphosphate carboxylase small chain (O. sativa) | 8.00E-84 |
| WRIC_284 | GR302668 | 4 | 1520 | putative transketolase (O. sativa) | 0.00E+00 |
| WRIC_287 | GR302671 | 4 | 596 | Cytochrome c oxidase polypeptide Vc（Hordeum vulgare） | 2.00E-26 |
| WRIC_293 | GR302677 | 2 | 1032 | chlorophyll a/b-binding protein (T. aestivum) | 1.00E -147 |
| WRIC_301 | GR302685 | 6 | 832 | photosystem II protein K (T. aestivum) | 4.00E-28 |
| WRIC_337 | GR302721 | 20 | 677 | ribulose-1,5-bisphosphate carboxylase/oxygenase small subunit (T. aestivum) | 2.00E-75 |
| WRIC_339 | GR302723 | 2 | 778 | putative cytochrome B5 (O. sativa) | 1.00E-66 |
| WRIC_354 | GR302738 | 3 | 450 | Photosystem I reaction center subunit III, chloroplast precursor（maize） | 9.00E-27 |
| WRIC_359 | GR302743 | 35 | 1704 | ribulose 1,5-bisphosphate carboxylase activase isoform 1（H. vulgare） | 0.00E+00 |
| WRIC_361 | GR302745 | 3 | 1499 | photosystem II 47 kDa protein(T. aestivum) | 0.00E+00 |
| WRIC_367 | GR302751 | 18 | 794 | photosystem II protein D1（Saccharum hybrid cultivar SP-80-3280）(O. sativa) | 1.00E -137 |
| WRIC_371 | GR302755 | 3 | 586 | Ribulose bisphosphate carboxylase small chain (T. aestivum) | 4.00E-58 |
| WRIC_374 | GR302758 | 4 | 667 | Photosystem I reaction center subunit psaK, chloroplast precursor（H. vulgare） | 1.00E-54 |
| WRIC_386 | GR302770 | 3 | 657 | photosystem II 10 kDa polypeptide (O. sativa) | 1.00E-46 |
| WRIC_407 | GR302791 | 2 | 1152 | mitochondrial ATP synthase precursor (T. aestivum) | 1.00E -111 |
| WRIC_409 | GR302793 | 4 | 1054 | photosystem I subunit VII (O. sativa) | 5.00E-44 |
| WRIC_427 | GR302811 | 3 | 529 | putative chloroplast chlorophyll A-B binding protein type II (Sorghum bicolor) | 7.00E-30 |
| WRIC_429 | GR302813 | 15 | 1553 | photosystem I P700 apoprotein A2 (T. aestivum) | 0.00E+00 |
| WRIC_436 | GR302820 | 7 | 784 | photosystem II protein D1 (T. aestivum) | 0.00E+00 |
| WRIC_438 | GR302822 | 4 | 750 | photosystem II protein D1(Saccharum hybrid cultivar SP-80-3280) | 0.00E+00 |
| WRIC_441 | GR302825 | 10 | 1653 | ribulose 1,5-bisphosphate carboxylase activase isoform 1（H. vulgare） | 0.00E+00 |
| WRIC_452 | GR302836 | 10 | 1697 | ribulose 1,5-bisphosphate carboxylase activase isoform 1（H. vulgare） | 0.00E+00 |
| WRIC_461 | GR302845 | 38 | 1302 | Photosystem II 44 kDa reaction center protein (P6 protein) （H. vulgare） | 0.00E+00 |
| WRIC_465 | GR302849 | 2 | 772 | digalactosyldiacylglycerol synthase 1, putative, expressed (O. sativa) | 1.00E -116 |
| WRIC_472 | GR302856 | 2 | 449 | ATP synthase D chain, mitochondrial, putative (Solanum demissum) | 3.00E-21 |
| WRIC_475 | GR302859 | 42 | 747 | photosystem II protein D1 (T. aestivum) | 1.00E -110 |
| WRIC_481 | GR302865 | 2 | 476 | Chlorophyll a-b binding protein 25, chloroplast precursor(Petunia sp) | 1.00E-33 |
| WRIC_485 | GR302869 | 2 | 1151 | cryptochrome 1b（H. vulgare） | 0.00E+00 |
| WRIC_490 | GR302874 | 2 | 655 | putative glycine decarboxylase subunit (T. aestivum) | 3.00E-81 |
| WRIC_491 | GR302875 | 8 | 420 | photosystem II protein D1（Ranunculus macranthus） | 2.00E-61 |
| WRIC_493 | GR302877 | 14 | 1053 | photosystem II 10 kDa polypeptide (O. sativa) | 2.00E-46 |
| WRIC_497 | GR302881 | 45 | 783 | ribulose-1,5-bisphosphate carboxylase/oxygenase large subunit (T. aestivum) | 1.00E -124 |
| WRIC_500 | GR302884 | 48 | 965 | photosystem II protein D1 (T. aestivum) | 1.00E -170 |
| WRIC_501 | GR302885 | 4 | 1158 | cytochrome b559 alpha chain (O. sativa) | 5.00E-51 |
| WRIC_503 | GR302887 | 5 | 876 | ferredoxin-NADP(H) oxidoreductase (T. aestivum) | 1.00E-79 |
| WRIC_506 | GR302890 | 2 | 835 | putative succinate dehydrogenase flavoprotein alpha subunit (O. sativa) | 5.00E-76 |
| WRIC_511 | GR302895 | 2 | 367 | chlorophyll a/b binding protein（Brassica oleracea） | 1.00E-28 |
| WRIC_524 | GR302908 | 35 | 575 | photosystem II protein D1 (T. aestivum) | 4.00E-94 |
| WRIC_528 | GR302912 | 93 | 495 | photosystem II protein D1 (T. aestivum) | 6.00E-70 |
| WRIC_531 | GR302915 | 8 | 640 | photosystem II 10 kDa polypeptide (O. sativa) | 2.00E-46 |
| WRIC_541 | GR302925 | 5 | 651 | Oxygen-evolving enhancer protein 2 (T. aestivum) | 3.00E-64 |
| WRIC_568 | GR302952 | 23 | 719 | photosystem II 10 kDa polypeptide (O. sativa) | 3.00E-46 |
| WRIS_1058 | GR302990 | 1 | 715 | Cytochrome b5（Mortierella alpina） | 6.00E-23 |
| WRIS_116 | GR303040 | 11 | 397 | putative hydroxypyruvate reductase (O. sativa) | 3.00E-26 |
| WRIS_1241 | GR303066 | 1 | 606 | glyceraldehyde-3-phosphate dehydrogenase（H. vulgare） | 1.00E-51 |
| WRIS_1300 | GR303092 | 1 | 366 | putative chlorophyll a/b-binding protein type III precursor (O. sativa) | 3.00E-20 |
| WRIS_1393 | GR303131 | 1 | 607 | glyoxysomal malate dehydrogenase (T. aestivum) | 2.00E-38 |
| WRIS_1408 | GR303135 | 1 | 648 | Oxidoreductase （Arabidopsis thaliana） | 1.00E-60 |
| WRIS_1453 | GR303149 | 1 | 677 | putative malate dehydrogenase (O. sativa) | 3.00E-82 |
| WRIS_146 | GR303158 | 1 | 579 | glyoxalase I (O. sativa) | 3.00E-15 |
| WRIS_1546 | GR303189 | 1 | 611 | NADPH thioredoxin reductase (O. sativa) | 2.00E-92 |
| WRIS_1586 | GR303202 | 1 | 592 | core chlorophyll a/b binding (CAB) protein ofphotosystem II (PSII) （H. vulgare） | 2.00E-87 |
| WRIS_160 | GR303213 | 1 | 678 | betaine aldehyde dehydrogenase-like (O. sativa) | 8.00E-90 |
| WRIS_161 | GR303217 | 1 | 497 | cytoplasmic malate dehydrogenase (O. sativa) | 9.00E-44 |
| WRIS_166 | GR303235 | 1 | 574 | mitochondrial NADH:ubiquinone oxidoreductase complex I（Gymnadeniaconopsea） | 5.00E-43 |
| WRIS_1748 | GR303265 | 1 | 487 | putative mitochondrial F0 ATP synthase D chain (O. sativa) | 1.00E-44 |
| WRIS_1752 | GR303268 | 1 | 321 | putative photosystem II reaction center W protein (O. sativa) | 2.00E-11 |
| WRIS_1869 | GR303324 | 1 | 713 | NAD dependent epimerase/dehydratase family protein (O. sativa) | 5.00E-70 |
| WRIS_190 | GR303341 | 1 | 701 | NAD kinase 2 (O. sativa) | 1.00E-66 |
| WRIS_1914 | GR303346 | 1 | 513 | putative long chain acyl-CoA synthetase (O. sativa) | 4.00E-39 |
| WRIS_1959 | GR303360 | 1 | 734 | Photosystem I reaction center subunit XI (O. sativa) | 1.00E -107 |
| WRIS_2040 | GR303388 | 1 | 246 | NdhC（H. vulgare） | 5.00E-23 |
| WRIS_2054 | GR303391 | 1 | 586 | Protochlorophyllide reductase B（Hordeum vulgare） | 4.00E-60 |
| WRIS_2079 | GR303403 | 1 | 495 | putative NADPH:quinone oxidoreductase (O. sativa) | 2.00E-30 |
| WRIS_2207 | GR303451 | 1 | 549 | ATP synthase gamma chain (O. sativa) | 2.00E-46 |
| WRIS_2226 | GR303461 | 1 | 607 | putative Aconitate hydratase (O. sativa) | 1.00E -103 |
| WRIS_2276 | GR303482 | 1 | 565 | ribulose-1,5-bisphosphate carboxylase/oxygenase large subunit（H. vulgare） | 0.00E+00 |
| WRIS_2292 | GR303486 | 1 | 644 | photosystem II 10 kDa polypeptide (O. sativa) | 1.00E-46 |
| WRIS_2377 | GR303527 | 1 | 468 | ATP-dependent Clp protease ATP-binding subunit clpACD4B (O. sativa) | 7.00E-13 |
| WRIS_2402 | GR303537 | 1 | 605 | NADH dehydrogenase subunit 5（Zea mays） | 0.00E+00 |
| WRIS_2414 | GR303545 | 1 | 488 | putative rubisco small subunit（Triticum turgidum subsp. durum） | 1.00E-36 |
| WRIS_2456 | GR303561 | 1 | 513 | putative mitochondrial Rieske protein（Sorghum bicolor） | 2.00E-32 |
| WRIS_2508 | GR303586 | 1 | 704 | putative acyl-CoA oxidase ACX3 (O. sativa) | 3.00E-82 |
| WRIS_2510 | GR303587 | 1 | 559 | A light-harvesting complex IIa protein | 7.00E-50 |
| WRIS_2518 | GR303590 | 1 | 555 | glyoxalase I (O. sativa) | 2.00E-22 |
| WRIS_2550 | GR303601 | 1 | 738 | putative pod-specific dehydrogenase SAC25 (O. sativa) | 5.00E-88 |
| WRIS_2602 | GR303619 | 1 | 713 | NADH:ubiquinone oxidoreductase-like（Solanum tuberosum） | 5.00E-78 |
| WRIS_2667 | GR303643 | 1 | 506 | Photosystem II reaction center Psb27 protein (O. sativa) | 4.00E-34 |
| WRIS_2671 | GR303646 | 1 | 751 | putative NADH dehydrogenase (O. sativa) | 1.00E -112 |
| WRIS_2686 | GR303652 | 1 | 733 | putative L-aspartate oxidase (O. sativa) | 2.00E-78 |
| WRIS_273 | GR303676 | 1 | 688 | putative photosystem II subunit (22KDa) precursor (O. sativa) | 1.00E-16 |
| WRIS_2783 | GR303697 | 1 | 773 | ribulose 1,5-bisphosphate carboxylase activase isoform 1（H. vulgare） | 1.00E -117 |
| WRIS_280 | GR303708 | 1 | 608 | putative photosystem II subunit (22KDa) precursor (O. sativa) | 7.00E-17 |
| WRIS_2859 | GR303723 | 1 | 523 | phosphoribulokinase; ribulose-5-phosphate kinase (T. aestivum) | 7.00E-37 |
| WRIS_3012 | GR303797 | 1 | 350 | photosystem 1 subunit 5 (T. aestivum) | 9.00E-11 |
| WRIS_3026 | GR303804 | 1 | 744 | ATP-citrate synthase, putative, expressed (O. sativa) | 6.00E-07 |
| WRIS_3038 | GR303809 | 1 | 669 | acetyl CoA synthetase（Deschampsia antarctica） | 2.00E-60 |
| WRIS_3084 | GR303829 | 1 | 642 | pentose-5-phosphate-3-epimerase （Solanum tuberosum） | 3.00E-87 |
| WRIS_3187 | GR303875 | 1 | 644 | pyridoxine 5'-phosphate oxidase, putative, expressed (O. sativa) | 1.00E-35 |
| WRIS_3188 | GR303876 | 1 | 732 | putative cytochrome b5 reductase (O. sativa) | 6.00E-69 |
| WRIS_3192 | GR303878 | 1 | 596 | succinate dehydrogenase (T. aestivum) | 5.00E-56 |
| WRIS_3196 | GR303880 | 1 | 280 | ribulose-1,5-bisphosphate carboxylase/oxygenase large subunit（Cortaderia selloana） | 1.00E-30 |
| WRIS_3245 | GR303895 | 1 | 678 | Oxygen-evolving enhancer protein 1, chloroplast precursor | 1.00E -105 |
| WRIS_3307 | GR303924 | 1 | 696 | 2-oxoglutarate/malate translocator, chloroplast precursor (O. sativa) | 1.00E -120 |
| WRIS_3335 | GR303932 | 1 | 699 | phosphoenolpyruvate carboxylase（H. vulgare） | 2.00E-75 |
| WRIS_3338 | GR303935 | 1 | 421 | putative 2-oxoglutarate/malate translocator (O. sativa) | 4.00E-29 |
| WRIS_3458 | GR303985 | 1 | 661 | putative dihydrolipoamide dehydrogenase precursor (O. sativa) | 5.00E-91 |
| WRIS_3544 | GR304019 | 1 | 663 | putative cytochrome b561 (O. sativa) | 4.00E-36 |
| WRIS_3572 | GR304032 | 1 | 544 | chlorophyll a/b binding protein 2 (O. sativa) | 9.00E-26 |
| WRIS_3695 | GR304078 | 1 | 558 | oxidoreductase,short chain dehydrogenase/reductase family protein (O. sativa) | 1.00E-36 |
| WRIS_3750 | GR304100 | 1 | 439 | NADPH-thioredoxin reductase (T. aestivum) | 3.00E-22 |
| WRIS_3977 | GR304161 | 1 | 254 | cytochrome P450 (O. sativa) | 3.00E-39 |
| WRIS_4082 | GR304190 | 1 | 524 | F1-ATPase（Pyrus pyrifolia） | 2.00E-29 |
| WRIS_4102 | GR304199 | 1 | 305 | NADH dehydrogenase 49KDa subunit （Acorus calamus） | 3.00E-23 |
| WRIS_4230 | GR304247 | 1 | 754 | putative 2-oxoglutarate/malate translocator (O. sativa) | 4.00E-84 |
| WRIS_4233 | GR304249 | 1 | 380 | Photosystem I reaction center subunit III | 2.00E-29 |
| WRIS_4251 | GR304254 | 1 | 260 | putative photosystem II reaction center W protein (O. sativa) | 2.00E-06 |
| WRIS_4266 | GR304260 | 1 | 546 | FAD dependent oxidoreductase family protein (O. sativa) | 5.00E-24 |
| WRIS_4367 | GR304299 | 1 | 394 | putative ATP synthase delta chain (O. sativa) | 6.00E-09 |
| WRIS_4389 | GR304308 | 1 | 405 | photosystem 1 subunit 5 (T. aestivum) | 6.00E-33 |
| WRIS_4411 | GR304318 | 1 | 554 | putative NAD synthetase (O. sativa) |  |
| WRIS_4421 | GR304322 | 1 | 384 | NADP-specific isocitrate dehydrogenase (O. sativa) | 4.00E-14 |
| WRIS_4482 | GR304343 | 1 | 624 | putative chlorophyll a/b-binding protein type III precursor (O. sativa) | 1.00E-74 |
| WRIS_4494 | GR304349 | 1 | 645 | photosystem II 10 kDa polypeptide (O. sativa) | 2.00E-46 |
| WRIS_4315 | GR304279 | 1 | 557 | utative dihydrolipoamide dehydrogenase precursor (O. sativa) | 3.00E-29 |
| WRIS_4400 | GR304315 | 1 | 451 | 4-hydroxyphenylpyruvate dioxygenase (T. aestivum) | 3.00E-31 |
| WRIS_4564 | GR304383 | 1 | 655 | ATP-dependent Clp protease proteolytic subunit (T. aestivum) | 1.00E -108 |
| WRIS_457 | GR304389 | 1 | 635 | oxidoreductase, 2OG-Fe oxygenase family protein (O. sativa) | 3.00E-18 |
| WRIS_4632 | GR304409 | 1 | 441 | NADPH-thioredoxin reductase (T. aestivum) | 1.00E-09 |
| WRIS_467 | GR304420 | 1 | 272 | AtpA（Elymus sibiricus） | 2.00E-17 |
| WRIS_4682 | GR304423 | 1 | 691 | ferredoxin-NADP(H) oxidoreductase (T. aestivum) | 1.00E-92 |
| WRIS_4723 | GR304440 | 1 | 626 | putative oxidoreductase （(O. sativa)） | 3.00E-99 |
| WRIS_4727 | GR304443 | 1 | 696 | ATP phosphoribosyltransferase family protein (O. sativa) | 3.00E-57 |
| WRIS_4756 | GR304458 | 1 | 552 | ATP-citrate synthase (O. sativa) | 3.00E-62 |
| WRIS_4778 | GR304465 | 1 | 540 | phosphoglycerate mutase (T. aestivum) | 1.00E -101 |
| WRIS_4840 | GR304498 | 1 | 604 | putative photosystem II protein reaction center W (O. sativa) | 3.00E-60 |
| WRIS_4844 | GR304500 | 1 | 491 | putative ATP synthase gamma chain 1 (O. sativa) | 2.00E-25 |
| WRIS_4872 | GR304521 | 1 | 681 | hemoglobin Hb2 (T. aestivum) | 0.00E+00 |
| WRIS_4912 | GR304543 | 1 | 668 | NADH-dependent glutamate synthase (O. sativa) | 1.00E -111 |
| WRIS_4953 | GR304561 | 1 | 631 | ribulose-1,5-bisphosphate carboxylase/oxygenase large subunit (T. aestivum) | 1.00E -118 |
| WRIS_4969 | GR304570 | 1 | 718 | chlorophyll a oxygenase (O. sativa) | 1.00E -112 |
| WRIS_4992 | GR304578 | 1 | 667 | putative phosphoribosylaminoimidazole carboxylase (O. sativa) | 3.00E-94 |
| WRIS_5007 | GR304585 | 1 | 618 | NADH dehydrogenase subunit 1 (T. aestivum) | 2.00E-79 |
| WRIS_5023 | GR304591 | 1 | 688 | putative cytochrome P450 (O. sativa) | 7.00E-57 |
| WRIS_5046 | GR304605 | 1 | 704 | putative cytochrome B5 (O. sativa) | 1.00E-63 |
| WRIS_5156 | GR304647 | 1 | 440 | plastocyanin precursor（H. vulgare） | 3.00E-48 |
| WRIS_5144 | GR304643 | 1 | 209 | PSII D1 protein (Prunus xyedoensis) | 1.00E-19 |
| WRIS_5208 | GR304673 | 1 | 549 | ferredoxin-NADP(H) oxidoreductase (T. aestivum) | 7.00E-53 |
| WRIS_5211 | GR304674 | 1 | 309 | hosphoenolpyruvate carboxylase（H. vulgare） | 1.00E-08 |
| WRIS_5231 | GR304679 | 1 | 373 | Cytochrome P450 family protein (O. sativa) | 2.00E-18 |
| WRIS_5260 | GR304692 | 1 | 685 | cytochrome P450-like (O. sativa) | 1.00E-74 |
| WRIS_528 | GR304700 | 1 | 753 | putative cytochrome c oxidase subunit (T. aestivum) | 2.00E-54 |
| WRIS_5329 | GR304714 | 1 | 615 | ATPase-like protein (O. sativa) | 5.00E-48 |
| WRIS_5339 | GR304717 | 1 | 756 | cytochrome f (T. aestivum) | 1.00E -103 |
| WRIS_5400 | GR304745 | 1 | 376 | Photosystem I reaction center subunit XI, chloroplast precursor (O. sativa) | 2.00E-32 |
| WRIS_5412 | GR304750 | 1 | 475 | cytochrome c oxidase subunit 1（Beta vulgaris subsp. vulgaris） | 2.00E-83 |
| WRIS_5491 | GR304779 | 1 | 402 | cytochrome f; gpetA (O. sativa) | 2.00E-27 |
| WRIS_5533 | GR304798 | 1 | 408 | Photosystem I reaction center subunit IV | 9.00E-24 |
| WRIS_5590 | GR304818 | 1 | 559 | phytoene synthase 2（Z. mays） | 7.00E-39 |
| WRIS_5689 | GR304859 | 1 | 654 | putative NADPH dehydrogenase (O. sativa) | 1.00E-63 |
| WRIS_5756 | GR304880 | 1 | 645 | putative cytochrome B5 (O. sativa) | 2.00E-64 |
| WRIS_5789 | GR304897 | 1 | 681 | ATP synthase alpha subunit (O. sativa) | 4.00E-42 |
| WRIS_5819 | GR304910 | 1 | 715 | putative mitochondrial NADH:ubiquinone oxidoreductase 29 kDasubunit (O. sativa) | 3.00E-77 |
| WRIS_5877 | GR304935 | 1 | 701 | FAD-dependent pyridine nucleotide-disulphide oxidoreductase;Calcium-binding EF-hand （Medicago truncatula） | 9.00E-53 |
| WRIS_5894 | GR304944 | 1 | 545 | NADP-specific isocitrate dehydrogenase (O. sativa) | 4.00E-49 |
| WRIS_5901 | GR304950 | 1 | 542 | LHCI-680, photosystem I antenna protein (Hordeum vulgare) | 5.00E-74 |
| WRIS_595 | GR304970 | 1 | 136 | Oxygen-evolving enhancer protein 2 (T. aestivum) | 1.00E-09 |
| WRIS_658 | GR305009 | 1 | 597 | putative NADH dehydrogenase (ubiquinone) chain PSST precursor (O. sativa) | 3.00E-81 |
| WRIS_768 | GR305038 | 1 | 444 | light-harvesting complex I（H. vulgare） | 6.00E-28 |
| WRIS_858 | GR305075 | 1 | 693 | Catalase-1 (T. aestivum) | 2.00E-22 |
| WRIS_891 | GR305086 | 1 | 463 | cytosolic glyceraldehyde-3-phosphate dehydrogenase (T. aestivum) | 2.00E-29 |
| WRIS_978 | GR305116 | 1 | 704 | Sedoheptulose-1,7-bisphosphatase (T. aestivum) | 3.00E-74 |
| WRIC_419 | GR302803 | 2 | 809 | oxidoreductase, 2OG-Fe oxygenase family protein | 5.00E-30 |
| WRIC_100 | GR302484 | 2 | 654 | chlorophyll a-b binding protein 3C-like (Solanum tuberosum) | 1.00E-92 |
| WRIC_385 | GR302769 | 2 | 469 | Chlorophyll a-b binding protein of LHCII type III （H. vulgare） | 2.00E-57 |
| WRIC_41 | GR302425 | 3 | 835 | Carbonic anhydrase | 3.00E-79 |
| WRIC_43 | GR302427 | 2 | 827 | plastid omega-3 fatty acid desaturase (T. aestivum) | 1.00E -130 |
| WRIC_63 | GR302447 | 3 | 718 | S-adenosylmethionine decarboxylase precursor（T. aestivum） | 7.00E-95 |
| WRIC_71 | GR302455 | 2 | 631 | glyoxysomal malate dehydrogenase (T. aestivum) | 2.00E-60 |
| WRIC_92 | GR302476 | 2 | 676 | Xylose isomerase（Hordeum vulgare） | 1.00E-54 |
| WRIC_96 | GR302480 | 2 | 714 | glyoxysomal malate dehydrogenase (T. aestivum) | 3.00E-68 |
| WRIC_119 | GR302503 | 2 | 962 | putative plastidic cysteine synthase 1 (O. sativa) | 3.00E-97 |
| WRIC_135 | GR302519 | 2 | 736 | putative glycine decarboxylase subunit (T. aestivum) | 3.00E-82 |
| WRIC_137 | GR302521 | 2 | 658 | S-adenosyl-L-homocysteine hydrolase（H. vulgare） | 5.00E-79 |
| WRIC_26 | GR302410 | 3 | 684 | Argininosuccinate lyase (O. sativa) | 1.00E-54 |
| WRIC_294 | GR302678 | 2 | 415 | putative carbamoyl phosphate synthetase small subunit (O. sativa) | 4.00E-25 |
| WRIC_311 | GR302695 | 4 | 743 | Glyceraldehyde-3-phosphate dehydrogenase（maize） | 1.00E -100 |
| WRIC_357 | GR302741 | 2 | 896 | putative gamma hydroxybutyrate dehydrogenase (O. sativa) | 1.00E -125 |
| WRIC_360 | GR302744 | 2 | 389 | putative aminotransferase (O. sativa) | 1.00E-08 |
| WRIC_434 | GR302818 | 2 | 742 | fructose-1,6-bisphosphatase （Pisum sativum） | 1.00E-77 |
| WRIC_451 | GR302835 | 3 | 1062 | inorganic pyrophosphatase (O. sativa) | 1.00E -113 |
| WRIC_456 | GR302840 | 5 | 946 | Phosphoglycerate kinase (T. aestivum) | 1.00E -125 |
| WRIC_537 | GR302921 | 2 | 897 | phosphoethanolamine methyltransferas (T. aestivum) | 6.00E-06 |
| WRIC_543 | GR302927 | 22 | 398 | putative glycolate oxidase (O. sativa) | 8.00E-17 |
| WRIC_566 | GR302950 | 2 | 439 | phosphate transporter 6（H. vulgare） | 1.00E-10 |
| WRIS_1181 | GR303045 | 1 | 570 | beta-cyanoalanine synthase (O. sativa) | 3.00E-47 |
| WRIS_1196 | GR303051 | 1 | 740 | triticain alpha (T. aestivum) | 1.00E-63 |
| WRIS_1211 | GR303056 | 1 | 634 | putative beta-alanine synthases (O. sativa) | 6.00E-94 |
| WRIS_1337 | GR303108 | 1 | 659 | putative aminopeptidase MAP1 precursor (O. sativa) | 1.00E -102 |
| WRIS_1368 | GR303117 | 1 | 340 | putative leucine aminopeptidase (O. sativa) | 1.00E-10 |
| WRIS_1535 | GR303182 | 1 | 634 | lipid binding (Arabidopsis thaliana) | 7.00E-10 |
| WRIS_1589 | GR303203 | 1 | 474 | putative aminopeptidase M (O. sativa) | 6.00E-18 |
| WRIS_1630 | GR303224 | 1 | 374 | putative beta-galactosidase (O. sativa) | 1.00E-45 |
| WRIS_1633 | GR303226 | 1 | 291 | putative acetylornithine aminotransferase (O. sativa) | 7.00E-27 |
| WRIS_174 | GR303266 | 1 | 494 | Cysteine synthase (O. sativa) | 2.00E-60 |
| WRIS_1778 | GR303279 | 1 | 400 | putative serine acetyltransferase (O. sativa) | 1.00E-08 |
| WRIS_1811 | GR303297 | 1 | 713 | beta-glucosidase isozyme 2 precursor (O. sativa) | 5.00E-59 |
| WRIS_1826 | GR303304 | 1 | 708 | reversibly glycosylated polypeptide (T. aestivum) | 1.00E -135 |
| WRIS_1859 | GR303318 | 1 | 581 | putative pyruvate kinase (Z. mays) | 7.00E-80 |
| WRIS_1995 | GR303371 | 1 | 723 | glycine decarboxylase P subunitx (Tritordeum sp.) | 2.00E-62 |
| WRIS_2025 | GR303385 | 1 | 751 | putative pyruvate dehydrogenase E1 beta subunit isoform 1 protein (O. sativa) | 1.00E -105 |
| WRIS_2071 | GR303402 | 1 | 478 | Putative 2,3-bisphosphoglycerate-independent phosphoglyceratemutase (O. sativa) | 2.00E-44 |
| WRIS_2080 | GR303404 | 1 | 602 | putative fructokinase (O. sativa) | 1.00E-53 |
| WRIS_2323 | GR303502 | 1 | 769 | phenylalanine ammonia-lyase（H. vulgare） | 0.00E+00 |
| WRIS_2389 | GR303530 | 1 | 485 | putative protein disulfide isomerase-related protein (O. sativa) | 9.00E-43 |
| WRIS_2420 | GR303547 | 1 | 566 | Lipolytic enzyme, G-D-S-L (Medicago truncatula) | 1.00E-56 |
| WRIS_242 | GR303551 | 1 | 654 | mannose-6-phosphate isomerase, class I family protein (O. sativa) | 3.00E-80 |
| WRIS_2465 | GR303567 | 1 | 567 | putative gamma-glutamyl hydrolase (O. sativa) | 8.00E-54 |
| WRIS_2494 | GR303578 | 1 | 517 | pyruvate,orthophosphate dikinase (maize) | 1.00E-38 |
| WRIS_2554 | GR303603 | 1 | 761 | sucrose synthase (Lolium perenne) | 1.00E -112 |
| WRIS_262 | GR303627 | 1 | 511 | putative chloroplast cysteine synthase 1 precursor (Nicotianatabacum) | 1.00E-91 |
| WRIS_2729 | GR303672 | 1 | 754 | putative tripeptidyl peptidase II (O. sativa) | 1.00E -110 |
| WRIS_2773 | GR303693 | 1 | 761 | rhamnose biosynthetic enzyme 1, putative, expressed (O. sativa) | 7.00E-88 |
| WRIS_2798 | GR303704 | 1 | 625 | putative UDP-glucose glucosyltransferase1 (O. sativa) | 4.00E-67 |
| WRIS_2835 | GR303715 | 1 | 697 | carboxylic ester hydrolase/ catalytic/ hydrolase （Arabidopsisthaliana） | 2.00E-91 |
| WRIS_2909 | GR303749 | 1 | 558 | prolyl 4-hydroxylase, putative (O. sativa) | 5.00E-57 |
| WRIS_2912 | GR303751 | 1 | 456 | putative anthranilate phosphoribosyltransferase (O. sativa) | 2.00E-71 |
| WRIS_2956 | GR303768 | 1 | 427 | putative Xaa-Pro aminopeptidase 2 (O. sativa) | 8.00E-28 |
| WRIS_2982 | GR303781 | 1 | 646 | plastid omega-3 fatty acid desaturase (T. aestivum) | 1.00E -128 |
| WRIS_3045 | GR303812 | 1 | 564 | arginase （Prunus armeniaca） | 2.00E-55 |
| WRIS_3048 | GR303813 | 1 | 247 | Fructose-bisphospha aldolase (O. sativa) | 5.00E-07 |
| WRIS_3121 | GR303850 | 1 | 692 | carboxylesterase-like (O. sativa) | 4.00E-48 |
| WRIS_3185 | GR303873 | 11 | 684 | Aldose 1-epimerase family protein (O. sativa) | 7.00E-98 |
| WRIS_3283 | GR303910 | 1 | 740 | lipase (class 3)-like protein (O. sativa) |  |
| WRIS_3285 | GR303912 | 1 | 741 | putative glutaryl-CoA dehydrogenase (O. sativa) | 1.00E -112 |
| WRIS_3341 | GR303937 | 1 | 720 | hydroxymethylbutenyl 4-diphosphate synthase （Z. mays） | 1.00E -117 |
| WRIS_3390 | GR303956 | 1 | 680 | Enolase (O. sativa) | 1.00E -113 |
| WRIS_3419 | GR303968 | 1 | 389 | lingual lipase-like (O. sativa) | 1.00E-22 |
| WRIS_3428 | GR303971 | 1 | 635 | Caffeic acid 3-O-methyltransferase | 3.00E-84 |
| WRIS_3478 | GR303993 | 1 | 341 | putative GDP-mannose pyrophosphorylase (O. sativa) | 4.00E-32 |
| WRIS_3619 | GR304049 | 1 | 680 | putative CBS domain containing protein (O. sativa) | 4.00E-31 |
| WRIS_3626 | GR304052 | 1 | 657 | putative carbamoyl phosphate synthetase (O. sativa) | 1.00E-46 |
| WRIS_3711 | GR304085 | 1 | 404 | cyanase (O. sativa) | 1.00E-25 |
| WRIS_3712 | GR304086 | 1 | 695 | putative uridine kinase/uracil phosphoribosyltransferase (O. sativa) | 1.00E -108 |
| WRIS_3729 | GR304091 | 1 | 641 | dolichyl-diphosphooligosaccharide-protein glycotransferase/oligosaccharyl transferase （Arabidopsis thaliana） | 2.00E-54 |
| WRIS_3874 | GR304138 | 1 | 691 | putative glycine decarboxylase subunit （T. aestivum） |  |
| WRIS_3845 | GR304125 | 1 | 466 | UDP-glucose dehydrogenase （Cinnamomum osmophloeum） | 4.00E-54 |
| WRIS_3989 | GR304164 | 1 | 640 | glucosamine-fructose-6-phosphate aminotransferase (O. sativa) | 6.00E-98 |
| WRIS_2553 | GR303602 | 1 | 644 | putative GDSL-like lipase/acylhydrolase（O. sativa） | 5.00E-22 |
| WRIS_3 | GR304166 | 1 | 697 | Serine hydroxymethyltransferase, mitochondrial precursor (O. sativa) | 2.00E-78 |
| WRIS_4009 | GR304170 | 1 | 275 | Inosine triphosphate pyrophosphatase (O. sativa) | 2.00E-11 |
| WRIS_4076 | GR304188 | 1 | 697 | putative phosphatidylserine synthase (O. sativa) | 1.00E-59 |
| WRIS_4089 | GR304193 | 1 | 302 | branched-chain amino acid aminotransferase -like (O. sativa) | 2.00E-08 |
| WRIS_4086 | GR304192 | 1 | 517 | thiosulfate sulfurtransferase (O. sativa) | 1.00E-41 |
| WRIS_4107 | GR304202 | 1 | 271 | sucrose:sucrose 1-fructosytransferase (T. aestivum) | 3.00E-37 |
| WRIS_4122 | GR304210 | 1 | 602 | GCN5-related N-acetyltransferase (GNAT) family protein-like (O. sativa) | 1.00E-57 |
| WRIS_4253 | GR304256 | 1 | 296 | putative Omega-6 fatty acid desaturase (O. sativa) | 2.00E-46 |
| WRIS_4292 | GR304269 | 1 | 520 | fructose 1,6-bisphosphate aldolase precursor（Avena sativa） | 5.00E-76 |
| WRIS_4310 | GR304276 | 1 | 622 | Fructose-1,6-bisphosphatase（T. aestivum） | 1.00E -101 |
| WRIS_4415 | GR304320 | 1 | 665 | inorganic pyrophosphatase (O. sativa) | 0.00E+00 |
| WRIS_4628 | GR304407 | 1 | 647 | aldehyde dehydrogenase, putative (O. sativa) | 1.00E-76 |
| WRIS_4699 | GR304428 | 1 | 405 | putative serine decarboxylase (O. sativa) | 3.00E-14 |
| WRIS_4730 | GR304446 | 1 | 443 | Carbonic anhydrase, chloroplast precursor |  |
| WRIS_4804 | GR304482 | 1 | 658 | O-methyltransferase (O. sativa) | 1.00E-69 |
| WRIS_4824 | GR304492 | 1 | 558 | 'putative 6-phosphofructo-2-kinase (O. sativa) | 5.00E-51 |
| WRIS_4842 | GR304499 | 1 | 559 | putative beta-galactosidase (O. sativa) | 8.00E-51 |
| WRIS_4858 | GR304510 | 1 | 518 | esterase/lipase/thioesterase-like protein (O. sativa) | 2.00E-40 |
| WRIS_4884 | GR304524 | 1 | 630 | Histidinol dehydrogenase, chloroplast precursor (O. sativa) | 2.00E-59 |
| WRIS_4946 | GR304558 | 1 | 569 | glutamine synthetase（Saccharum officinarum） | 9.00E-69 |
| WRIS_4966 | GR304568 | 1 | 561 | tryptophan decarboxylase（H. vulgare） | 9.00E-95 |
| WRIS_4988 | GR304576 | 1 | 718 | putative aspartate aminotransferase (O. sativa) | 3.00E-72 |
| WRIS_5015 | GR304588 | 1 | 646 | arabinoxylan arabinofuranohydrolase isoenzyme AXAH-II（H. vulgare） | 1.00E -124 |
| WRIS_5093 | GR304621 | 1 | 670 | glucose-6-phosphate isomerase (T. aestivum) | 1.00E -114 |
| WRIS_5130 | GR304640 | 1 | 697 | sucrase-like protein (O. sativa) | 1.00E-11 |
| WRIS_5213 | GR304675 | 1 | 625 | putative serine decarboxylase (O. sativa) | 1.00E -112 |
| WRIS_5292 | GR304702 | 1 | 691 | methionine synthase 1 enzyme（H. vulgare） | 1.00E -125 |
| WRIS_5308 | GR304709 | 1 | 609 | ribulose bisphosphate carboxylase large chain （Anthoxanthumodoratum） | 4.00E-44 |
| WRIS_5347 | GR304719 | 11 | 337 | GDP-D-mannose pyrophosphorylase （Viola baoshanensis） | 5.00E-06 |
| WRIS_5411 | GR304749 | 1 | 698 | putative hosphatidylinositol/phophatidylcholine transfer protein (O. sativa) | 2.00E-60 |
| WRIS_5439 | GR304759 | 1 | 538 | GCN5-related N-acetyltransferase-like (O. sativa) | 5.00E-27 |
| WRIS_5485 | GR304776 | 1 | 740 | UDP-D-glucuronate decarboxylase（H. vulgare） | 1.00E -103 |
| WRIS_5505 | GR304785 | 1 | 679 | phosphoglycerate mutase family, putative (O. sativa) | 1.00E-91 |
| WRIS_5515 | GR304789 | 1 | 671 | putative carboxymethylenebutenolidase (O. sativa) | 1.00E-68 |
| WRIS_5616 | GR304829 | 1 | 399 | Triosephosphate isomerase | 3.00E-25 |
| WRIS_5650 | GR304845 | 1 | 509 | nucleotide pyrophosphatase/phosphodiesterase （H. vulgare） | 2.00E-58 |
| WRIS_5735 | GR304872 | 1 | 459 | phenylalanine ammonia-lyase | 1.00E-20 |
| WRIS_5866 | GR304930 | 1 | 634 | reversibly glycosylated polypeptide (T. aestivum) | 6.00E-46 |
| WRIS_5874 | GR304932 | 1 | 551 | peptidase-like protein (O. sativa) | 4.00E-24 |
| WRIS_5895 | GR304945 | 1 | 356 | xyloglucan endo-transglycosylase homolog | 2.00E-14 |
| WRIS_5905 | GR304953 | 1 | 271 | GAD1（H. vulgare） | 9.00E-40 |
| WRIS_5983 | GR304981 | 1 | 541 | 6-phosphogluconate dehydrogenase isoenzyme B （Z. mays） | 3.00E-33 |
| WRIS_674 | GR305013 | 1 | 583 | putative esterase (O. sativa) | 4.00E-30 |
| WRIS_802 | GR305051 | 1 | 527 | Lactoylglutathione lyase (Methylglyoxalase) | 5.00E-13 |
| WRIS_866 | GR305081 | 1 | 402 | putative GDSL-like lipase/acylhydrolase (O. sativa) | 5.00E-14 |
| WRIS_2817 | GR303710 | 1 | 380 | plastid glutamine synthetase isoform GS2c (T. aestivum) | 4.00E-14 |
| **Signal transduction** | | | | | |
| WRIC_24 | GR302408 | 2 | 495 | casein protein kinase 2 alpha subunit （Lolium perenne） | 3.00E-19 |
| WRIC_22 | GR302406 | 2 | 554 | putative phospholipase (O. sativa) | 2.00E-13 |
| WRIC_131 | GR302515 | 2 | 909 | chloroplast SRP receptor cpFtsY precursor（Z. mays） | 1.00E -149 |
| WRIC_146 | GR302530 | 2 | 888 | Serine/threonine-protein phosphatase PP1 (O. sativa) | 1.00E-36 |
| WRIC_152 | GR302536 | 2 | 719 | Saposin -like type B (O. sativa) | 4.00E-50 |
| WRIC_233 | GR302617 | 2 | 782 | catalytic/ protein phosphatase type 2C （Arabidopsis thaliana） | 9.00E-97 |
| WRIC_271 | GR302655 | 2 | 793 | WD40-like Beta Propeller Repeat family protein (O. sativa) | 4.00E-89 |
| WRIC_384 | GR302768 | 3 | 1475 | 'putative 6-phosphofructo-2-kinase (O. sativa) | 0.00E+00 |
| WRIC_482 | GR302866 | 3 | 755 | Thiol peroxidase that functions as a hydroperoxide receptor tosense intracellular hydroperoxide levels and transduce aredox signal to the Yap1p transcription factor（Saccharomyces cerevisiae） | 2.00E-53 |
| WRIC_536 | GR302920 | 2 | 780 | GTP-binding protein SAR1A (O. sativa) | 8.00E-75 |
| WRIC_559 | GR302943 | 2 | 976 | putative GTP-binding protein typA (O. sativa) | 1.00E -120 |
| WRIC_571 | GR302955 | 2 | 581 | CIPK-like protein 1 (O. sativa) | 2.00E-26 |
| WRIC_579 | GR302963 | 2 | 818 | Calcineurin B-like protein 3 (O. sativa) | 3.00E-82 |
| WRIS_1079 | GR303004 | 1 | 677 | putative fructokinase (O. sativa) | 1.00E -107 |
| WRIS_1145 | GR303030 | 1 | 351 | putative BRI1-KD interacting protein 112 (O. sativa) | 3.00E-20 |
| WRIS_1189 | GR303048 | 1 | 621 | 1-phosphatidylinositol-3-phosphate 5-kinase-like (O. sativa) | 1.00E-85 |
| WRIS_1232 | GR303062 | 1 | 598 | chitin elicitor binding protein (O. sativa) | 4.00E-20 |
| WRIS_1238 | GR303065 | 1 | 563 | Gamma-aminobutyric acid receptor-associated protein-like 2 (O. sativa) | 3.00E-39 |
| WRIS_1331 | GR303104 | 1 | 669 | putative rab1 small GTP-binding protein (O. sativa) | 6.00E-85 |
| WRIS_1362 | GR303115 | 1 | 531 | putative TGF-beta receptor interacting protein (O. sativa) | 1.00E-51 |
| WRIS_1437 | GR303144 | 1 | 571 | Contains similarity to a receptor-like serine/threonine kinase（Arabidopsis thaliana） | 7.00E-82 |
| WRIS_1460 | GR303154 | 1 | 340 | putative non-cell-autonomous protein pathway2; plasmodesmalreceptor (O. sativa) | 6.00E-18 |
| WRIS_1501 | GR303168 | 1 | 465 | Profilin-1 （Hordeum vulgare） | 1.00E-17 |
| WRIS_1625 | GR303220 | 1 | 557 | putative MAP3K delta-1 protein kinase (O. sativa) | 6.00E-81 |
| WRIS_1665 | GR303234 | 1 | 665 | small GTP-binding protein domain containing protein (O. sativa) | 3.00E-40 |
| WRIS_1782 | GR303283 | 1 | 698 | root hair defective 3 GTP-binding protein (T. aestivum) | 1.00E -115 |
| WRIS_1819 | GR303299 | 1 | 522 | GUN4-like family protein (O. sativa) | 4.00E-39 |
| WRIS_1835 | GR303309 | 1 | 486 | C2 domain-containing protein-like Copines (O. sativa) | 2.00E-18 |
| WRIS_1927 | GR303350 | 1 | 730 | small Ras-related GTP-binding protein (T. aestivum) | 1.00E -113 |
| WRIS_1946 | GR303356 | 1 | 630 | putative ADP-ribosylation factor (O. sativa) | 3.00E-46 |
| WRIS_1987 | GR303367 | 1 | 650 | CAAX peptidase（H. vulgare） | 1.00E-83 |
| WRIS_2061 | GR303396 | 1 | 651 | ras-related GTP binding protein (O. sativa) | 7.00E-91 |
| WRIS_2111 | GR303416 | 1 | 366 | Ras-related protein RHN1, putative (O. sativa) | 1.00E-21 |
| WRIS_2341 | GR303509 | 1 | 727 | proline-rich family protein (O. sativa) | 5.00E-85 |
| WRIS_2363 | GR303521 | 1 | 688 | hydrolase/ protein serine/threonine phosphatase （Arabidopsisthaliana） | 7.00E-68 |
| WRIS_2397 | GR303534 | 1 | 497 | GTP-binding protein, putative, expressed (O. sativa) | 1.00E-28 |
| WRIS_2398 | GR303535 | 1 | 700 | Sedoheptulose-1,7-bisphosphatase (T. aestivum) | 1.00E -125 |
| WRIS_2435 | GR303553 | 1 | 703 | putative serine/threonine protein phosphatase PP1 (O. sativa) | 2.00E-81 |
| WRIS_2485 | GR303572 | 1 | 732 | putative protein kinase Xa21 (O. sativa) | 3.00E-44 |
| WRIS_263 | GR303631 | 1 | 638 | putative serine-threonine kinase receptor-associated protein (O. sativa) | 7.00E-34 |
| WRIS_2745 | GR303680 | 1 | 716 | putative receptor serine/threonine kinase PR5K (O. sativa) | 1.00E-34 |
| WRIS_2747 | GR303682 | 1 | 707 | putative heme A farnesyltransferase homolog (O. sativa) | 4.00E-47 |
| WRIS_2876 | GR303731 | 1 | 335 | putative receptor-mediated endocytosis 1 isoform I (O. sativa) | 2.00E-14 |
| WRIS_2969 | GR303775 | 1 | 113 | putative serine/threonine-protein kinase ctr1 (O. sativa) | 1.00E-11 |
| WRIS_3182 | GR303872 | 1 | 437 | similar to protein kinase AtSIK (O. sativa) | 2.00E-08 |
| WRIS_3194 | GR303879 | 1 | 395 | FHA domain containing protein (O. sativa) | 9.00E-19 |
| WRIS_3284 | GR303911 | 1 | 714 | putative serine/threonine protein kinase (O. sativa) | 6.00E-84 |
| WRIS_3412 | GR303964 | 1 | 769 | putative DsPTP1 protein (O. sativa) | 2.00E-40 |
| WRIS_3423 | GR303969 | 1 | 683 | putative ATP/GTP-binding protein (O. sativa) | 1.00E-52 |
| WRIS_3451 | GR303982 | 1 | 676 | protein phosphatase 2A regulatory A subunit （Lolium perenne） | 1.00E-07 |
| WRIS_3461 | GR303987 | 1 | 592 | Leucine Rich Repeat family protein (O. sativa) | 4.00E-17 |
| WRIS_3468 | GR303989 | 1 | 602 | ATP binding / protein kinase/ protein serine/threonine kinase/protein-tyrosine kinase (Arabidopsis thaliana) | 2.00E-47 |
| WRIS_3517 | GR304006 | 1 | 692 | putative protein kinase (O. sativa) | 1.00E-130 |
| WRIS_3613 | GR304045 | 1 | 579 | putative serine-threonine protein kinase (O. sativa) | 3.00E-48 |
| WRIS_3641 | GR304059 | 1 | 581 | putative calcium binding protein (O. sativa) | 4.00E-57 |
| WRIS_372 | GR304092 | 1 | 765 | SacIy domain containing protein, expressed (O. sativa) | 9.00E-73 |
| WRIS_3759 | GR304102 | 1 | 444 | myosin heavy chain-like (O. sativa) | 6.00E-26 |
| WRIS_3774 | GR304105 | 1 | 701 | protein kinase family protein (O. sativa) | 1.00E-71 |
| WRIS_3783 | GR304107 | 1 | 709 | putative ATP/GTP-binding protein (O. sativa) | 1.00E -110 |
| WRIS_3971 | GR304159 | 1 | 507 | OsIre1p (O. sativa) | 1.00E-46 |
| WRIS_4091 | GR304194 | 1 | 546 | serine/threonine-protein kinase (O. sativa) | 1.00E-21 |
| WRIS_4121 | GR304209 | 1 | 408 | signal peptidase I family protein (O. sativa) | 2.00E-19 |
| WRIS_4152 | GR304222 | 1 | 684 | Ras-related protein RIC1 (O. sativa) | 2.00E-98 |
| WRIS_4246 | GR304253 | 1 | 330 | ADP-ribosylation factor (atharanthus roseus) | 4.00E-32 |
| WRIS_4287 | GR304267 | 1 | 622 | Putative vacuolar sorting receptor protein homolog (O. sativa) | 1.00E-41 |
| WRIS_4297 | GR304271 | 1 | 601 | Signal recognition particle 54 kDa protein 1（H. vulgare） | 3.00E-75 |
| WRIS_4325 | GR304284 | 1 | 618 | putative Septum-promoting GTP-binding protein 1 (O. sativa) | 1.00E-12 |
| WRIS_4383 | GR304305 | 1 | 426 | pyruvate kinase-like (Deschampsia Antarctica) | 4.00E-19 |
| WRIS_431 | GR304281 | 1 | 723 | protein kinase family protein (O. sativa) | 2.00E-88 |
| WRIS_4555 | GR304377 | 1 | 643 | anther ethylene-upregulated protein ER1 (O. sativa) | 2.00E-41 |
| WRIS_4619 | GR304405 | 1 | 694 | calcium-dependent protein kinase CPK1 adapter protein 2-like (O. sativa) | 4.00E-82 |
| WRIS_4648 | GR304416 | 1 | 565 | putative protein kinase (O. sativa) | 5.00E-92 |
| WRIS_4691 | GR304426 | 1 | 621 | putative Serine/threonine Kinase (O. sativa) | 5.00E-45 |
| WRIS_4705 | GR304430 | 1 | 493 | Mitogen-activated protein kinase 2 (MAP kinase 2) (O. sativa) | 2.00E-27 |
| WRIS_4816 | GR304487 | 1 | 386 | Cop1 protein-like (O. sativa) | 5.00E-59 |
| WRIS_4856 | GR304508 | 1 | 587 | putative signal transduction protein（H. vulgare） | 2.00E-43 |
| WRIS_4885 | GR304525 | 1 | 603 | putative protein kinase Xa21 (O. sativa) | 1.00E-22 |
| WRIS_4908 | GR304540 | 1 | 568 | Phosphoglycerate kinase (T. aestivum) | 2.00E-36 |
| WRIS_490 | GR304541 | 1 | 496 | putative ethylene-induced calmodulin-binding protein (O. sativa) | 5.00E-16 |
| WRIS_5193 | GR304667 | 1 | 265 | Structure Of Signal Recognition Particle Receptor (O. sativa) | 2.00E-06 |
| WRIS_524 | GR304687 | 1 | 590 | thyroid hormone receptor-associated protein complex component (O. sativa) | 5.00E-28 |
| WRIS_5391 | GR304739 | 1 | 759 | Protein kinase domain containing protein (O. sativa) | 7.00E-70 |
| WRIS_5418 | GR304752 | 1 | 736 | putative Mitogen-activated protein kinase(MAPK) (O. sativa) | 1.00E-68 |
| WRIS_5448 | GR304762 | 1 | 522 | root hair defective 3 GTP-binding protein (T. aestivum) | 6.00E-21 |
| WRIS_5464 | GR304767 | 1 | 660 | Ras-related protein Rab-6A (O. sativa) | 6.00E-92 |
| WRIS_5546 | GR304802 | 1 | 477 | pyruvate,orthophosphate dikinase | 1.00E-29 |
| WRIS_5600 | GR304823 | 1 | 675 | putative small GTP-binding protein Ran (O. sativa) | 2.00E-77 |
| WRIS_5674 | GR304854 | 1 | 619 | putative adenosine kinase (O. sativa) | 2.00E-22 |
| WRIS_5757 | GR304881 | 1 | 661 | putative signal recognition particle receptor (O. sativa) | 6.00E-72 |
| WRIS_5766 | GR304884 | 1 | 568 | putative receptor kinase (O. sativa) | 7.00E-77 |
| WRIS_583 | GR304918 | 1 | 327 | putative GTP-binding protein Rab7a（O. sativa） | 4.00E-14 |
| WRIS_5933 | GR304959 | 1 | 311 | Protein kinase domain containing protein (O. sativa) | 1.00E-14 |
| WRIS_5962 | GR304972 | 1 | 616 | Protein kinase domain containing protein (O. sativa) | 2.00E-41 |
| WRIS_5963 | GR304973 | 1 | 693 | protein kinase C substrate 80K-H isoform 2 -like (O. sativa) | 5.00E-78 |
| WRIS_702 | GR305022 | 1 | 569 | GTP1/OBG family protein (O. sativa) | 4.00E-61 |
| WRIS_808 | GR305055 | 1 | 681 | Armadillo/beta-catenin-like repeat family protein (O. sativa) | 1.00E-94 |
| WRIS_823 | GR305059 | 1 | 369 | serine/threonine protein kinase (Axonopus compressu) | 1.00E-06 |
| WRIS_82 | GR305063 | 1 | 248 | small GTP-binding protein domain containing protein (O. sativa) | 2.00E-16 |
| WRIS_853 | GR305073 | 1 | 698 | phophatdylinositol 4-kinase (O. sativa) | 9.00E-32 |
| WRIS_85 | GR305077 | 1 | 195 | protein phosphatase 2C (O. sativa) | 1.00E-21 |
| WRIS_867 | GR305082 | 1 | 676 | putative mitogen activated protein kinase (MAP K) (O. sativa) | 3.00E-39 |
| WRIS_952 | GR305106 | 1 | 722 | putative protein phosphatase type-2C | 1.00E -110 |
| WRIS_999 | GR305126 | 1 | 367 | TAK33 (T. aestivum) | 4.00E-16 |
| **Protein destination and storage** | | | | | |
| WRIC_17 | GR302401 | 2 | 907 | aspartic proteinase (T. aestivum) | 1.00E -161 |
| WRIC_51 | GR302435 | 2 | 155 | ubiquitin-conjugating enzyme （Hyacinthus orientalis） | 2.00E-16 |
| WRIC_105 | GR302489 | 2 | 647 | chopper chaperone（H. vulgare） | 3.00E-31 |
| WRIC_209 | GR302593 | 2 | 806 | putative actin depolymerizing factor (O. sativa) | 1.00E-48 |
| WRIC_213 | GR302597 | 3 | 670 | Ubiquitin（Medicago truncatula） | 8.00E-35 |
| WRIC_307 | GR302691 | 3 | 807 | immature spike ubiquitin-conjugating enzyme 2 (T. aestivum) | 2.00E-83 |
| WRIC_346 | GR302730 | 3 | 801 | polyubiquitin 2（Deschampsia antarctica） | 2.00E-69 |
| WRIC_400 | GR302784 | 2 | 925 | putative proteasome subunit alpha type 3 (O. sativa) | 1.00E -105 |
| WRIC_403 | GR302787 | 2 | 638 | cysteine proteinase（H. vulgare） | 8.00E-20 |
| WRIC_404 | GR302788 | 6 | 718 | immature spike ubiquitin-conjugating enzyme 2（T. aestivum） | 1.00E-83 |
| WRIC_428 | GR302812 | 3 | 952 | Polyubiquitin（Sporobolus stapfianus） | 1.00E-78 |
| WRIC_489 | GR302873 | 3 | 487 | putative copper chaperone (O. sativa) | 3.00E-31 |
| WRIC_512 | GR302896 | 2 | 502 | ubiquitin-like protein (O. sativa) | 4.00E-21 |
| WRIC_513 | GR302897 | 2 | 717 | ADP-ribosylation factor (O. sativa) | 3.00E-70 |
| WRIC_522 | GR302906 | 2 | 697 | polyubiquitin （Sporobolus stapfianus） | 2.00E-59 |
| WRIC_533 | GR302917 | 3 | 853 | Polyubiquitin（Z.mays） | 5.00E-27 |
| WRIC_558 | GR302942 | 2 | 507 | cysteine protease (T. aestivum) | 9.00E-31 |
| WRIC_563 | GR302947 | 2 | 427 | ubiquitin-conjugating enzyme (E2) （Nicotiana tabacum） | 1.00E-14 |
| WRIC_569 | GR302953 | 2 | 852 | Polyubiquitin（Sporobolus stapfianus） | 1.00E-78 |
| WRIC_573 | GR302957 | 2 | 672 | Thiol protease aleurain precursor（H. vulgare） | 9.00E-55 |
| WRIS_1060 | GR302992 | 1 | 575 | putative alpha-mannosidase (O. sativa) | 2.00E-31 |
| WRIS_1119 | GR303019 | 1 | 635 | Ubiquitin-conjugating enzyme E2-23 kDa (O. sativa) | 8.00E-76 |
| WRIS_1170 | GR303041 | 1 | 556 | cysteine-type endopeptidase/ ubiquitin thiolesterase （Arabidopsist haliana） | 6.00E-52 |
| WRIS_1373 | GR303120 | 1 | 613 | F-box protein family-like (O. sativa) | 1.00E-69 |
| WRIS_1474 | GR303160 | 1 | 320 | putative prolyl endopeptidase(Arabidopsis thaliana) | 2.00E-11 |
| WRIS_1572 | GR303197 | 1 | 572 | TPR repeat (Medicago truncatula) | 2.00E-15 |
| WRIS_1600 | GR303208 | 1 | 623 | putative protease (O. sativa) | 5.00E-83 |
| WRIS_1694 | GR303242 | 1 | 395 | beta1 proteasome-1D (Aegilops tauschii) | 1.00E-38 |
| WRIS_1704 | GR303248 | 1 | 227 | Ubiquitin carboxyl-terminal hydrolase family protein (O. sativa) | 3.00E-25 |
| WRIS_1884 | GR303331 | 1 | 634 | peptidase-like protein (O. sativa) | 2.00E-42 |
| WRIS_1891 | GR303334 | 11 | 505 | ubiquitin/ribosomal fusion protein (T. aestivum) | 2.00E-21 |
| WRIS_1887 | GR303332 | 1 | 719 | TPR repeat (Medicago truncatula) | 2.00E-73 |
| WRIS_1998 | GR303373 | 1 | 384 | immature spike ubiquitin-conjugating enzyme 2 (T. aestivum) | 6.00E-36 |
| WRIS_2005 | GR303375 | 1 | 331 | 26S proteasome regulatory particle non-ATPase subunit10 (O. sativa) | 2.00E-10 |
| WRIS_2018 | GR303380 | 1 | 384 | Ubiquitin-activating enzyme E1 2 (T. aestivum) | 2.00E-28 |
| WRIS_2139 | GR303430 | 1 | 775 | Tetratricopeptide repeat protein 11 (O. sativa) | 1.00E-60 |
| WRIS_2390 | GR303531 | 1 | 742 | Proteasome subunit alpha type 5 (O. sativa) | 1.00E -108 |
| WRIS_2467 | GR303568 | 1 | 706 | putative carboxyl-terminal proteinase (O. sativa) | 1.00E-74 |
| WRIS_252 | GR303596 | 1 | 331 | beta1 proteasome-7D (Aegilops tauschii) | 1.00E-17 |
| WRIS_2689 | GR303654 | 1 | 701 | tetratricopeptide repeat-containing protein-like (O. sativa) | 2.00E-71 |
| WRIS_2892 | GR303740 | 1 | 573 | Proteasome subunit beta type 3 (O. sativa) | 2.00E-63 |
| WRIS_2920 | GR303754 | 1 | 551 | Proteasome subunit alpha type 5 (O. sativa) | 6.00E-85 |
| WRIS_2943 | GR303763 | 1 | 500 | Ubiquitin （Medicago truncatula） | 2.00E-30 |
| WRIS_3053 | GR303817 | 1 | 560 | ubiquitin（Hevea brasiliensis） | 5.00E-61 |
| WRIS_3123 | GR303851 | 1 | 434 | alpha 2 subunit of 20S proteasome (O. sativa) | 8.00E-33 |
| WRIS_20 | GR303412 | 1 | 389 | putative peptidyl-prolyl cis-trans isomerase NIMA-interacting (O. sativa) | 3.00E-31 |
| WRIS_3279 | GR303908 | 1 | 678 | putative Ubiquitin carrier protein UBC7 (O. sativa) | 4.00E-77 |
| WRIS_3314 | GR303925 | 1 | 479 | multifunctional protein (O. sativa) | 6.00E-25 |
| WRIS_3324 | GR303929 | 1 | 623 | armadillo/beta-catenin repeat family protein (O. sativa) | 2.00E-52 |
| WRIS_3530 | GR304016 | 1 | 603 | putative protease (O. sativa) | 7.00E-57 |
| WRIS_3707 | GR304084 | 1 | 542 | ubiquitin-conjugating enzyme (E2) （Nicotiana tabacum） | 4.00E-40 |
| WRIS_4060 | GR304182 | 1 | 486 | reversibly glycosylated polypeptide (T. aestivum) | 4.00E-21 |
| WRIS_4180 | GR304233 | 1 | 549 | polyubiquitin 10 （Arachis hypogaea） | 8.00E-32 |
| WRIS_4394 | GR304311 | 1 | 445 | Ubiquitin-conjugating enzyme E2-23 kDa（Oryza sativa） | 1.00E-75 |
| WRIS_4362 | GR304298 | 1 | 429 | E3 ubiquitin protein ligase UPL1 (O. sativa) | 1.00E-57 |
| WRIS_4487 | GR304346 | 1 | 667 | tetratricopeptide repeat domain 5-like protein (O. sativa) | 8.00E-53 |
| WRIS_4563 | GR304382 | 1 | 556 | ubiquitin-conjugating enzyme OsUBC5b (O. sativa) | 1.00E-56 |
| WRIS_4640 | GR304412 | 1 | 452 | maize protease inhibitor（Tripsacum dactyloides） | 2.00E-15 |
| WRIS_4850 | GR304503 | 1 | 475 | 26S proteasome ATPase subunit （Lupinus albus） | 1.00E-37 |
| WRIS_4865 | GR304516 | 1 | 344 | AAA-protein subdomain（Medicago truncatula） | 2.00E-10 |
| WRIS_4888 | GR304527 | 1 | 701 | 20S proteasome beta 4 subunit (T. aestivum) | 8.00E-83 |
| WRIS_4891 | GR304528 | 1 | 689 | POZ domain protein family-like (O. sativa) | 8.00E-54 |
| WRIS_4897 | GR304531 | 1 | 682 | TPR Domain containing protein (O. sativa) | 2.00E-71 |
| WRIS_4919 | GR304547 | 1 | 637 | peptidyl-prolyl cis-trans isomerase (O. sativa) | 2.00E-65 |
| WRIS_4998 | GR304579 | 1 | 691 | TPR repeat region family protein (O. sativa) | 4.00E-12 |
| WRIS_4 | GR304581 | 1 | 602 | putative protease inhibitor（H. vulgare） | 7.00E-36 |
| WRIS_5286 | GR304699 | 1 | 338 | ATP-dependent Clp protease ATP-binding subunit clpACD4B (O. sativa) | 2.00E-10 |
| WRIS_52 | GR304704 | 1 | 124 | putative immunophilin / FKBP-type peptidyl-prolyl cis-transisomerase (O. sativa) | 8.00E-10 |
| WRIS_5486 | GR304777 | 1 | 725 | polyubiquitin 2 （Deschampsia antarctica） | 4.00E-20 |
| WRIS_5796 | GR304901 | 1 | 616 | 26S proteasome ATPase subunit（Lupinus albus） | 1.00E-63 |
| WRIS_863 | GR305078 | 1 | 669 | putative UBA3 (O. sativa) | 1.00E-103 |
| **Protein synthesis** | | | | | |
| WRIC_45 | GR302429 | 2 | 652 | putative ribosomal S1 protein (O. sativa) | 4.00E-43 |
| WRIC_56 | GR302440 | 3 | 624 | putative protein translation factor Sui1 (O. sativa) | 7.00E-56 |
| WRIC_66 | GR302450 | 4 | 990 | ribosomal protein L32 (T. aestivum) | 2.00E-17 |
| WRIC_115 | GR302499 | 3 | 1475 | Chloroplast 50S ribosomal protein L2（H. vulgare） | 1.00E-141 |
| WRIC_160 | GR302544 | 2 | 587 | ribosomal protein S8 (T. aestivum) | 1.00E-70 |
| WRIC_180 | GR302564 | 2 | 371 | putative ribosomal protein (O. sativa) | 9.00E-22 |
| WRIC_192 | GR302576 | 13 | 1432 | ribosomal protein S7（Z. mays） | 3.00E-80 |
| WRIC_239 | GR302623 | 10 | 665 | putative ribosomal protein L26 (O. sativa) | 6.00E-39 |
| WRIC_269 | GR302653 | 2 | 727 | 60S acidic ribosomal protein P0 (O. sativa) | 1.00E-83 |
| WRIC_281 | GR302665 | 2 | 1074 | ribosomal protein L16 (O. sativa) | 3.00E-70 |
| WRIC_299 | GR302683 | 2 | 705 | putative ribosomal protein L28 (O. sativa) | 2.00E-28 |
| WRIC_300 | GR302684 | 2 | 802 | putative ribosomal protein S12 (O. sativa) | 5.00E-64 |
| WRIC_323 | GR302707 | 2 | 1074 | ribosomal protein L3 (T. aestivum) | 0.00E+00 |
| WRIC_405 | GR302789 | 2 | 767 | Elongation factor 1-gamma (O. sativa) | 4.00E-77 |
| WRIC_410 | GR302794 | 4 | 1010 | translation elongation factor 1 alpha-subunit | 1.00E-148 |
| WRIC_442 | GR302826 | 3 | 896 | ribosomal protein L33 (T. aestivum) | 9.00E-30 |
| WRIC_443 | GR302827 | 5 | 941 | translation initiation factor 5A (O. sativa) | 2.00E-78 |
| WRIC_449 | GR302833 | 3 | 797 | translation initiation factor (T. aestivum) | 2.00E-50 |
| WRIC_450 | GR302834 | 2 | 765 | putative ribosomal protein (O. sativa) | 3.00E-71 |
| WRIC_467 | GR302851 | 4 | 697 | translation initiation factor (T. aestivum) | 1.00E-50 |
| WRIC_477 | GR302861 | 2 | 616 | Mg-chelatase subunit XANTHA-F（H. vulgare） | 8.00E-72 |
| WRIC_546 | GR302930 | 2 | 1008 | Elongation factor 1-alpha (EF-1-alpha) (T. aestivum) | 1.00E-132 |
| WRIS_106 | GR302998 | 1 | 491 | 40S ribosomal protein S16 (O. sativa) | 9.00E-41 |
| WRIS_1132 | GR303025 | 1 | 522 | translation elongation factor-1 alpha （Pseudotsuga menziesii var.menziesii） | 5.00E-25 |
| WRIS_1162 | GR303037 | 1 | 596 | ribosomal protein L36 (T. aestivum) | 5.00E-53 |
| WRIS_1345 | GR303110 | 1 | 612 | 50S ribosomal protein L6 (O. sativa) | 5.00E-90 |
| WRIS_1410 | GR303137 | 1 | 406 | chloroplast polyprotein of elongation factor Ts precursor（Arabidopsis thaliana） | 1.00E-13 |
| WRIS_1420 | GR303141 | 1 | 665 | putative 50S ribosomal protein L21 (O. sativa) | 1.00E-43 |
| WRIS_1440 | GR303145 | 1 | 233 | translation initiation factor SUI1 （Medicago truncatula） | 2.00E-12 |
| WRIS_1475 | GR303161 | 1 | 519 | elongation factor (T. aestivum) | 2.00E-50 |
| WRIS_1476 | GR303162 | 1 | 682 | glutathione synthetase (T. aestivum) | 3.00E-41 |
| WRIS_1539 | GR303184 | 1 | 624 | putative 60S ribosomal protein L28（O.sativa） | 1.00E-66 |
| WRIS_168 | GR303240 | 1 | 583 | ribosomal protein L30 (T. aestivum) | 7.00E-51 |
| WRIS_1736 | GR303260 | 1 | 419 | putative chloroplast ribosomal protein L1 (O. sativa) | 1.00E-10 |
| WRIS_1791 | GR303289 | 1 | 392 | Ribosomal protein S30（Medicago truncatula） | 1.00E-08 |
| WRIS_1895 | GR303337 | 1 | 622 | ribosomal Pr 117 (T. aestivum) | 8.00E-73 |
| WRIS_1904 | GR303339 | 1 | 386 | rps3 (T. aestivum) | 1.00E-53 |
| WRIS_2092 | GR303410 | 1 | 428 | Chloroplast 30S ribosomal protein S10 (O. sativa) | 1.00E-43 |
| WRIS_2141 | GR303432 | 1 | 539 | 40S ribosomal protein S9 (O. sativa) | 0.00E+00 |
| WRIS_2212 | GR303454 | 1 | 452 | putative ribosomal protein S29 (O. sativa) | 9.00E-29 |
| WRIS_236 | GR303523 | 1 | 585 | putative phenylalanyl-tRNA synthetase beta chain (O. sativa) | 6.00E-28 |
| WRIS_2450 | GR303559 | 1 | 618 | 60S ribosomal protein L17-2（H. vulgare） | 4.00E-53 |
| WRIS_2498 | GR303581 | 1 | 740 | Eukaryotic translation initiation factor 2 subunit beta (T. aestivum) | 1.00E-109 |
| WRIS_2564 | GR303608 | 1 | 306 | putative 60S ribosomal protein L39 (O. sativa) | 0.00E+00 |
| WRIS_2567 | GR303610 | 1 | 651 | 40S ribosomal protein S14 (O. sativa) | 7.00E-62 |
| WRIS_2802 | GR303706 | 1 | 374 | putative 60S ribosomal protein L10A (O. sativa) | 7.00E-62 |
| WRIS_2828 | GR303713 | 1 | 631 | Eukaryotic initiation factor 4A (O. sativa) | 6.00E-95 |
| WRIS_2870 | GR303727 | 1 | 719 | putative 40S ribosomal protein S8 (O. sativa) | 0.00E+00 |
| WRIS_2958 | GR303770 | 1 | 345 | ribosomal protein （Bromus inermis） | 1.00E-59 |
| WRIS_2990 | GR303787 | 1 | 348 | ribosomal protein S1（Sorghum bicolor） | 1.00E-53 |
| WRIS_3024 | GR303802 | 1 | 329 | 40S subunit ribosomal protein (O. sativa) | 1.00E-25 |
| WRIS_3104 | GR303839 | 1 | 407 | putative 40S ribosomal protein 25S (O. sativa) | 1.00E-33 |
| WRIS_3186 | GR303874 | 1 | 794 | 60S ribosomal protein L10-2 (Putative tumor suppressor SG12) (O. sativa) | 1.00E-110 |
| WRIS_3269 | GR303905 | 1 | 549 | putative lysyl-tRNA synthetase (O. sativa) | 1.00E-23 |
| WRIS_3340 | GR303936 | 1 | 189 | ribosomal protein L36; rpl36 (O. sativa) | 2.00E-14 |
| WRIS_3646 | GR304062 | 1 | 571 | putative ribosomal protein L10a (O. sativa) | 0.00E+00 |
| WRIS_3661 | GR304068 | 1 | 469 | ribosomal protein （Bromus inermis） | 7.00E-27 |
| WRIS_3824 | GR304117 | 1 | 604 | acidic ribosomal protein P2 (T. aestivum) | 5.00E-28 |
| WRIS_385 | GR304131 | 1 | 207 | putative elongation factor （Arabidopsis thaliana） | 2.00E-15 |
| WRIS_3925 | GR304150 | 1 | 403 | elongation factor（T. aestivum） | 0.00E+00 |
| WRIS_3985 | GR304163 | 1 | 648 | putative translational inhibitor protein (O. sativa) | 5.00E-54 |
| WRIS_4098 | GR304197 | 1 | 455 | ribosomal protein L36 (T. aestivum) | 3.00E-23 |
| WRIS_4260 | GR304258 | 1 | 714 | 40S ribosomal protein S23 (O. sativa) | 9.00E-77 |
| WRIS_4424 | GR304323 | 1 | 519 | 40S ribosomal protein S23 (O. sativa) | 3.00E-46 |
| WRIS_4484 | GR304344 | 1 | 498 | Elongation factor 1-alpha (EF-1-alpha) (T. aestivum) | 9.00E-28 |
| WRIS_4554 | GR304376 | 1 | 619 | 60S ribosomal protein L17-1（H. vulgare） | 5.00E-79 |
| WRIS_463 | GR304411 | 1 | 147 | rps 19（Secale cereale） | 6.00E-15 |
| WRIS_4674 | GR304418 | 1 | 624 | ribosomal protein L11 | 6.00E-84 |
| WRIS_4788 | GR304473 | 1 | 621 | putative ribosomal protein S7 from chromosome 10 chloroplastinsetion (O. sativa) | 0.00E+00 |
| WRIS_4826 | GR304494 | 1 | 300 | 60S ribosomal protein L7A（Arabidopsis thaliana） | 8.00E-09 |
| WRIS_4864 | GR304515 | 1 | 600 | putative elongation factor 1 beta（Hordeum vulgare） | 7.00E-49 |
| WRIS_5029 | GR304595 | 1 | 431 | putative ribosomal protein S29 (O. sativa) | 2.00E-24 |
| WRIS_5393 | GR304741 | 1 | 802 | putative translation initiation factor (O. sativa) | 0.00E+00 |
| WRIS_547 | GR304773 | 1 | 645 | 50S ribosomal protein L6 (O. sativa) | 4.00E-97 |
| WRIS_5520 | GR304792 | 1 | 442 | ribosomal protein S3 (T. aestivum) | 4.00E-44 |
| WRIS_5550 | GR304805 | 1 | 657 | translation initiation factor IF-3-like (O. sativa) | 2.00E-41 |
| WRIS_5725 | GR304867 | 11 | 604 | ribosomal protein L14 (T. aestivum) | 1.00E-45 |
| WRIS_5734 | GR304871 | 1 | 757 | initiation factor (iso)4f p82 subunit | 1.00E-108 |
| WRIS_5767 | GR304885 | 1 | 600 | translational elongation factor Tu (O. sativa) | 2.00E-65 |
| WRIS_5876 | GR304934 | 1 | 510 | 40S subunit ribosomal protein (O. sativa) | 4.00E-52 |
| WRIS_607 | GR304991 | 1 | 327 | cytoplasmic ribosomal protein L18 (O. sativa) | 2.00E-23 |
| WRIS_612 | GR304995 | 1 | 620 | elongation factor (T. aestivum) | 1.00E-67 |
| WRIS_698 | GR305020 | 1 | 579 | putative eukaryotic translation initiation factor (O. sativa) | 1.00E-88 |
| WRIS_957 | GR305108 | 1 | 679 | 40S ribosomal protein S27（H. vulgare） | 7.00E-38 |
| WRIC_298 | GR302682 | 2 | 1107 | eukaryotic translation initiation factor 5（Z. mays） | 1.00E-101 |
| WRIC_350 | GR302734 | 3 | 718 | eukaryotic translation initiation factor 5A1 (T. aestivum) | 5.00E-89 |
| **Transporters** | | | | | |
| WRIC_16 | GR302400 | 2 | 626 | putative ABC transporter (O. sativa) | 4.00E-75 |
| WRIC_37 | GR302421 | 2 | 805 | putative transmembrane protein(TOM3) (O. sativa) | 3.00E-29 |
| WRIC_94 | GR302478 | 2 | 633 | putative H(+)-transporting ATP synthase (O. sativa) | 1.00E-30 |
| WRIC_141 | GR302525 | 2 | 765 | MtN3-like (O. sativa) | 1.00E-84 |
| WRIC_215 | GR302599 | 2 | 418 | H+-transporting ATP synthase chain 9-like protein (O. sativa) | 5.00E-16 |
| WRIC_216 | GR302600 | 5 | 1087 | Plasma membrane ATPase (Proton pump) (T. aestivum) | 1.00E-106 |
| WRIC_388 | GR302772 | 2 | 630 | ATP synthase subunit C family protein (O. sativa) | 1.00E-13 |
| WRIC_392 | GR302776 | 4 | 1639 | putative glycine hydroxymethyltransferase (O. sativa) | 1.00E-23 |
| WRIC_431 | GR302815 | 5 | 799 | MtN3-like (O. sativa) | 6.00E-86 |
| WRIC_476 | GR302860 | 2 | 639 | Protein transport protein SEC61 subunit gamma (O. sativa) | 2.00E-20 |
| WRIC_484 | GR302868 | 2 | 535 | transport protein subunit-like （Arabidopsis thaliana） | 2.00E-10 |
| WRIC_523 | GR302907 | 2 | 1150 | transferase, transferring glycosyl groups（Arabidopsis thaliana） | 1.00E-114 |
| WRIC_560 | GR302944 | 2 | 675 | Aquaporin（Hordeum vulgare） | 2.00E-56 |
| WRIS_1056 | GR302988 | 1 | 608 | Plasma membrane ATPase (T. aestivum) | 2.00E-98 |
| WRIS_1326 | GR303102 | 1 | 556 | Tryptophan/tyrosine permease family protein (O. sativa) | 2.00E-48 |
| WRIS_1380 | GR303124 | 1 | 650 | putative glucose-6-phosphate/phosphate- translocator precursor (O. sativa) | 2.00E-38 |
| WRIS_1383 | GR303126 | 1 | 663 | putative nicotinate phosphoribosyltransferase | 1.00E-86 |
| WRIS_1620 | GR303218 | 1 | 350 | sucrose transporter 2（Z. mays） | 1.00E-11 |
| WRIS_1635 | GR303227 | 1 | 360 | methyltransferase-like (O. sativa) | 4.00E-10 |
| WRIS_181 | GR303300 | 1 | 263 | H+-transporting two-sector ATPase | 1.00E-40 |
| WRIS_1719 | GR303253 | 1 | 598 | synaptobrevin-like protein (O. sativa) | 5.00E-33 |
| WRIS_1776 | GR303277 | 1 | 647 | GDP-Mannose transporter （Arabidopsis thaliana） | 3.00E-24 |
| WRIS_1798 | GR303292 | 1 | 691 | ABC-type transport system-like (O. sativa) | 8.00E-49 |
| WRIS_1912 | GR303344 | 1 | 556 | putative system A transporter isoform 2 (O. sativa) | 2.00E-08 |
| WRIS_1961 | GR303361 | 1 | 701 | triose phosphate translocator (T. aestivum) | 1.00E-103 |
| WRIS_200 | GR303377 | 1 | 630 | glycolipid transfer protein-like (O. sativa) | 6.00E-47 |
| WRIS_2062 | GR303397 | 1 | 715 | iron transport protein 1 (O. sativa) | 4.00E-82 |
| WRIS_2065 | GR303399 | 1 | 349 | putative membrane protein (O. sativa) | 2.00E-09 |
| WRIS_2088 | GR303408 | 1 | 717 | putative CLB1 protein (O. sativa) | 1.00E-36 |
| WRIS_2147 | GR303434 | 1 | 418 | 'unknown protein, contains zip zinc transporter protein (O. sativa) | 2.00E-33 |
| WRIS_2176 | GR303440 | 1 | 721 | putative amino acid transport protein（O. sativa） | 1.00E-104 |
| WRIS_2225 | GR303460 | 1 | 669 | Translocase of chloroplast 34, putative (O. sativa) | 5.00E-61 |
| WRIS_2238 | GR303466 | 1 | 600 | hexose transporter（Z. mays） | 4.00E-70 |
| WRIS_2218 | GR303457 | 1 | 188 | lipid transporter（Arabidopsis thaliana） | 5.00E-07 |
| WRIS_2246 | GR303469 | 1 | 523 | MRP-like ABC transporter (O. sativa) | 8.00E-20 |
| WRIS_2408 | GR303540 | 1 | 711 | Eukaryotic porin family protein (O. sativa) | 2.00E-87 |
| WRIS_2421 | GR303548 | 1 | 534 | putative monosaccharide transporter 3 (O. sativa) | 9.00E-33 |
| WRIS_2447 | GR303557 | 1 | 686 | Phosphatidylinositol transfer protein (O. sativa) | 1.00E-114 |
| WRIS_2463 | GR303565 | 1 | 405 | Vacuolar cation/proton exchanger 1b (Ca(2+)/H(+) exchanger 1b) (O. sativa) | 4.00E-13 |
| WRIS_2734 | GR303675 | 1 | 747 | Transmembrane protein G5p (O. sativa) | 6.00E-69 |
| WRIS_2834 | GR303714 | 1 | 580 | plasma membrane intrinsic protein 1 (T. aestivum) | 1.00E-25 |
| WRIS_2854 | GR303720 | 1 | 393 | ABC transporter family protein (O. sativa) | 2.00E-47 |
| WRIS_3262 | GR303901 | 1 | 395 | aquaporin（H. vulgare） | 3.00E-40 |
| WRIS_3436 | GR303976 | 1 | 506 | putative transmembrane protein (O. sativa) | 3.00E-25 |
| WRIS_3527 | GR304015 | 1 | 623 | Metal transporter Nramp2 (O. sativa) | 7.00E-16 |
| WRIS_3628 | GR304053 | 1 | 611 | electron carrier/ electron transporter/ iron ion binding（Arabidopsis thaliana） | 1.00E-37 |
| WRIS_3674 | GR304072 | 1 | 597 | ABC1 family protein-like (O. sativa) | 4.00E-38 |
| WRIS_4058 | GR304181 | 1 | 569 | putative potential copper-transporting ATPase (O. sativa) | 8.00E-62 |
| WRIS_4073 | GR304187 | 1 | 376 | amino acid permease I, putative (O. sativa) | 6.00E-33 |
| WRIS_4105 | GR304201 | 1 | 556 | membrane related protein (O. sativa) | 9.00E-79 |
| WRIS_429 | GR304272 | 1 | 361 | putative Na+/Ca2+ antiporter （Arabidopsis thaliana） | 4.00E-09 |
| WRIS_4391 | GR304309 | 1 | 669 | putative VAMP-associated protein （Arabidopsis thaliana） | 3.00E-43 |
| WRIS_4501 | GR304353 | 1 | 471 | putative Ca2+/H+-exchanging protein (O. sativa) | 6.00E-50 |
| WRIS_4502 | GR304354 | 1 | 576 | putative selenium binding protein (O. sativa) | 1.00E-100 |
| WRIS_4860 | GR304513 | 1 | 290 | putative non-transporter ABC protein AbcF1 (O. sativa) | 7.00E-06 |
| WRIS_4933 | GR304554 | 1 | 505 | putative amino acid transporter (O. sativa) | 1.00E-33 |
| WRIS_5110 | GR304630 | 1 | 506 | PIP aquaporin isoform（H. vulgare） | 1.00E-24 |
| WRIS_5313 | GR304710 | 1 | 683 | Tryptophan/tyrosine permease family protein (O. sativa) | 4.00E-42 |
| WRIS_5431 | GR304756 | 1 | 457 | ammonium transporter (T. aestivum) | 6.00E-07 |
| WRIS_5575 | GR304812 | 1 | 280 | plasma membrane H+-ATPase (T. aestivum) | 3.00E-31 |
| WRIS_5596 | GR304821 | 1 | 592 | putative peroxisomal Ca-dependent solute carrier（Oryza sativa） | 2.00E-15 |
| WRIS_5598 | GR304822 | 1 | 414 | HvPIP1;5（H. vulgare） | 9.00E-22 |
| WRIS_561 | GR304832 | 1 | 589 | putative integral membrane protein (O. sativa) | 6.00E-29 |
| WRIS_5676 | GR304855 | 1 | 621 | major facilitator superfamily protein (O. sativa) | 3.00E-26 |
| WRIS_5852 | GR304924 | 1 | 742 | histidine amino acid transporter (O. sativa) | 1.00E-97 |
| WRIS_5900 | GR304949 | 1 | 704 | ABC transporter family protein (O. sativa) | 9.00E-10 |
| WRIS_5955 | GR304968 | 1 | 470 | putative peroxisomal membrane protein (O. sativa) | 7.00E-82 |
| WRIS_618 | GR304997 | 1 | 192 | membrane protein （Saccharum hybrid cultivar H65-7052） | 3.00E-24 |
| WRIS_3452 | GR303983 | 1 | 467 | 37 kDa inner envelope membrane protein (O. sativa) | 8.00E-39 |
| **Transcriptions** | | | | | |
| WRIC_128 | GR302512 | 3 | 649 | cap-binding protein CBP20 (O. sativa) | 1.00E-35 |
| WRIC_168 | GR302552 | 2 | 744 | zinc finger protein-like (O. sativa) | 7.00E-57 |
| WRIC_217 | GR302601 | 10 | 872 | rRNA intron-encoded homing endonuclease (O. sativa) | 2.00E-20 |
| WRIC_266 | GR302650 | 2 | 907 | putative transcription initiation factor (O. sativa) | 8.00E-48 |
| WRIC_272 | GR302656 | 2 | 585 | putative RNA-binding protein cp33 (O. sativa) | 3.00E-28 |
| WRIC_330 | GR302714 | 3 | 1027 | RNA binding protein, putative, expressed (O. sativa) | 1.00E-170 |
| WRIC_470 | GR302854 | 2 | 374 | Sig5（O. sativa） | 1.00E-14 |
| WRIS_1144 | GR303029 | 1 | 724 | Histone-like transcription factor and archaeal histone familyprotein, expressed (O. sativa) | 6.00E-86 |
| WRIS_1193 | GR303050 | 1 | 487 | PCD/DCoH-like protein 2-like (O. sativa) | 3.00E-26 |
| WRIS_1246 | GR303068 | 1 | 564 | splicing factor 4-like protein (O. sativa) | 2.00E-51 |
| WRIS_1252 | GR303071 | 1 | 739 | ZIM motif family protein, expressed (O. sativa) | 9.00E-25 |
| WRIS_1328 | GR303103 | 1 | 154 | DNA-directed RNA polymerase beta'' chain | 1.00E-08 |
| WRIS_1400 | GR303132 | 1 | 731 | DHHC zinc finger domain containing protein | 1.00E-102 |
| WRIS_1745 | GR303263 | 1 | 508 | Ps16 protein (T. aestivum) | 1.00E-21 |
| WRIS_1915 | GR303347 | 1 | 483 | mRNA capping enzyme, C-terminal domain containing protein (O. sativa) | 1.00E-23 |
| WRIS_2303 | GR303494 | 1 | 664 | Associated with HOX family protein, expressed (O. sativa) | 3.00E-30 |
| WRIS_2331 | GR303506 | 1 | 778 | cp31BHv (Hordeum vulgare) | 3.00E-99 |
| WRIS_2664 | GR303641 | 1 | 345 | poly(A)-binding protein (T. aestivum) | 3.00E-23 |
| WRIS_2720 | GR303668 | 1 | 646 | putative RNA helicase RH22 (O. sativa) | 1.00E-79 |
| WRIS_2827 | GR303712 | 1 | 434 | putative RAE1 (RNA export 1, S.pombe) homolog (O. sativa) | 4.00E-14 |
| WRIS_304 | GR303814 | 1 | 680 | RNA recognition motif (RRM)-containing protein-like (O. sativa) | 1.00E-39 |
| WRIS_324 | GR303898 | 1 | 490 | bHLH transcription factor PTF1(Z. mays) | 2.00E-17 |
| WRIS_337 | GR303949 | 1 | 692 | putative S-like RNase (O. sativa) | 2.00E-87 |
| WRIS_3523 | GR304011 | 1 | 730 | putative regulator of nonsense transcripts 1 homolog | 4.00E-20 |
| WRIS_3650 | GR304063 | 1 | 334 | putative HLA-B associated transcript 1 (O. sativa) | 5.00E-15 |
| WRIS_3832 | GR304120 | 1 | 620 | BTH-induced ERF transcriptional factor 1 (O. sativa) | 1.00E-50 |
| WRIS_4314 | GR304278 | 1 | 442 | putative apoptosis antagonizing transcription factor (O. sativa) | 2.00E-13 |
| WRIS_4348 | GR304293 | 1 | 669 | putative RNA-binding protein (O. sativa) | 2.00E-40 |
| WRIS_4495 | GR304350 | 1 | 688 | putative leucine zipper protein (O. sativa) | 1.00E-61 |
| WRIS_4574 | GR304387 | 1 | 365 | putative poly(A) binding protein II (O. sativa) | 4.00E-46 |
| WRIS_4786 | GR304472 | 1 | 640 | Leucine Rich Repeat family protein (O. sativa) | 9.00E-06 |
| WRIS_4820 | GR304489 | 1 | 653 | putative transfactor (O. sativa) | 3.00E-05 |
| WRIS_4825 | GR304493 | 1 | 651 | RNA polymerase I specific transcription initiation factor (O. sativa) | 2.00E-25 |
| WRIS_4859 | GR304511 | 1 | 746 | putative bHLH protein (O. sativa) | 1.00E-40 |
| WRIS_489 | GR304534 | 1 | 558 | putative SBP-domain protein (O. sativa) | 3.00E-46 |
| WRIS_4898 | GR304532 | 1 | 295 | putative histone H2B (O. sativa) | 3.00E-10 |
| WRIS_4904 | GR304537 | 1 | 701 | transcriptional co-repressor -like (O. sativa) | 4.00E-72 |
| WRIS_5236 | GR304681 | 1 | 721 | leucine zipper protein zip1 (T. aestivum) | 1.00E-70 |
| WRIS_5376 | GR304733 | 1 | 661 | putative RNA helicase (O. sativa) | 1.00E-104 |
| WRIS_5405 | GR304746 | 1 | 607 | putative MYB transcription factor (O. sativa) | 2.00E-17 |
| WRIS_5643 | GR304841 | 1 | 477 | splicing factor-like (O. sativa) | 2.00E-27 |
| WRIS_5943 | GR304964 | 1 | 433 | putative RNA Binding Protein (O. sativa) | 5.00E-06 |
| WRIS_5949 | GR304966 | 1 | 384 | nuclease I（H. vulgare） | 4.00E-13 |
| WRIS_621 | GR304999 | 1 | 462 | putative DNA-directed RNA polymerase II subunit (O. sativa) | 2.00E-45 |
| WRIS_91 | GR305096 | 1 | 352 | putative ATP-dependent RNA helicase (O. sativa) | 1.00E-28 |
| WRIS_920 | GR305097 | 1 | 725 | guanine nucleotide-binding protein beta subujit-like protein (O. sativa) | 6.00E-07 |
| WRIS_707 | GR305024 | 1 | 574 | zinc finger (C3HC4-type RING finger) protein family -like (O. sativa) | 3.00E-13 |
| WRIS_60 | GR304994 | 1 | 121 | putative zinc finger protein ZmZf (Z. mays) | 1.00E-08 |
| WRIS_5795 | GR304900 | 1 | 366 | DNA binding zinc finger protein-like (O. sativa) | 7.00E-26 |
| WRIS_5658 | GR304849 | 1 | 407 | putative zinc finger protein 216 (O. sativa) | 7.00E-24 |
| WRIS_4334 | GR304288 | 1 | 474 | putative zinc-finger protein (O. sativa) | 9.00E-08 |
| WRIS_3663 | GR304069 | 1 | 668 | CCCH-type zinc finger protein-like protein (O. sativa) | 1.00E-42 |
| WRIS_3363 | GR303945 | 1 | 586 | zinc finger POZ domain protein-like (O. sativa) | 4.00E-34 |
| WRIS_2922 | GR303756 | 1 | 686 | putative zinc finger DNA-binding protein (O. sativa) | 2.00E-36 |
| WRIS_2539 | GR303598 | 1 | 461 | zinc finger protein family-like (O. sativa) | 2.00E-27 |
| WRIS_2396 | GR303533 | 1 | 470 | putative DHHC-type zinc finger domain-containing protein (O. sativa) | 4.00E-14 |
| WRIC_508 | GR302892 | 1 | 882 | zinc finger protein-like (O. sativa) | 4.00E-72 |
| WRIS_4234 | GR304250 | 1 | 458 | CCCH-type zinc finger protein-like protein | 2.00E-27 |
| WRIS_4735 | GR304450 | 1 | 632 | putative SWIM protein; putative Zn-finger protein (T. aestivum) | 2.00E-10 |
| WRIS_1228 | GR303059 | 1 | 466 | nucleic acid binding (Arabidopsis thaliana) | 0.00E+00 |
| **Cell structure** | | | | | |
| WRIC_3 | GR302387 | 2 | 714 | chloroplast inner envelope protein (O. sativa) | 5.00E-51 |
| WRIC_95 | GR302479 | 2 | 621 | putative actin depolymerizing factor (O. sativa) | 6.00E-36 |
| WRIC_142 | GR302526 | 17 | 927 | ndhD gene product (Z. mays) | 6.00E-49 |
| WRIC_310 | GR302694 | 3 | 480 | putative FtsH-like protein Pftf precursor (O. sativa) | 4.00E-41 |
| WRIC_313 | GR302697 | 2 | 721 | putative p60 katanin (O. sativa) | 4.00E-48 |
| WRIC_471 | GR302855 | 5 | 1116 | Actin (Musa x paradisiaca) | 1.00E-178 |
| WRIC_565 | GR302949 | 3 | 1070 | putative cellulose synthase-like protein OsCslE1 (O. sativa) | 1.00E-124 |
| WRIS_1008 | GR302973 | 1 | 731 | tubulin-specific chaperone C-like protein (O. sativa) | 2.00E-65 |
| WRIS_1116 | GR303017 | 1 | 568 | IFA-binding protein-like (O. sativa) | 8.00E-28 |
| WRIS_1464 | GR303156 | 1 | 602 | putative ankyrin repeat family protein (O. sativa) | 1.00E-17 |
| WRIS_1759 | GR303269 | 1 | 666 | fiber protein Fb19 (O. sativa) | 5.00E-29 |
| WRIS_1743 | GR303262 | 1 | 579 | Fibronectin type III domain containing protein (O. sativa) | 5.00E-52 |
| WRIS_2237 | GR303465 | 1 | 674 | Cytochrome b6-f complex iron-sulfur subunit (T. aestivum) | 1.00E-95 |
| WRIS_2312 | GR303497 | 1 | 565 | putative actin-depolymerizing factor 1 (O. sativa) | 7.00E-24 |
| WRIS_2548 | GR303600 | 1 | 692 | thylakoid lumen protein (O. sativa) | 3.00E-61 |
| WRIS_2762 | GR303690 | 1 | 496 | putative cryptochrome dash (O. sativa) | 1.00E-64 |
| WRIS_3439 | GR303977 | 1 | 662 | putative microtubule bundling polypeptide TMBP200 (O. sativa) | 1.00E-54 |
| WRIS_3558 | GR304026 | 1 | 679 | protoporphyrin IX Mg-chelatase subunit precursor | 1.00E-119 |
| WRIS_3609 | GR304044 | 1 | 503 | putative suppressor of actin 1 (O. sativa) | 5.00E-40 |
| WRIS_3888 | GR304142 | 1 | 610 | Actin (T. aestivum) | 2.00E-110 |
| WRIS_3930 | GR304152 | 1 | 357 | Actin（Setaria italica） | 1.00E-11 |
| WRIS_4360 | GR304297 | 1 | 785 | actin（H. vulgare） | 2.00E-77 |
| WRIS_5083 | GR304617 | 1 | 700 | fibrillin-like protein (O. sativa) | 2.00E-78 |
| WRIS_5087 | GR304618 | 1 | 533 | putative fiber protein Fb14 (O. sativa) | 7.00E-33 |
| WRIS_5433 | GR304757 | 1 | 458 | Profilin-1（Hordeum vulgare） | 4.00E-11 |
| WRIS_5553 | GR304806 | 1 | 684 | cellulose synthase BoCesA1b（Bambusa oldhamii） | 1.00E-28 |
| WRIS_967 | GR305113 | 1 | 502 | putative small nuclear ribonucleoprotein U1A (O. sativa) | 8.00E-35 |
| **Cell growth/division** | | | | | |
|
| WRIC_123 | GR302507 | 2 | 627 | reticulon（H. vulgare） | 1.00E-40 |
| WRIS_152 | GR303181 | 1 | 573 | retrotransposon protein, putative (O. sativa) | 1.00E-53 |
| WRIS_1563 | GR303195 | 1 | 594 | mitogen-activated kinase kinase kinase alpha (O. sativa) | 3.00E-67 |
| WRIS_1700 | GR303246 | 1 | 555 | Tankyrase 1, putative (O. sativa) | 7.00E-31 |
| WRIS_2209 | GR303453 | 1 | 678 | putative DNA helicase (O. sativa) | 2.00E-60 |
| WRIS_2893 | GR303741 | 1 | 761 | replication factor C large subunit (T. aestivum) | 3.00E-50 |
| WRIS_2994 | GR303790 | 1 | 690 | Cell division protease ftsH homolog (O. sativa) | 1.00E-113 |
| WRIS_317 | GR303871 | 1 | 362 | Cell division protease ftsH homolog (O. sativa) | 1.00E-23 |
| WRIS_3606 | GR304043 | 1 | 258 | DnaJ protein（Solanum tuberosum） | 5.00E-35 |
| WRIS_4441 | GR304326 | 1 | 657 | putative PHG1A protein (O. sativa) | 1.00E-89 |
| WRIS_4893 | GR304529 | 11 | 388 | putative DNA methyltransferase DMT106 (O. sativa) | 1.00E-07 |
| WRIS_5070 | GR304611 | 1 | 623 | putative small nuclear ribonucleoprotein polypeptide G (O. sativa) | 6.00E-35 |
| WRIS_5214 | GR304676 | 1 | 616 | putative chromodomain-helicase-DNA-binding protein （Arabidopsisthaliana） | 4.00E-51 |
| WRIS_5398 | GR304742 | 1 | 704 | DNA-binding protein S1FA2 (O. sativa) | 3.00E-11 |
| WRIS_5888 | GR304940 | 1 | 640 | J-domain protein (T. aestivum) | 1.00E-109 |
| WRIS_5956 | GR304969 | 1 | 472 | Cell division cycle protein 48 (O. sativa) | 1.00E-20 |
| **Secondary metabolism** | | | | | |
| WRIC_81 | GR302465 | 2 | 718 | betaine-aldehyde dehydrogenase (T. aestivum) | 1.00E-138 |
| WRIC_121 | GR302505 | 2 | 710 | carotenoid cleavage dioxygenase （Z. mays） | 4.00E-53 |
| WRIC_333 | GR302717 | 3 | 702 | phytoene synthase 2 (Z. mays) | 2.00E-90 |
| WRIC_417 | GR302801 | 3 | 715 | S-adenosylmethionine decarboxylase precursor (T. aestivum) | 1.00E-98 |
| WRIC_516 | GR302900 | 2 | 478 | carotenoid cleavage dioxygenase 1 (O. sativa) | 2.00E-43 |
| WRIC_550 | GR302934 | 2 | 630 | Mg-chelatase subunit XANTHA-F（Hordeum vulgare） | 1.00E-108 |
| WRIS_1280 | GR303085 | 1 | 518 | 24-methylene lophenol C24 methyltransferase (O. sativa) | 8.00E-18 |
| WRIS_1603 | GR303210 | 1 | 600 | CBS domain containing protein (O. sativa) | 2.00E-75 |
| WRIS_2068 | GR303401 | 1 | 512 | putative AdoMet synthase 4（H. vulgare） | 1.00E-59 |
| WRIS_2091 | GR303409 | 1 | 778 | putative prephenate dehydratase (O. sativa) | 2.00E-99 |
| WRIS_2260 | GR303475 | 1 | 556 | putative ethanolamine kinase 1 (O. sativa) | 3.00E-84 |
| WRIS_2752 | GR303686 | 1 | 456 | S-adenosylmethionine decarboxylase precursor (T. aestivum) | 3.00E-37 |
| WRIS_2795 | GR303703 | 1 | 748 | putative quinolinate phosphoribosyltransferase (Nicotiana tabacum) | 3.00E-55 |
| WRIS_2856 | GR303721 | 1 | 733 | flavonoid 7-O-methyltransferase-like (O. sativa) | 5.00E-46 |
| WRIS_3496 | GR303999 | 1 | 451 | tocopherol cyclase (T. aestivum) | 4.00E-60 |
| WRIS_4376 | GR304303 | 1 | 604 | Gamma interferon inducible lysosomal thiol reductase familyprotein (O. sativa) | 8.00E-43 |
| WRIS_4262 | GR304259 | 1 | 650 | Squalene monooxygenase, putative (O. sativa) | 1.00E-110 |
| WRIS_4799 | GR304477 | 1 | 479 | quinone reductase (Triticum monococcum) | 4.00E-54 |
| WRIS_4935 | GR304555 | 1 | 491 | putative nifU-like protein (O. sativa) | 2.00E-61 |
| WRIS_4980 | GR304573 | 1 | 626 | delta-24-sterol methyltransferase (T. aestivum) | 2.00E-84 |
| WRIS_5080 | GR304615 | 1 | 524 | remorin-like protein (O. sativa) | 2.00E-22 |
| WRIS_5115 | GR304633 | 1 | 723 | putative nicotinate phosphoribosyltransferase | 8.00E-65 |
| WRIS_5199 | GR304670 | 1 | 661 | cystathionine gamma-synthase (Z. mays) | 1.00E-113 |
| WRIS_5727 | GR304868 | 1 | 429 | Tropinone reductase (O. sativa) | 4.00E-21 |
| WRIS_5800 | GR304904 | 1 | 262 | maturase K (Triticum palmovae) | 1.00E-37 |
| WRIS_5892 | GR304942 | 1 | 368 | putative insulin degrading enzyme (O. sativa) | 1.00E-29 |
| WRIS_992 | GR305124 | 1 | 474 | putative 2'-hydroxyisoflavone reductase (O. sativa) | 3.00E-24 |
| WRIS_3148 | GR303859 | 1 | 346 | putative heme binding protein 2 (O. sativa) | 4.00E-17 |
| **Cell traffic** | | | | | |
| WRIC_254 | GR302638 | 2 | 591 | aerobic Mg-protoporphyrin IX monomethyl ester cyclase（Hordeumvulgare） | 1.00E-44 |
| WRIC_519 | GR302903 | 2 | 497 | nuclear transport factor 2 (O. sativa) | 5.00E-19 |
| WRIS_2445 | GR303556 | 1 | 736 | rab3-GAP regulatory domain-like (O. sativa) | 3.00E-93 |
| WRIS_3078 | GR303826 | 1 | 606 | putative vacuolar-type H+-translocating inorganic pyrophosphatase (O. sativa) | 2.00E-83 |
| WRIS_3256 | GR303900 | 1 | 655 | putative alpha-soluble NSF attachment protein (O. sativa) | 2.00E-72 |
| WRIS_3601 | GR304040 | 1 | 310 | putative ADP-ribosylation factor (O. sativa) | 6.00E-17 |
| WRIS_3801 | GR304111 | 1 | 580 | nuclear movement protein-like (O. sativa) | 5.00E-42 |
| WRIS_4185 | GR304235 | 1 | 548 | putative vacuolar ATP synthase subunit H (O. sativa) | 2.00E-33 |
| WRIS_4259 | GR304257 | 1 | 603 | SNF7 protein-like (O. sativa) | 1.00E-56 |
| WRIS_4 | GR304581 | 1 | 392 | putative Vacuolar ATP synthase subunit d (O. sativa) | 2.00E-21 |
| **Unknown** | | | | | |
| WRIC_11 | GR302395 | 2 | 588 | putative r40c2 protein (O. sativa) | 7.00E-33 |
| WRIC_21 | GR302405 | 2 | 983 | unknown protein (Arabidopsis thaliana) | 0.00E+00 |
| WRIC_27 | GR302411 | 2 | 358 | hypothetical protein (Phalaenopsis Aphrodite) | 4.00E-06 |
| WRIC_57 | GR302441 | 2 | 203 | unknown protein (A. thaliana) | 8.00E-17 |
| WRIC_72 | GR302456 | 4 | 1150 | hypothetical protein (O. sativa) | 2.00E-26 |
| WRIC_6 | GR302390 | 2 | 649 | Os05g0419200 (O. sativa) | 9.00E-19 |
| WRIC_9 | GR302393 | 2 | 844 | unknown protein (O. sativa) | 3.00E-71 |
| WRIC_70 | GR302454 | 17 | 2018 | P0519D04.20 (O. sativa) | 1.00E-15 |
| WRIC_111 | GR302495 | 3 | 343 | hypothetical protein (O. sativa) | 6.00E-23 |
| WRIC_113 | GR302497 | 3 | 561 | unknown protein (O. sativa) | 2.00E-27 |
| WRIC_161 | GR302545 | 2 | 716 | putative UOS1 (O. sativa) | 1.00E-106 |
| WRIC_418 | GR302802 | 2 | 683 | expressed protein (O. sativa) | 1.00E-70 |
| WRIC_169 | GR302553 | 2 | 1092 | expressed protein (O. sativa) | 1.00E-71 |
| WRIC_187 | GR302571 | 2 | 795 | expressed protein (O. sativa) | 7.00E-87 |
| WRIC_197 | GR302581 | 6 | 671 | hypothetical protein (Phalaenopsis Aphrodite) | 4.00E-47 |
| WRIC_204 | GR302588 | 2 | 820 | unknown protein (O. sativa) | 5.00E-42 |
| WRIC_224 | GR302608 | 2 | 956 | unknown protein (O. sativa) | 7.00E-64 |
| WRIC_236 | GR302620 | 3 | 621 | unknown protein (O. sativa) | 5.00E-13 |
| WRIC_248 | GR302632 | 2 | 746 | Os04g0653000 (O. sativa) | 6.00E-45 |
| WRIC_251 | GR302635 | 2 | 898 | | ORF42 (T. aestivum) | 2.00E-10 |
| WRIC_257 | GR302641 | 2 | 723 | Os04g0538100 (O. sativa) | 1.00E-91 |
| WRIC_275 | GR302659 | 2 | 565 | OSJNBa0036B21.24 (O. sativa) | 4.00E-43 |
| WRIC_229 | GR302613 | 2 | 1008 | unknown protein (Homo sapiens) | 1.00E-142 |
| WRIC_261 | GR302645 | 5 | 500 | Os05g0400800 (O. sativa) | 8.00E-24 |
| WRIC_265 | GR302649 | 150 | 1618 | hypothetical protein (Medicago truncatula) | 9.00E-31 |
| WRIC_273 | GR302657 | 2 | 1072 | OSJNBa0086B14.7 (O. sativa) | 7.00E-25 |
| WRIC_277 | GR302661 | 2 | 582 | Os02g0593400 (O. sativa) | 8.00E-28 |
| WRIC_282 | GR302666 | 5 | 648 | unknown protein (O. sativa) | 5.00E-34 |
| WRIC_285 | GR302669 | 2 | 707 | OJ991214_12.3 (O. sativa) | 1.00E-69 |
| WRIC_292 | GR302676 | 2 | 417 | P0031D02.12 (O. sativa) | 4.00E-28 |
| WRIC_342 | GR302726 | 2 | 711 | unknown protein (O. sativa) | 5.00E-50 |
| WRIC_363 | GR302747 | 2 | 617 | P0031D02.12 (O. sativa) | 4.00E-44 |
| WRIC_378 | GR302762 | 2 | 668 | P0520B06.24 (O. sativa) | 4.00E-66 |
| WRIC_396 | GR302780 | 2 | 430 | Os01g0896500 (O. sativa) | 5.00E-27 |
| WRIC_423 | GR302807 | 23 | 1223 | unknow protein (O. sativa) | 6.00E-15 |
| WRIC_499 | GR302883 | 3 | 1320 | unknown protein (A. thaliana) | 3.00E-89 |
| WRIC_509 | GR302893 | 2 | 768 | unknow protein (O. sativa) | 2.00E-54 |
| WRIC_515 | GR302899 | 2 | 669 | OJ991214_12.3 (O. sativa) | 1.00E-63 |
| WRIC_538 | GR302922 | 28 | 515 | hypothetical protein (Nicotiana tabacum) | 3.00E-20 |
| WRIC_545 | GR302929 | 3 | 641 | unknow protein (O. sativa) | 2.00E-26 |
| WRIC_578 | GR302962 | 3 | 708 | unknow protein (O. sativa) | 2.00E-34 |
| WRIS_1000 | GR302968 | 1 | 473 | unknown protein (A. thaliana) | 1.00E-24 |
| WRIS_1002 | GR302969 | 1 | 759 | unknow protein (O. sativa) | 2.00E-84 |
| WRIS_1006 | GR302971 | 1 | 503 | Os12g0529900 (O. sativa) | 4.00E-11 |
| WRIS_1026 | GR302980 | 1 | 538 | unknow protein (O. sativa) | 2.00E-23 |
| WRIS_1114 | GR303015 | 1 | 396 | unknown (T. aestivum) | 2.00E-30 |
| WRIS_1125 | GR303022 | 1 | 340 | OSJNBb0004G23.6 (O. sativa) | 9.00E-19 |
| WRIS_1129 | GR303024 | 1 | 453 | unknow protein (O. sativa) | 8.00E-20 |
| WRIS_1218 | GR303057 | 1 | 599 | Hypothetical protein (O. sativa) | 2.00E-25 |
| WRIS_122 | GR303060 | 1 | 583 | unknown protein (O. sativa) | 1.00E-56 |
| WRIS_1253 | GR303072 | 1 | 669 | unknown protein (O. sativa) | 9.00E-54 |
| WRIS_1447 | GR303147 | 1 | 609 | expressed protein (O. sativa) | 9.00E-37 |
| WRIS_1457 | GR303151 | 1 | 528 | unknown protein (O. sativa) | 3.00E-74 |
| WRIS_1514 | GR303175 | 1 | 609 | unknown protein (O. sativa) | 1.00E-31 |
| WRIS_1516 | GR303176 | 1 | 686 | OSJNBb0038F03.15 (O. sativa) | 4.00E-12 |
| WRIS_1538 | GR303183 | 1 | 388 | unknown protein (O. sativa) | 3.00E-30 |
| WRIS_1557 | GR303193 | 1 | 477 | Os04g0527500 (O. sativa) | 5.00E-22 |
| WRIS_1574 | GR303199 | 1 | 609 | unknown protein (O. sativa) | 1.00E-48 |
| WRIS_1582 | GR303200 | 1 | 577 | unknown protein (A. thaliana) | 2.00E-10 |
| WRIS_158 | GR303204 | 1 | 316 | Os06g0631300 (O. sativa) | 7.00E-22 |
| WRIS_162 | GR303223 | 1 | 156 | hypothetical protein (O. sativa) | 4.00E-16 |
| WRIS_1696 | GR303243 | 1 | 677 | hypothetical protein(P. aphrodite) | 5.00E-26 |
| WRIS_1725 | GR303256 | 1 | 736 | Os02g0533000 (O. sativa) | 1.00E-103 |
| WRIS_1993 | GR303370 | 1 | 669 | Os03g0388900 (O. sativa) | 9.00E-31 |
| WRIS_1730 | GR303258 | 1 | 501 | unnamed protein product (O. sativa) | 3.00E-17 |
| WRIS_1774 | GR303276 | 1 | 673 | unknown protein (O. sativa) | 2.00E-49 |
| WRIS_1783 | GR303284 | 1 | 621 | unknown protein (O. sativa) | 3.00E-54 |
| WRIS_1821 | GR303301 | 1 | 761 | unknown protein (O. sativa) | 1.00E-117 |
| WRIS_1842 | GR303310 | 1 | 730 | unknown protein (O. sativa) | 1.00E-101 |
| WRIS_185 | GR303319 | 1 | 488 | hypothetical protein (O. sativa) | 2.00E-11 |
| WRIS_1862 | GR303320 | 1 | 627 | unknown protein (O. sativa) | 7.00E-61 |
| WRIS_1863 | GR303321 | 1 | 599 | hypothetical protein (Medicago truncatula) | 2.00E-25 |
| WRIS_1876 | GR303327 | 1 | 436 | expressed protein (O. sativa) | 2.00E-19 |
| WRIS_1992 | GR303369 | 1 | 667 | unknown protein (O. sativa) | 7.00E-28 |
| WRIS_2020 | GR303383 | 1 | 505 | Os11g0216300 (O. sativa) | 1.00E-30 |
| WRIS_2037 | GR303387 | 1 | 615 | expressed protein (O. sativa) | 1.00E-29 |
| WRIS_2055 | GR303392 | 1 | 605 | unknown protein (O. sativa) | 2.00E-62 |
| WRIS_2058 | GR303394 | 1 | 586 | unknown protein (O. sativa) | 3.00E-29 |
| WRIS_2083 | GR303406 | 1 | 527 | Os04g0692200 (O. sativa) | 3.00E-16 |
| WRIS_2107 | GR303414 | 1 | 538 | unnamed protein product (O. sativa) | 1.00E-37 |
| WRIS_212 | GR303425 | 1 | 753 | unknown protein (O. sativa) | 3.00E-60 |
| WRIS_2164 | GR303437 | 1 | 498 | Os02g0131100 (O. sativa) | 5.00E-27 |
| WRIS_2182 | GR303444 | 1 | 672 | unknown protein (O. sativa) | 2.00E-23 |
| WRIS_2219 | GR303458 | 1 | 535 | unknown protein (O. sativa) | 6.00E-47 |
| WRIS_2241 | GR303467 | 1 | 384 | unknown protein (O. sativa) | 6.00E-26 |
| WRIS_2248 | GR303470 | 1 | 636 | unknown protein (O. sativa) | 8.00E-62 |
| WRIS_2297 | GR303489 | 1 | 722 | Os01g0170600 (O. sativa) | 2.00E-69 |
| WRIS_2405 | GR303539 | 1 | 446 | Os06g0163200 (O. sativa) | 2.00E-46 |
| WRIS_2441 | GR303555 | 1 | 730 | expressed protein (O. sativa) | 2.00E-26 |
| WRIS_245 | GR303563 | 1 | 657 | unknown protein (O. sativa) | 1.00E-76 |
| WRIS_2504 | GR303584 | 1 | 309 | unknown protein (O. sativa) | 6.00E-12 |
| WRIS_2623 | GR303626 | 1 | 715 | unknown (Bambusa ventricosa) | 2.00E-28 |
| WRIS_2649 | GR303633 | 1 | 524 | unknown protein (O. sativa) | 2.00E-45 |
| WRIS_2661 | GR303638 | 1 | 669 | expressed protein (O. sativa) | 7.00E-71 |
| WRIS_268 | GR303655 | 1 | 537 | unknown protein (O. sativa) | 1.00E-34 |
| WRIS_2755 | GR303688 | 1 | 509 | unknown protein (O. sativa) | 7.00E-42 |
| WRIS_2776 | GR303694 | 1 | 731 | unknown protein (O. sativa) | 6.00E-37 |
| WRIS_2816 | GR303709 | 1 | 417 | P0497A05.16 (O. sativa) | 5.00E-14 |
| WRIS_2820 | GR303711 | 1 | 656 | unknown protein (O. sativa) | 9.00E-86 |
| WRIS_2839 | GR303716 | 1 | 604 | hypothetical protein (O. sativa) | 1.00E-09 |
| WRIS_2841 | GR303717 | 1 | 773 | unknown protein (O. sativa) | 1.00E-74 |
| WRIS_2844 | GR303719 | 1 | 632 | unknown protein (O. sativa) | 2.00E-34 |
| WRIS_2873 | GR303728 | 1 | 542 | expressed protein (O. sativa) | 1.00E-23 |
| WRIS_2919 | GR303753 | 1 | 772 | OSJNBb0096E05.17 (O. sativa) | 3.00E-25 |
| WRIS_2921 | GR303755 | 1 | 673 | Os01g0337600 (O. sativa) | 1.00E-71 |
| WRIS_2968 | GR303774 | 1 | 475 | unknown protein (O. sativa) | 4.00E-22 |
| WRIS_297 | GR303779 | 1 | 648 | Os07g0181800 (O. sativa) | 6.00E-13 |
| WRIS_2985 | GR303783 | 1 | 272 | hypothetical protein(Medicago truncatula) | 7.00E-08 |
| WRIS_3018 | GR303800 | 1 | 723 | Os01g0898800 (O. sativa) | 7.00E-13 |
| WRIS_3020 | GR303801 | 1 | 665 | Os11g0689400 (O. sativa) | 1.00E-13 |
| WRIS_3067 | GR303822 | 1 | 499 | unknown protein (O. sativa) | 9.00E-20 |
| WRIS_3097 | GR303835 | 1 | 693 | P0492F05.13 (O. sativa) | 7.00E-36 |
| WRIS_3131 | GR303853 | 1 | 612 | unknown protein (O. sativa) | 2.00E-62 |
| WRIS_3171 | GR303866 | 1 | 506 | unknown protein (O. sativa) | 0.00E+00 |
| WRIS_3221 | GR303887 | 1 | 589 | unknown protein (O. sativa) | 1.00E-46 |
| WRIS_322 | GR303889 | 1 | 682 | unknown protein (O. sativa) | 8.00E-67 |
| WRIS_3244 | GR303894 | 1 | 471 | unknown protein (O. sativa) | 4.00E-36 |
| WRIS_3268 | GR303904 | 1 | 651 | unknown protein (O. sativa) | 6.00E-08 |
| WRIS_3294 | GR303917 | 1 | 722 | unknown protein (O. sativa) | 1.00E-22 |
| WRIS_3349 | GR303939 | 1 | 533 | Os10g0489700 (O. sativa) | 2.00E-12 |
| WRIS_3356 | GR303941 | 1 | 556 | unknown protein (O. sativa) | 5.00E-41 |
| WRIS_3368 | GR303946 | 1 | 741 | unknown protein (O. sativa) | 2.00E-59 |
| WRIS_3378 | GR303948 | 1 | 496 | unknown protein (O. sativa) | 4.00E-46 |
| WRIS_3388 | GR303954 | 1 | 691 | unknown protein (O. sativa) | 5.00E-67 |
| WRIS_3418 | GR303967 | 1 | 525 | hypothetical protein (Nicotiana tabacum) | 3.00E-20 |
| WRIS_3425 | GR303970 | 1 | 623 | unknown protein (O. sativa) | 7.00E-10 |
| WRIS_3474 | GR303992 | 1 | 498 | unknown protein (O. sativa) | 2.00E-14 |
| WRIS_3483 | GR303994 | 1 | 716 | unknown protein (O. sativa) | 4.00E-51 |
| WRIS_3491 | GR303998 | 1 | 657 | expressed protein (O. sativa) | 7.00E-38 |
| WRIS_3498 | GR304000 | 1 | 708 | hypothetical protein (Phalaenopsis aphrodite) | 1.00E-46 |
| WRIS_3514 | GR304005 | 1 | 679 | unknown protein (O. sativa) | 3.00E-98 |
| WRIS_3518 | GR304007 | 1 | 738 | unknown protein (O. sativa) | 3.00E-47 |
| WRIS_3590 | GR304035 | 1 | 606 | unknown protein (O. sativa) | 2.00E-25 |
| WRIS_1096 | GR303006 | 1 | 707 | OSJNBa0020P07.9 (O. sativa) | 1.00E-07 |
| WRIS_3618 | GR304048 | 1 | 495 | Os04g0477900 (O. sativa) | 6.00E-14 |
| WRIS_3632 | GR304057 | 1 | 587 | OSJNBb0022F16.14 (O. sativa) | 2.00E-28 |
| WRIS_3643 | GR304061 | 1 | 590 | unknown protein (O. sativa) | 2.00E-15 |
| WRIS_3652 | GR304064 | 1 | 740 | unknown protein (O. sativa) | 1.00E-76 |
| WRIS_3691 | GR304077 | 1 | 541 | expressed protein (O. sativa) | 4.00E-41 |
| WRIS_3697 | GR304079 | 1 | 542 | Os05g0566600 (O. sativa) | 6.00E-11 |
| WRIS_3722 | GR304090 | 1 | 501 | unknown protein (O. sativa) | 3.00E-29 |
| WRIS_3731 | GR304093 | 1 | 532 | unknown protein (O. sativa) | 2.00E-28 |
| WRIS_4103 | GR304200 | 1 | 519 | Os09g0542100 (O. sativa) | 3.00E-07 |
| WRIS_3745 | GR304098 | 1 | 378 | unknown protein (O. sativa) | 6.00E-15 |
| WRIS_3823 | GR304116 | 1 | 625 | unknown protein (O. sativa) | 6.00E-60 |
| WRIS_3822 | GR304115 | 1 | 679 | unknown protein (O. sativa) | 5.00E-43 |
| WRIS_3846 | GR304126 | 1 | 589 | unknown protein (O. sativa) | 5.00E-47 |
| WRIS_3861 | GR304132 | 1 | 411 | unknown protein (O. sativa) | 1.00E-20 |
| WRIS_4037 | GR304177 | 1 | 336 | unknown protein (O. sativa) | 0.00E+00 |
| WRIS_4040 | GR304178 | 1 | 622 | expressed protein (O. sativa) | 8.00E-78 |
| WRIS_4051 | GR304180 | 1 | 705 | unknown protein (O. sativa) | 3.00E-68 |
| WRIS_4135 | GR304213 | 1 | 354 | unknown protein (O. sativa) | 4.00E-09 |
| WRIS_4145 | GR304219 | 1 | 558 | expressed protein (O. sativa) | 2.00E-41 |
| WRIS_4160 | GR304227 | 1 | 653 | expressed protein (O. sativa) | 2.00E-26 |
| WRIS_416 | GR304230 | 1 | 676 | unknown protein (O. sativa) | 6.00E-11 |
| WRIS_4183 | GR304234 | 1 | 661 | Os04g0535400 (O. sativa) | 6.00E-69 |
| WRIS_4295 | GR304270 | 1 | 583 | OSJNBb0065L13.4 (O. sativa) | 5.00E-26 |
| WRIS_4307 | GR304274 | 1 | 455 | unknown protein (O. sativa) | 5.00E-07 |
| WRIS_4326 | GR304285 | 1 | 574 | unknown protein (O. sativa) | 6.00E-08 |
| WRIS_4328 | GR304286 | 1 | 647 | hypothetical protein (O. sativa) | 4.00E-27 |
| WRIS_4335 | GR304289 | 1 | 592 | unknown protein (O. sativa) | 3.00E-35 |
| WRIS_434 | GR304294 | 1 | 405 | Hypothetical protein (O. sativa) | 1.00E-41 |
| WRIS_4382 | GR304304 | 1 | 492 | Hypothetical protein (O. sativa) | 6.00E-72 |
| WRIS_4395 | GR304312 | 1 | 443 | unknown protein (O. sativa) | 1.00E-11 |
| WRIS_4443 | GR304328 | 1 | 497 | unknown protein (O. sativa) | 9.00E-21 |
| WRIS_4452 | GR304331 | 1 | 722 | unknown protein (O. sativa) | 0.00E+00 |
| WRIS_4398 | GR304313 | 1 | 674 | Os09g0493700 (O. sativa) | 8.00E-58 |
| WRIS_4399 | GR304314 | 1 | 298 | OSJNBa0008A08.5 (O. sativa) | 3.00E-06 |
| WRIS_448 | GR304347 | 1 | 512 | OSJNBa0068L06.8 (O. sativa) | 1.00E-32 |
| WRIS_4500 | GR304352 | 1 | 679 | unknown protein (O. sativa) | 1.00E-112 |
| WRIS_4505 | GR304355 | 1 | 685 | hypothetical protein (Brachypodium sylvaticum) | 4.00E-22 |
| WRIS_4523 | GR304362 | 1 | 711 | Hypothetical protein (O. sativa) | 9.00E-80 |
| WRIS_4533 | GR304366 | 1 | 612 | unknown protein (O. sativa) | 2.00E-39 |
| WRIS_4551 | GR304374 | 1 | 660 | unknown protein (O. sativa) | 2.00E-29 |
| WRIS_4557 | GR304378 | 1 | 645 | expressed protein (O. sativa) | 2.00E-16 |
| WRIS_455 | GR304380 | 1 | 793 | expressed protein (O. sativa) | 7.00E-73 |
| WRIS_4582 | GR304390 | 1 | 676 | unknown protein (O. sativa) | 2.00E-59 |
| WRIS_4588 | GR304393 | 1 | 674 | Os08g0242700 (O. sativa) | 0.00E+00 |
| WRIS_45 | GR304397 | 1 | 597 | Os06g0107000 (O. sativa) | 1.00E-10 |
| WRIS_4601 | GR304399 | 1 | 653 | expressed protein (O. sativa) | 2.00E-30 |
| WRIS_465 | GR304417 | 1 | 374 | unknown protein (O. sativa) | 5.00E-30 |
| WRIS_2503 | GR303583 | 1 | 636 | OSJNBb0012E08.10 (O. sativa) | 6.00E-20 |
| WRIS_2526 | GR303594 | 1 | 432 | OSJNBa0088I22.11 (O. sativa) | 1.00E-21 |
| WRIS_3081 | GR303827 | 1 | 699 | OSJNBa0009P12.21 (O. sativa) | 0.00E+00 |
| WRIS_3891 | GR304143 | 1 | 535 | unknown protein (A. thaliana) | 0.00E+00 |
| WRIS_4709 | GR304432 | 1 | 664 | unknown protein (O. sativa) | 4.00E-66 |
| WRIS_4708 | GR304431 | 1 | 502 | hypothetical protein (Phalaenopsis aphrodite) | 2.00E-47 |
| WRIS_4745 | GR304453 | 1 | 344 | hypothetical protein (Phalaenopsis aphrodite) | 2.00E-27 |
| WRIS_4775 | GR304463 | 1 | 410 | unknown protein (O. sativa) | 3.00E-10 |
| WRIS_4781 | GR304467 | 1 | 428 | hypothetical protein (U. maydis) | 7.00E-19 |
| WRIS_4797 | GR304476 | 1 | 377 | unknown protein (O. sativa) | 2.00E-36 |
| WRIS_4833 | GR304496 | 1 | 584 | hypothetical protein (Medicago truncatula) | 1.00E-24 |
| WRIS_4847 | GR304502 | 1 | 704 | unknown protein (O. sativa) | 3.00E-41 |
| WRIS_4866 | GR304517 | 1 | 677 | hypothetical protein (Phalaenopsis aphrodite) | 4.00E-47 |
| WRIS_4924 | GR304550 | 1 | 531 | Os05g0305100 (O. sativa) | 2.00E-21 |
| WRIS_4961 | GR304564 | 1 | 580 | unknown protein (O. sativa) | 9.00E-25 |
| WRIS_4968 | GR304569 | 1 | 719 | Os07g0223700 (O. sativa) | 2.00E-29 |
| WRIS_4973 | GR304572 | 1 | 717 | Os01g0171800 (O. sativa) | 1.00E-83 |
| WRIS_4986 | GR304574 | 1 | 729 | unknown protein (O. sativa) | 1.00E-110 |
| WRIS_499 | GR304580 | 1 | 555 | unknown protein (O. sativa) | 3.00E-37 |
| WRIS_5002 | GR304582 | 1 | 341 | unknown protein (O. sativa) | 2.00E-09 |
| WRIS_5005 | GR304584 | 1 | 629 | Os01g0272800 (O. sativa) | 1.00E-62 |
| WRIS_5012 | GR304587 | 1 | 646 | unknown protein (O. sativa) | 8.00E-20 |
| WRIS_5055 | GR304608 | 1 | 665 | unknown protein (A. thaliana) | 1.00E-12 |
| WRIS_5071 | GR304612 | 1 | 556 | expressed protein (O. sativa) | 1.00E-39 |
| WRIS_2177 | GR303441 | 1 | 587 | unknown protein (O. sativa) | 5.00E-17 |
| WRIS_511 | GR304637 | 1 | 440 | unknown protein (O. sativa) | 1.00E-17 |
| WRIS_5158 | GR304649 | 1 | 330 | unknown protein (O. sativa) | 1.00E-45 |
| WRIS_5192 | GR304666 | 1 | 370 | unknown protein (O. sativa) | 4.00E-24 |
| WRIS_5164 | GR304653 | 1 | 712 | unknown protein (O. sativa) | 1.00E-33 |
| WRIS_5181 | GR304661 | 1 | 730 | Os07g0646100 (O. sativa) | 9.00E-06 |
| WRIS_5198 | GR304669 | 1 | 699 | expressed protein (O. sativa) | 2.00E-59 |
| WRIS_5248 | GR304686 | 1 | 496 | unknown protein (O. sativa) | 3.00E-16 |
| WRIS_5266 | GR304694 | 1 | 232 | Os07g0673000 (O. sativa) | 2.00E-12 |
| WRIS_5315 | GR304711 | 1 | 671 | hypothetical protein (Phalaenopsis aphrodite) | 0.00E+00 |
| WRIS_5367 | GR304728 | 1 | 582 | unknown protein (O. sativa) | 6.00E-26 |
| WRIS_5368 | GR304729 | 1 | 283 | Os05g0419200 (O. sativa) | 1.00E-38 |
| WRIS_5881 | GR304937 | 1 | 404 | hypothetical protein (O. sativa) | 4.00E-06 |
| WRIS_537 | GR304736 | 1 | 635 | OSE2-like protein (O. sativa) | 2.00E-20 |
| WRIS_5442 | GR304761 | 1 | 757 | OSJNBb0004A17.6 (O. sativa) | 1.00E-117 |
| WRIS_5481 | GR304775 | 1 | 680 | Os08g0567000 (O. sativa) | 2.00E-82 |
| WRIS_5506 | GR304786 | 1 | 677 | putative OsCTTP (O. sativa) | 1.00E-46 |
| WRIS_5531 | GR304797 | 1 | 639 | OSJNBa0093F12.3 (O. sativa) | 2.00E-75 |
| WRIS_5618 | GR304830 | 1 | 696 | OSJNBa0069D17.9 (O. sativa) | 6.00E-16 |
| WRIS_5631 | GR304836 | 1 | 608 | unknown protein (O. sativa) | 2.00E-11 |
| WRIS_5652 | GR304846 | 1 | 428 | unknown protein (O. sativa) | 2.00E-38 |
| WRIS_5688 | GR304858 | 1 | 630 | expressed protein (O. sativa) | 1.00E-54 |
| WRIS_5690 | GR304860 | 1 | 669 | unknown protein (O. sativa) | 2.00E-20 |
| WRIS_5718 | GR304864 | 1 | 733 | unknown protein (O. sativa) | 5.00E-54 |
| WRIS_571 | GR304866 | 1 | 672 | Os05g0401200 (O. sativa) | 2.00E-61 |
| WRIS_5729 | GR304869 | 1 | 398 | expressed protein (O. sativa) | 0.00E+00 |
| WRIS_5742 | GR304874 | 1 | 666 | Os03g0766000 (O. sativa) | 1.00E-28 |
| WRIS_216 | GR303438 | 1 | 321 | Os06g0548100 (O. sativa) | 4.00E-19 |
| WRIS_5752 | GR304878 | 1 | 787 | OSJNBa0084K01.13 (O. sativa) | 0.00E+00 |
| WRIS_5773 | GR304887 | 1 | 544 | unknown protein (O. sativa) | 4.00E-14 |
| WRIS_57 | GR304903 | 1 | 453 | unknown (Deschampsia antarctica) | 7.00E-14 |
| WRIS_5823 | GR304911 | 1 | 456 | Os01g0929100 (O. sativa) | 2.00E-25 |
| WRIS_5835 | GR304915 | 1 | 714 | unknown protein (O. sativa) | 8.00E-23 |
| WRIS_5839 | GR304917 | 1 | 682 | P0520B06.24 (O. sativa) | 2.00E-81 |
| WRIS_5849 | GR304923 | 1 | 533 | unknown protein (O. sativa) | 6.00E-18 |
| WRIS_5862 | GR304928 | 1 | 744 | Os01g0104100 (O. sativa) | 1.00E-113 |
| WRIS_5863 | GR304929 | 1 | 704 | Os09g0436900 (O. sativa) | 5.00E-45 |
| WRIS_5897 | GR304947 | 1 | 573 | OSJNBa0011L07.8 (O. sativa) | 2.00E-70 |
| WRIS_5952 | GR304967 | 1 | 674 | unknown protein (O. sativa) | 1.00E-44 |
| WRIS_5969 | GR304974 | 1 | 364 | Os03g0669600 (O. sativa) | 2.00E-25 |
| WRIS_597 | GR304979 | 1 | 536 | Os11g0148200 (O. sativa) | 1.00E-38 |
| WRIS_601 | GR304989 | 1 | 594 | OJ000114_01.6 (O. sativa) | 0.00E+00 |
| WRIS_615 | GR304996 | 1 | 308 | unknown protein (O. sativa) | 6.00E-12 |
| WRIS_634 | GR305002 | 1 | 608 | Os04g0464500 (O. sativa) | 9.00E-42 |
| WRIS_650 | GR305005 | 1 | 591 | P0679C12.27 (O. sativa) | 2.00E-50 |
| WRIS_737 | GR305032 | 1 | 556 | unknown protein (O. sativa) | 0.00E+00 |
| WRIS_750 | GR305033 | 1 | 415 | hypothetical protein (O. sativa) | 9.00E-16 |
| WRIS_758 | GR305035 | 1 | 361 | OSJNBa0004N05.24 (O. sativa) | 0.00E+00 |
| WRIS_776 | GR305042 | 1 | 510 | unknown protein (O. sativa) | 6.00E-54 |
| WRIS_790 | GR305047 | 1 | 654 | expressed protein (O. sativa) | 1.00E-32 |
| WRIS_793 | GR305048 | 1 | 729 | unknown protein (O. sativa) | 7.00E-77 |
| WRIS_830 | GR305064 | 1 | 627 | unnamed protein product (O. sativa) | 1.00E-37 |
| WRIS_876 | GR305083 | 1 | 362 | expressed protein (O. sativa) | 4.00E-19 |
| WRIS_895 | GR305088 | 1 | 275 | Os07g0658400 (O. sativa) | 2.00E-34 |
| WRIS_902 | GR305090 | 1 | 373 | unknown protein (O. sativa) | 7.00E-06 |
| WRIS_918 | GR305095 | 1 | 651 | Os04g0482800 (O. sativa) | 2.00E-06 |
| WRIS_921 | GR305098 | 1 | 427 | unknown protein (O. sativa) | 2.00E-14 |
| WRIS_983 | GR305120 | 1 | 280 | OSJNBa0083N12.23 (O. sativa) | 3.00E-08 |
| WRIC_245 | GR302629 |  | 613 | Os08g0157600 (O. sativa) | 2.00E-30 |
| WRIS_1127 | GR303023 | 1 | 406 | Os03g0792400 (O. sativa) | 9.00E-14 |
| WRIS_1442 | GR303146 | 1 | 592 | Os04g0398300 (O. sativa) | 1.00E-78 |
| WRIS_218 | GR303447 | 1 | 399 | Os07g0693700 (O. sativa) | 1.00E-19 |
| WRIS_3568 | GR304031 | 1 | 680 | Os05g0373700 (O. sativa) | 2.00E-42 |
| WRIS_4244 | GR304252 | 1 | 340 | BAF04439.1 (O. sativa) | 6.00E-09 |
| WRIS_4534 | GR304367 | 1 | 622 | Os02g0722700 (O. sativa) | 3.00E-43 |
| WRIS_692 | GR305019 | 1 | 393 | OSJNBa0014K14.6 (O. sativa) | 6.00E-41 |
| WRIS_713 | GR305027 | 1 | 693 | Os11g0174000 (O. sativa) | 6.00E-75 |
| WRIS_769 | GR305039 | 1 | 679 | Os02g0326200 (O. sativa) | 5.00E-40 |
| WRIS_110 | GR303014 | 1 | 682 | Os04g0680400 (O. sativa) | 1.00E-112 |
| WRIS_1913 | GR303345 | 1 | 700 | OSJNBb0026L04.11 (O. sativa) | 2.00E-53 |
| WRIS_2857 | GR303722 | 1 | 855 | unknown protein (O. sativa) | 5.00E-26 |
| WRIS_4211 | GR304242 | 1 | 587 | OSJNBa0084K20.14 (O. sativa) | 1.00E-60 |
| WRIS_549 | GR304781 | 1 | 647 | OSJNBa0073E02.3 (O. sativa) | 3.00E-97 |
| WRIS_5548 | GR304803 | 1 | 601 | Os05g0401000 (O. sativa) | 5.00E-71 |
| WRIS_5615 | GR304828 | 1 | 488 | Os05g0150000 (O. sativa) | 6.00E-26 |
| WRIS_5998 | GR304988 | 1 | 751 | Os09g0511600 (O. sativa) | 1.00E-100 |
| WRIC_309 | GR302693 | 2 | 858 | Os05g0405500 (O. sativa) | 1.00E-59 |
| WRIS_4137 | GR304214 | 1 | 593 | OSJNBa0010H02.6 (O. sativa) | 3.00E-32 |
| WRIC_397 | GR302781 |  | 766 | OSJNBa0083N12.23 (O. sativa) | 3.00E-27 |
| WRIS_3119 | GR303847 | 1 | 309 | Os05g0350600 (O. sativa) | 2.00E-48 |
| WRIS_412 | GR304211 | 1 | 545 | OSJNBb0012E08.10 (O. sativa) | 1.00E-93 |
| WRIC_91 | GR302475 | 41 | 952 | hypothetical protein (Nicotiana tabacum) | 1.00E-28 |
| WRIC_297 | GR302681 | 6 | 369 | PREDICTED OJ1582_D10.6 gene product (O. sativa) | 1.00E-16 |
| WRIS_5361 | GR304726 | 1 | 556 | OSJNBa0084K11.9 (O. sativa) | 5.00E-66 |
| WRIS_2258 | GR303473 | 1 | 715 | Os05g0513900 (O. sativa) | 7.00E-87 |
| WRIS_2933 | GR303759 | 1 | 521 | conserved hypothetical protein (O. sativa) | 9.00E-11 |
| WRIC_143 | GR302527 | 8 | 673 | hypothetical protein OeelhCp025 (Oenothera elata) | 4.00E-07 |
| WRIC_171 | GR302555 | 2 | 667 | Os04g0311400 | 0.00E+00 |
| WRIC_232 | GR302616 | 2 | 797 | Os03g0571900 (O. sativa) | 3.00E-70 |
| WRIC_435 | GR302819 | 2 | 571 | OSJNBa0088A01.11 (O. sativa) | 0.00E+00 |
| WRIC_468 | GR302852 | 2 | 421 | OSJNBa0045O17.11 (O. sativa) | 5.00E-15 |
| WRIC_553 | GR302937 | 2 | 507 | Os09g0511600 (O. sativa) | 2.00E-14 |
| WRIS_1035 | GR302983 | 1 | 731 | OSJNBa0060P14.10 (O. sativa) | 4.00E-07 |
| WRIS_1100 | GR303009 | 1 | 600 | OSJNBa0017B10.14 (O. sativa) | 1.00E-85 |
| WRIS_1123 | GR303021 | 1 | 625 | OSJNBb0118P14.8 (O. sativa) | 1.00E-91 |
| WRIS_1167 | GR303039 | 1 | 638 | Os08g0518300 (O. sativa) | 1.00E-36 |
| WRIS_119 | GR303053 | 1 | 678 | Os05g0463400 (O. sativa) | 3.00E-39 |
| WRIS_1203 | GR303054 | 1 | 594 | OSJNBa0041A02.21 (O. sativa) | 1.00E-23 |
| WRIS_129 | GR303091 | 1 | 787 | Os04g0542900 (O. sativa) | 1.00E-116 |
| WRIS_1051 | GR302987 | 1 | 377 | expressed protein (O. sativa) | 2.00E-17 |
| WRIS_1458 | GR303152 | 1 | 400 | expressed protein (O. sativa) | 1.00E-13 |
| WRIS_1490 | GR303165 | 1 | 582 | Os04g0662700 (O. sativa) | 5.00E-59 |
| WRIS_1598 | GR303206 | 1 | 577 | Os04g0137500 (O. sativa) | 6.00E-48 |
| WRIS_1760 | GR303270 | 1 | 627 | Os04g0479200 (O. sativa) | 3.00E-39 |
| WRIS_1763 | GR303271 | 1 | 727 | Os11g0446500 (O. sativa) | 3.00E-55 |
| WRIS_1786 | GR303286 | 1 | 676 | Os02g0473000 (O. sativa) | 1.00E-105 |
| WRIS_4552 | GR304375 | 1 | 462 | putative protein (Arabidopsis thaliana) | 3.00E-31 |
| WRIS_1409 | GR303136 | 1 | 286 | Os05g0293600 (O. sativa) | 3.00E-06 |
| WRIS_1627 | GR303221 | 1 | 415 | Os11g0456100 (O. sativa) | 4.00E-32 |
| WRIS_1764 | GR303272 | 1 | 294 | Os09g0545300 (O. sativa) | 9.00E-08 |
| WRIS_1805 | GR303293 | 1 | 291 | OSJNBa0053K19.11 (O. sativa) | 8.00E-07 |
| WRIS_1848 | GR303312 | 1 | 454 | Os02g0439700 (O. sativa) | 4.00E-12 |
| WRIS_1856 | GR303316 | 1 | 626 | Os04g0602300 (O. sativa) | 5.00E-38 |
| WRIS_1883 | GR303330 | 1 | 665 | Os01g0896500 (O. sativa) | 1.00E-103 |
| WRIS_1930 | GR303353 | 1 | 620 | H0413E07.8 (O. sativa) | 6.00E-17 |
| WRIS_1957 | GR303358 | 1 | 671 | Os05g0149600 (O. sativa) | 1.00E-100 |
| WRIS_196 | GR303363 | 1 | 685 | Os09g0504700 (O. sativa) | 1.00E-23 |
| WRIS_1990 | GR303368 | 1 | 641 | OSJNBa0083N12.23 (O. sativa) | 1.00E-26 |
| WRIS_2060 | GR303395 | 1 | 500 | Os02g0820700 (O. sativa) | 1.00E-37 |
| WRIS_2256 | GR303472 | 1 | 622 | Os01g0896500 (O. sativa) | 3.00E-08 |
| WRIS_2261 | GR303476 | 1 | 530 | Os08g0545700 (O. sativa) | 9.00E-57 |
| WRIS_2319 | GR303501 | 1 | 747 | hypothetical protein (O. sativa) | 3.00E-37 |
| WRIS_2342 | GR303510 | 1 | 402 | OSJNBa0006A01.6 (O. sativa) | 6.00E-12 |
| WRIS_2347 | GR303513 | 1 | 679 | Os11g0148200 (O. sativa) | 6.00E-40 |
| WRIS_2371 | GR303524 | 1 | 337 | Os12g0514100 (O. sativa) | 4.00E-08 |
| WRIS_2411 | GR303543 | 1 | 726 | Os05g0401200 (O. sativa) | 2.00E-65 |
| WRIS_2566 | GR303609 | 1 | 315 | OSJNBa0071I13.18 (O. sativa) | 1.00E-09 |
| WRIS_2598 | GR303616 | 1 | 722 | OSJNBb0088C09.11 (O. sativa) | 2.00E-20 |
| WRIS_2666 | GR303642 | 1 | 572 | Os05g0311500 (O. sativa) | 2.00E-08 |
| WRIS_2716 | GR303667 | 1 | 657 | Os04g0450200 (O. sativa) | 2.00E-71 |
| WRIS_2727 | GR303670 | 1 | 734 | OSJNBa0008A08.11 (O. sativa) | 1.00E-114 |
| WRIS_2753 | GR303687 | 1 | 548 | OSJNBb0024F06.20 (O. sativa) | 1.00E-18 |
| WRIS_3082 | GR303828 | 1 | 672 | Os08g0170200 (O. sativa) | 4.00E-08 |
| WRIS_3090 | GR303833 | 1 | 275 | Os11g0433500 (O. sativa) | 2.00E-13 |
| WRIS_3130 | GR303852 | 1 | 729 | H0811E11.3 (O. sativa) | 0.00E+00 |
| WRIS_3159 | GR303862 | 1 | 441 | OSJNBb0024F06.14 (O. sativa) | 6.00E-14 |
| WRIS_3175 | GR303868 | 1 | 480 | Os03g0324300 (O. sativa) | 3.00E-11 |
| WRIS_3197 | GR303881 | 1 | 570 | OSJNBa0087O24.7 (O. sativa) | 3.00E-22 |
| WRIS_3429 | GR303972 | 1 | 447 | Os01g0267900 (O. sativa) | 3.00E-23 |
| WRIS_3488 | GR303995 | 1 | 688 | OSJNBb0118P14.12 (O. sativa) | 1.00E-108 |
| WRIS_4466 | GR304335 | 1 | 520 | Os01g0267900 (O. sativa) | 6.00E-10 |
| WRIS_4823 | GR304491 | 1 | 231 | Os06g0470000 (O. sativa) | 1.00E-30 |
| WRIS_5094 | GR304622 | 1 | 142 | Os09g0513000 (O. sativa) | 2.00E-09 |
| WRIC_326 | GR302710 | 6 | 923 | Os03g0388900 (O. sativa) | 2.00E-75 |
| WRIS_2159 | GR303435 | 1 | 514 | Os08g0119800 (O. sativa) | 2.00E-07 |
| WRIS_2308 | GR303496 | 1 | 623 | OSJNBa0036B21.24 (O. sativa) | 5.00E-43 |
| WRIS_2559 | GR303606 | 1 | 388 | Os05g0301700 (O. sativa) | 6.00E-13 |
| WRIS_3002 | GR303792 | 1 | 451 | H0212B02.14 (O. sativa) | 9.00E-35 |
| WRIS_1564 | GR303196 | 1 | 669 | Os04g0397100 (O. sativa) | 2.00E-67 |
| WRIS_515 | GR304651 | 1 | 704 | OSJNBa0032F06.20 (O. sativa) | 2.00E-95 |
| WRIS_5519 | GR304791 | 1 | 631 | OSJNBa0070C17.12 (O. sativa) | 2.00E-84 |
| WRIS_5648 | GR304844 | 1 | 555 | OSJNBa0083D01.7 (O. sativa) | 7.00E-10 |
| WRIS_5668 | GR304852 | 1 | 391 | Os06g0620600 (O. sativa) | 6.00E-12 |
| WRIS_5743 | GR304875 | 1 | 614 | Os06g0605900 (O. sativa) | 4.00E-43 |
| WRIS_5889 | GR304941 | 1 | 373 | OSJNBb0060M15.6 (O. sativa) | 5.00E-28 |
| WRIS_5936 | GR304962 | 1 | 447 | B1078G07.34 (O. sativa) | 7.00E-35 |
| WRIS_5940 | GR304963 | 1 | 514 | Os03g0229600 (O. sativa) | 3.00E-52 |
| WRIS_69 | GR305021 | 1 | 675 | Os01g0766600 (O. sativa) | 2.00E-25 |
| WRIS_980 | GR305119 | 1 | 302 | Os07g0476200 (O. sativa) | 7.00E-11 |
| WRIS_99 | GR305127 | 1 | 563 | Os08g0162100 (O. sativa) | 2.00E-16 |
| WRIS_3351 | GR303940 | 1 | 661 | Os03g0388900 (O. sativa) | 4.00E-63 |
| WRIS_3394 | GR303958 | 1 | 725 | OSJNBa0006A01.6 (O. sativa) | 2.00E-39 |
| WRIS_5896 | GR304946 | 1 | 348 | Os03g0388900 (O. sativa) | 9.00E-19 |
| **Unclear classification** | | | | | |
| WRIS_3410 | GR303962 | 1 | 720 | putative U4/U6 snRNP-associated 61 kDa protein (Oryza sativa) | 0.00E+00 |
| WRIS_287 | GR303734 | 1 | 667 | putative small nuclear ribonucleoprotein polypeptide E (O. sativa) | 2.00E-39 |
| WRIC_42 | GR302426 | 2 | 407 | putative B12D protein (O. sativa) | 1.00E-06 |
| WRIC_151 | GR302535 | 16 | 1588 | photosystem I assembly protein Ycf4 (T. aestivum) | 2.00E-93 |
| WRIC_306 | GR302690 | 2 | 1060 | hydrolase-like (O. sativa) | 1.00E-139 |
| WRIC_112 | GR302496 | 2 | 508 | VTC2, putative (O. sativa) | 2.00E-16 |
| WRIC_170 | GR302554 | 4 | 910 | triticain alpha (T. aestivum) | 1.00E-122 |
| WRIC_185 | GR302569 | 3 | 828 | Crystal Structure Of Presequence Protease (Arabidopsis Thaliana) | 3.00E-44 |
| WRIC_303 | GR302687 | 2 | 671 | putative genetic modifier (O. sativa) | 2.00E-30 |
| WRIC_225 | GR302609 | 2 | 599 | Metallothionein-like protein 1 (O. sativa) | 1.00E-10 |
| WRIC_226 | GR302610 | 3 | 537 | putative MTD2 (O. sativa) | 5.00E-18 |
| WRIC_379 | GR302763 | 6 | 556 | ORF100 (O. sativa) | 4.00E-19 |
| WRIC_382 | GR302766 | 18 | 514 | chloroplast hypothetical protein (Zea mays) | 3.00E-17 |
| WRIC_432 | GR302816 | 3 | 479 | sterility protein 1 (Phaseolus vulgaris) | 5.00E-15 |
| WRIC_383 | GR302767 | 3 | 731 | OsS5a (O. sativa) | 4.00E-22 |
| WRIC_458 | GR302842 | 4 | 737 | one helix protein (Deschampsia Antarctica) | 8.00E-42 |
| WRIC_498 | GR302882 | 2 | 451 | geranylgeranyl hydrogenase (T. aestivum) | 1.00E-20 |
| WRIC_514 | GR302898 | 2 | 999 | putative CEO protein (O. sativa) | 1.00E-131 |
| WRIS_100 | GR302974 | 1 | 478 | Protein H2A.7 (O. sativa) | 1.00E-33 |
| WRIS_1041 | GR302984 | 1 | 709 | DNA binding (Arabidopsis thaliana) | 1.00E-07 |
| WRIS_1042 | GR302985 | 1 | 430 | apospory-associated protein C-like (O. sativa) | 4.00E-12 |
| WRIS_2121 | GR303421 | 1 | 349 | biogenesis protein（H. vulgare） | 5.00E-14 |
| WRIS_1500 | GR303167 | 1 | 364 | one helix protein (Deschampsia Antarctica) | 6.00E-31 |
| WRIS_1502 | GR303169 | 1 | 438 | putative DnaJ domain containing protein (O. sativa) | 0.00E+00 |
| WRIS_1526 | GR303179 | 1 | 628 | putative small nuclear ribonucleoprotein polypeptide E (O. sativa) | 3.00E-38 |
| WRIS_1735 | GR303259 | 1 | 666 | putative CTV.22 (O. sativa) | 8.00E-71 |
| WRIS_201 | GR303382 | 1 | 573 | isochorismatase hydrolase-like protein (O. sativa) | 7.00E-54 |
| WRIS_2204 | GR303450 | 1 | 581 | SNF7 protein-like (O. sativa) | 5.00E-07 |
| WRIS_2464 | GR303566 | 1 | 760 | Uncharacterized Cys-rich domain (Medicago truncatula) | 1.00E-46 |
| WRIS_2618 | GR303624 | 1 | 519 | putative MTD2 (O. sativa) | 2.00E-26 |
| WRIS_2691 | GR303657 | 1 | 468 | putative SEC23 (O. sativa) | 2.00E-30 |
| WRIS_2881 | GR303736 | 1 | 684 | histone acetyltransferase HAT1 (T. aestivum) | 3.00E-61 |
| WRIS_3088 | GR303831 | 1 | 668 | Histone deacetylase family protein (O. sativa) | 5.00E-71 |
| WRIS_3098 | GR303836 | 1 | 654 | putative ariadne (O. sativa) | 1.00E-122 |
| WRIS_311 | GR303848 | 1 | 687 | putative CTP synthase (O. sativa) | 7.00E-25 |
| WRIS_3616 | GR304046 | 1 | 382 | LIM domain-containing protein, putative (O. sativa) | 7.00E-46 |
| WRIS_3642 | GR304060 | 1 | 600 | putative FKBP12 interacting protein (O. sativa) | 0.00E+00 |
| WRIS_4190 | GR304237 | 1 | 404 | Histidinol dehydrogenase, chloroplast precursor (O. sativa) | 3.00E-22 |
| WRIS_4578 | GR304388 | 1 | 671 | smr domain-containing protein -like (O. sativa) | 2.00E-82 |
| WRIS_4587 | GR304392 | 1 | 516 | putative nitrate-induced NOI protein (O. sativa) | 2.00E-37 |
| WRIS_4714 | GR304434 | 1 | 506 | putative nitrate-induced NOI protein (O. sativa) | 4.00E-23 |
| WRIS_4600 | GR304398 | 1 | 552 | GAMYB-binding protein（H. vulgare） | 1.00E-95 |
| WRIS_460 | GR304401 | 1 | 308 | putative z-protein (O. sativa) | 5.00E-29 |
| WRIS_472 | GR304445 | 1 | 735 | ankyrin repeat family protein (O. sativa) | 6.00E-52 |
| WRIS_4770 | GR304462 | 1 | 681 | Ureide permease 2 (O. sativa) | 1.00E-82 |
| WRIS_4779 | GR304466 | 1 | 300 | putative alpha-coat protein (O. sativa) | 7.00E-24 |
| WRIS_4785 | GR304471 | 1 | 376 | putative methyl-binding domain protein MBD111 (Z. mays) | 1.00E-58 |
| WRIS_4793 | GR304474 | 1 | 426 | 14-3-3-like protein B (O. sativa) | 8.00E-44 |
| WRIS_4838 | GR304497 | 1 | 725 | triticain alpha (T. aestivum) | 4.00E-66 |
| WRIS_4952 | GR304560 | 1 | 472 | cystatin Hv-CPI3（H. vulgare） | 6.00E-13 |
| WRIS_4987 | GR304575 | 1 | 338 | copper-translocating P-type ATPase family protein (O. sativa) | 7.00E-42 |
| WRIS_5031 | GR304597 | 1 | 563 | putative FH protein interacting protein FIP1 (O. sativa) | 6.00E-68 |
| WRIS_5034 | GR304600 | 1 | 537 | hesB-like domain-containing protein-like (O. sativa) | 7.00E-57 |
| WRIS_5056 | GR304609 | 1 | 544 | putative SF16 protein (O. sativa) | 4.00E-36 |
| WRIS_2208 | GR303452 | 1 | 593 | putative DAG protein (O. sativa) | 5.00E-07 |
| WRIS_278 | GR303700 | 1 | 410 | photosystem I assembly protein ycf3 (Lactuca sativa) | 1.00E-40 |
| WRIS_2954 | GR303767 | 1 | 739 | hypothetical protein PhapfoPp090 (Phalaenopsis Aphrodite) | 5.00E-47 |
| WRIS_5139 | GR304642 | 1 | 701 | biogenesis protein（H. vulgare） | 3.00E-59 |
| WRIS_5215 | GR304677 | 1 | 458 | spore coat protein -like (O. sativa) | 4.00E-16 |
| WRIS_5238 | GR304682 | 1 | 693 | putative DegP protease (O. sativa) | 9.00E-90 |
| WRIS_5304 | GR304707 | 1 | 730 | F-box domain containing protein (O. sativa) | 7.00E-21 |
| WRIS_5351 | GR304721 | 1 | 636 | Tat binding protein like protein (Brassica rapa) | 2.00E-93 |
| WRIS_5354 | GR304722 | 1 | 503 | Stem-specific protein TSJT1 (O. sativa) | 2.00E-29 |
| WRIS_5420 | GR304753 | 1 | 690 | DEAD/DEAH box helicase family protein (O. sativa) | 1.00E-103 |
| WRIS_5463 | GR304766 | 1 | 519 | putative MATE efflux family protein (O. sativa) | 1.00E-13 |
| WRIS_5567 | GR304809 | 1 | 350 | elenium-binding protein-like (O. sativa) | 3.00E-36 |
| WRIS_556 | GR304810 | 1 | 651 | putative syntaxin of plants 52 (O. sativa) | 4.00E-51 |
| WRIS_5645 | GR304842 | 1 | 586 | carboxyl-terminal proteinase-like (O. sativa) | 2.00E-74 |
| WRIS_5671 | GR304853 | 1 | 627 | putative B12D protein (O. sativa) | 3.00E-41 |
| WRIS_2023 | GR303384 | 1 | 727 | Endoplasmin homolog precursor (O. sativa) | 1.00E-110 |
| WRIS_3248 | GR303897 | 1 | 573 | NHL repeat-containing protein-like (O. sativa) | 1.00E-16 |
| WRIS_5848 | GR304922 | 1 | 714 | putative AdoMet synthase 3（H. vulgare） | 1.00E-108 |
| WRIS_5858 | GR304926 | 1 | 732 | I Chain I, Trypsin:bbi Complex | 4.00E-65 |
| WRIS_5869 | GR304931 | 1 | 691 | putative SPFH domain / Band 7 family (O. sativa) | 3.00E-86 |
| WRIS_5883 | GR304938 | 1 | 611 | kelch repeat-containing F-box family protein (O. sativa) | 2.00E-46 |
| WRIS_5902 | GR304951 | 1 | 165 | MATE efflux family protein, expressed (O. sativa) | 5.00E-14 |
| WRIS_591 | GR304957 | 1 | 679 | BRI1-KD interacting protein 103 (O. sativa) | 2.00E-22 |
| WRIS_646 | GR305003 | 1 | 328 | haloacid dehalogenase-like hydrolase-like protein (O. sativa) | 7.00E-06 |
| WRIS_709 | GR305025 | 1 | 600 | Erwinia chrysanthemi IndA protein homolog-like (O. sativa) | 8.00E-98 |
| WRIS_832 | GR305065 | 1 | 452 | homeobox gene (O. sativa) | 3.00E-40 |
| WRIS_936 | GR305102 | 1 | 511 | hesB-like domain-containing protein-like (O. sativa) | 4.00E-49 |
| WRIS_3325 | GR303930 | 1 | 759 | putative annexin (O. sativa) | 4.00E-07 |
| WRIS_3411 | GR303963 | 1 | 570 | apomixis-associated protein (T. aestivum) | 8.00E-41 |
| WRIS_3834 | GR304121 | 1 | 615 | Lipoxygenase 2.3, chloroplast precursor（H. vulgare） | 4.00E-72 |
| WRIS_4198 | GR304239 | 1 | 414 | putative oxidase-like (O. sativa) | 6.00E-52 |
| WRIS_4875 | GR304522 | 1 | 508 | PDI-like protein (Z. mays) | 3.00E-41 |
| WRIS_555 | GR304808 | 1 | 536 | major facilitator superfamily antiporter, putative (O. sativa) | 1.00E-35 |
| WRIS_1314 | GR303097 | 1 | 521 | putative NF-E2 inducible protein (O. sativa) | 1.00E-62 |
| WRIS_426 | GR304262 | 1 | 523 | GAMYB-binding protein（H. vulgare | 2.00E-84 |
| WRIS_535 | GR304725 | 1 | 704 | transposon protein, putative (O. sativa) | 1.00E-11 |
| WRIS_5812 | GR304908 | 1 | 442 | endoribonuclease E-like protein (O. sativa) | 3.00E-25 |
| WRIS_856 | GR305074 | 1 | 613 | C2 domain-containing protein-like (O. sativa) | 4.00E-72 |
| WRIC_30 | GR302414 | 2 | 573 | DnaJ domain containing protein (O. sativa) | 7.00E-21 |
| WRIS_3687 | GR304075 | 1 | 711 | VTC2, putative (O. sativa) | 1.00E-112 |
| WRIC_416 | GR302800 | 8 | 829 | ORF 62 | 7.00E-31 |
| WRIS_1105 | GR303011 | 1 | 649 | Sec13p (O. sativa) | 0.00E+00 |
| WRIS_3881 | GR304139 | 1 | 712 | putative G10 protein | 1.00E-82 |
| WRIS_3179 | GR303870 | 1 | 656 | metal-dependent hydrolase-like protein (O. sativa) | 1.00E-68 |
| WRIS_4035 | GR304176 | 1 | 576 | 68 kDa protein HP68 (T. aestivum) | 8.00E-34 |
