# Supplementary material for: Identification of expressed genes during compatible interaction between stripe rust (Puccinia striiformis) and wheat using a cDNA library
Source: BMC Genomics. 2009 Dec 8;10:586. doi: 10.1186/1471-2164-10-586 (PMC3087560; doi:10.1186/1471-2164-10-586)
Supplement: Additional file 2 — Unisequences from the compatible interaction between wheat and Puccinia striiformis f. sp. tritici showing significant similarities to fungi genes in the GenBank database. These data provide the original EST number and best hit. [file 1471-2164-10-586-S2.DOC]

**Additional file 2:** Unisequences from the compatible interaction between wheat and *Puccinia striiformis* f. sp. *tritici* showing significant similarities to fungi genes in the GenBank database.

| **User ID** | **Accession no.** | **Copy no.** | **Length** | **Similarity and species** | **E-value** |
| --- | --- | --- | --- | --- | --- |
| WRIC_1 | GR302385 | 3 | 734 | endopeptidase (*Cryptococcus neoformans*) | 3.00E-55 |
| WRIC_7 | GR302391 | 6 | 706 | 60S ribosomal protein(*C. neoformans*) | 2.00E-25 |
| WRIC_20 | GR302404 | 4 | 623 | 40S ribosomal protein (*Schizosaccharomyces pombe*) | 2.00E-36 |
| WRIC_34 | GR302418 | 5 | 738 | S15 (*Suberites domuncula*) | 2.00E-64 |
| WRIC_36 | GR302420 | 2 | 637 | ribosomal protein L13A (*Xanthophyllomyces dendrorhous*) | 6.00E-78 |
| WRIC_39 | GR302423 | 2 | 829 | 60s ribosomal protein l5-b (*C. neoformans*) | 1.00E-25 |
| WRIC_54 | GR302438 | 4 | 580 | 60s ribosomal protein l23 (*C. neoformans*) | 9.00E-62 |
| WRIC_59 | GR302443 | 4 | 678 | nucleoside-diphosphate kinase (*C. neoformans*) | 7.00E-57 |
| WRIC_73 | GR302457 | 3 | 753 | putative S-phase specific ribosomal protein cyc07 (*Lentinulaedodes*) | 8.00E-90 |
| WRIC_80 | GR302464 | 5 | 756 | ER to Golgi transport-related protein (*C. neoformans*) | 1.00E-45 |
| WRIC_84 | GR302468 | 10 | 749 | 40s ribosomal protein s5-1 (*C. neoformans*) | 4.00E-86 |
| WRIC_101 | GR302485 | 2 | 690 | 60s ribosomal protein l33-b (*C. neoformans*) | 6.00E-38 |
| WRIC_102 | GR302486 | 3 | 393 | phosphoprotein phosphatase (*C. neoformans*) | 1.00E-26 |
| WRIC_104 | GR302488 | 2 | 716 | hydroxyacylglutathione hydrolase (*C. neoformans*) | 6.00E-46 |
| WRIC_132 | GR302516 | 2 | 814 | glutamine-fructose-6-phosphate transaminase (*C. neoformans*) | 8.00E-52 |
| WRIC_144 | GR302528 | 2 | 695 | S15 (Suberites domuncula]) | 2.00E-64 |
| WRIC_175 | GR302559 | 11 | 607 | 60s ribosomal protein l33-b (*C. neoformans*) | 5.00E-38 |
| WRIC_177 | GR302561 | 26 | 568 | PRCDNA35 (*C. neoformans*) | 8.00E-20 |
| WRIC_181 | GR302565 | 13 | 949 | 40S ribosomal protein S9 (S7) (*Ustilago maydis*) | 6.00E-78 |
| WRIC_190 | GR302574 | 3 | 881 | 60S ribosomal protein L7 (Chaetomium globosum) | 4.00E-78 |
| WRIC_212 | GR302596 | 4 | 752 | 40S ribosomal protein S9 (*U. maydis*) | 4.00E-78 |
| WRIC_244 | GR302628 | 5 | 661 | ubiquitin-carboxy extension protein fusion (*C. neoformans*) | 5.00E-62 |
| WRIC_260 | GR302644 | 5 | 690 | 60S ribosomal protein L24 (*C. neoformans*) | 2.00E-25 |
| WRIC_280 | GR302664 | 5 | 970 | 40S ribosomal protein S3 (*Coccidioides immitis*) | 3.00E-97 |
| WRIC_302 | GR302686 | 6 | 818 | 60s ribosomal protein l11 (*C. neoformans*) | 1.00E-76 |
| WRIC_288 | GR302672 | 2 | 760 | C1-THF synthase (*Strongylocentrotus purpuratus*) | 6.00E-51 |
| WRIC_312 | GR302696 | 2 | 951 | rust transferred protein (Uromyces striatus) | 6.00E-27 |
| WRIC_322 | GR302706 | 3 | 978 | differentiation-related protein Infp (*Uromyces appendiculatus*) | 1.00E-14 |
| WRIC_324 | GR302708 | 2 | 756 | aldehyde dehydrogenase (Aspergillus terreus) | 2.00E-54 |
| WRIC_343 | GR302727 | 2 | 703 | GTP-binding nuclear protein RAN (*U. maydis*) | 1.00E-87 |
| WRIC_347 | GR302731 | 2 | 650 | AFR526Cp (Eremothecium gossypii) | 4.00E-30 |
| WRIC_348 | GR302732 | 10 | 785 | | PIG28 (Uromyces fabae) | 2.00E-53 |
| WRIC_365 | GR302749 | 2 | 633 | alcohol dehydrogenase (Puccinia triticina) | 6.00E-70 |
| WRIC_368 | GR302752 | 6 | 587 | ribosomal protein L36 (TRP36) (Gibberella zeae) | 2.00E-21 |
| WRIC_375 | GR302759 | 10 | 700 | ribosomal protein S19 (*C. neoformans*) | 2.00E-57 |
| WRIC_381 | GR302765 | 2 | 661 | 60s ribosomal protein l20 (yl17) (*C. neoformans*) | 2.00E-71 |
| WRIC_387 | GR302771 | 2 | 707 | L-malate dehydrogenase (*C. neoformans*) | 4.00E-48 |
| WRIC_401 | GR302785 | 2 | 471 | protein transporter (*C. neoformans*) | 3.00E-19 |
| WRIC_406 | GR302790 | 2 | 750 | chitin deacetylase (Flammulina velutipes) | 3.00E-25 |
| WRIC_413 | GR302797 | 2 | 546 | cytochrome P450 (Rhodotorula sp) | 6.00E-16 |
| WRIC_420 | GR302804 | 5 | 640 | 40s ribosomal protein (Aspergillus fumigatus) | 3.00E-39 |
| WRIC_439 | GR302823 | 4 | 773 | structural constituent of ribosome (*C. neoformans*) | 5.00E-96 |
| WRIC_460 | GR302844 | 11 | 585 | L41 ribosomal protein (*C. neoformans*) | 6.00E-53 |
| WRIC_480 | GR302864 | 3 | 661 | glutathione S-transferase (*C. neoformans*) | 3.00E-41 |
| WRIC_495 | GR302879 | 7 | 1333 | elongation factor (*Puccinia graminis*) | 0 |
| WRIC_455 | GR302839 | 8 | 1182 | ATP:ADP antiporter (*C. neoformans*) | 1.00E-126 |
| WRIC_540 | GR302924 | 2 | 718 | alpha tubulin (*C. neoformans*) | 1.00E-100 |
| WRIC_544 | GR302928 | 2 | 394 | elongation factor 2 (Neurospora crassa) | 1.00E-26 |
| WRIC_547 | GR302931 | 5 | 676 | large (60S) ribosomal subunit (*Saccharomyces cerevisiae*) | 5.00E-15 |
| WRIC_551 | GR302935 | 2 | 850 | elongation factor 3 (*C. neoformans*) | 8.00E-58 |
| WRIC_552 | GR302936 | 5 | 682 | cytosolic small ribosomal subunit protein (*Aspergillus fumigatus*) | 2.00E-39 |
| WRIC_555 | GR302939 | 9 | 880 | ribosomal L10 protein (*C. neoformans*) | 6.00E-93 |
| WRIC_574 | GR302958 | 2 | 881 | Beta-hexosaminidase precursor (*C. neoformans*) | 2.00E-62 |
| WRIC_575 | GR302959 | 2 | 595 | ADR345Cp (Eremothecium gossypii) | 5.00E-18 |
| WRIC_576 | GR302960 | 3 | 661 | ribosomal protein S11 (*C. neoformans*) | 1.00E-65 |
| WRIC_580 | GR302964 | 2 | 743 | disulfide-isomerase precursor (*C. neoformans*) | 3.00E-17 |
| WRIS_1017 | GR302976 | 1 | 484 | oxidoreductase (Streptomyces avermitilis) | 5.00E-28 |
| WRIS_1032 | GR302982 | 1 | 474 | cytochrome P450 (Rhodotorula sp) | 1.00E-16 |
| WRIS_1115 | GR303016 | 1 | 614 | ribosomal protein (*C. neoformans*) | 3.00E-30 |
| WRIS_1157 | GR303034 | 1 | 703 | transketolase (*C. neoformans*) | 7.00E-85 |
| WRIS_1197 | GR303052 | 1 | 663 | malate dehydrogenase (*C. neoformans*) | 5.00E-55 |
| WRIS_1248 | GR303069 | 1 | 399 | imidazoleglycerol-phosphate dehydratase (*C. neoformans*) | 1.00E-10 |
| WRIS_1233 | GR303063 | 1 | 368 | mRNA processing-related protein (*C. neoformans*) | 5.00E-27 |
| WRIS_1277 | GR303084 | 1 | 606 | phytase (*C. neoformans*) | 3.00E-38 |
| WRIS_1347 | GR303111 | 1 | 684 | endoplasmic reticulum protein (*C. neoformans*) | 4.00E-36 |
| WRIS_1374 | GR303121 | 1 | 499 | 60s ribosomal protein (*C. neoformans*) | 1.00E-56 |
| WRIS_1608 | GR303211 | 1 | 604 | THI2p (*Uromyces viciae-fabae*) | 8.00E-75 |
| WRIS_1741 | GR303261 | 1 | 666 | CAP1 (*Cryptococcus gattii*) | 1.00E-36 |
| WRIS_1773 | GR303275 | 1 | 739 | actin (*Puccinia graminis*) | 1.00E-114 |
| WRIS_1781 | GR303282 | 1 | 643 | actin (*P. graminis*) | 1.00E-51 |
| WRIS_182 | GR303306 | 1 | 479 | tricarboxylate carrier (*C. neoformans*) | 2.00E-39 |
| WRIS_2275 | GR303481 | 1 | 337 | protein phosphatase 2A (*U. maydis*) | 7.00E-58 |
| WRIS_2282 | GR303483 | 1 | 601 | heavy metal ion transporter (*A. fumigatus*) | 6.00E-14 |
| WRIS_229 | GR303490 | 1 | 454 | glucose-repressible gene protein-related protein (*A. fumigatus*) | 2.00E-14 |
| WRIS_2302 | GR303493 | 1 | 429 | hesp-767 (*Melampsora lini*) | 1.00E-29 |
| WRIS_2093 | GR303411 | 1 | 733 | N-hydroxyarylamine O-acetyltransferase (Streptomyces avermitilis) | 1.00E-12 |
| WRIS_2222 | GR303459 | 1 | 590 | phosphatidylinositol kinase (Schizosaccharomyces pombe) | 2.00E-54 |
| WRIS_2259 | GR303474 | 1 | 579 | prenyltransferase (*A. fumigatus*) | 5.00E-31 |
| WRIS_2031 | GR303386 | 1 | 751 | chitinase (*Puccinia triticina*) | 2.00E-25 |
| WRIS_2324 | GR303503 | 1 | 696 | cytoplasm protein (*C. neoformans*) | 9.00E-63 |
| WRIS_2369 | GR303522 | 1 | 685 | Ser/Thr protein kinase (*C. neoformans*) | 8.00E-14 |
| WRIS_2449 | GR303558 | 1 | 727 | Vacuolar ATP synthase subunit | 4.00E-08 |
| WRIS_2496 | GR303579 | 1 | 505 | t-complex protein 1, eta subunit (*C. neoformans*) | 2.00E-34 |
| WRIS_2546 | GR303599 | 1 | 611 | protein arginine n-methyltransferase (*C. neoformans*) | 8.00E-93 |
| WRIS_2607 | GR303621 | 1 | 718 | riboflavin-aldehyde forming enzyme (*Agaricus bisporus*) | 1.00E-14 |
| WRIS_2632 | GR303628 | 1 | 512 | putative ER dolichol phosphate mannose synthase (*Candida albicans*) | 2.00E-52 |
| WRIS_265 | GR303636 | 1 | 313 | heat-shock protein 90 (*Cryptococcus bacillisporus*) | 4.00E-20 |
| WRIS_2675 | GR303648 | 1 | 745 | 20S proteasome subunit | 2.00E-97 |
| WRIS_2512 | GR303588 | 1 | 691 | ATP-citrate synthase subunit 1 (*Aspergillus terreus*) | 1.00E-34 |
| WRIS_2350 | GR303515 | 1 | 725 | structural constituent of ribosome (*C. neoformans*) | 1.00E-44 |
| WRIS_2702 | GR303661 | 1 | 708 | tyrosine-tRNA ligase (*C. neoformans*) | 4.00E-38 |
| WRIS_2704 | GR303663 | 1 | 763 | nucleolus protein (*C. neoformans*) | 1.00E-08 |
| WRIS_294 | GR303765 | 1 | 585 | (60S) ribosomal subunit (*Saccharomyces cerevisiae*) | 4.00E-15 |
| WRIS_2959 | GR303771 | 1 | 728 | Glyceraldehyde-3-phosphate dehydrogenase (*Cryptococcus curvatus*) | 2.00E-97 |
| WRIS_298 | GR303786 | 1 | 693 | hesp-735 (*Melampsora lini*) | 4.00E-07 |
| WRIS_3000 | GR303791 | 1 | 512 | 2-isopropylmalate synthase (*Agrobacterium tumefaciens*) | 3.00E-23 |
| WRIS_3006 | GR303794 | 1 | 324 | DNA unwinding-related protein (*C. neoformans*) | 2.00E-08 |
| WRIS_3037 | GR303808 | 1 | 406 | aspartyl proteinase (*Trichoderma asperellum*) | 1.00E-25 |
| WRIS_3063 | GR303820 | 1 | 487 | alpha-1,3-glucan synthase (*C. neoformans*) | 2.00E-07 |
| WRIS_3072 | GR303824 | 1 | 427 | sterol-binding protein (*C. neoformans*) | 1.00E-14 |
| WRIS_3117 | GR303845 | 1 | 226 | phosphoglucomutase (*C. neoformans*) | 3.00E-18 |
| WRIS_316 | GR303865 | 1 | 619 | PIG1 *(Uromyces fabae)* | 0 |
| WRIS_3174 | GR303867 | 1 | 503 | cleft lip and palate associated transmembrane protein (*C. neoformans*) | 2.00E-33 |
| WRIS_4144 | GR304218 | 1 | 376 | aspartate-tRNA ligase (*C. neoformans*) | 3.00E-15 |
| WRIS_3205 | GR303883 | 1 | 668 | Machado-Joseph disease protein 1 | 2.00E-20 |
| WRIS_3241 | GR303893 | 1 | 758 | succinate dehydrogenase cytochrome b560 subunit (*Coprinopsiscinerea*) | 6.00E-39 |
| WRIS_3246 | GR303896 | 1 | 695 | hypothetical class II chitin synthase (*P. graminis*) | 9.00E-29 |
| WRIS_3292 | GR303916 | 1 | 604 | pyruvate dehydrogenase e1 component alpha subunit (*C. neoformans*) | 1.00E-90 |
| WRIS_3415 | GR303965 | 1 | 418 | fructose-bisphosphate aldolase (*C. neoformans*) | 2.00E-15 |
| WRIS_3511 | GR304003 | 1 | 790 | chitinase (*Puccinia triticina*) | 0 |
| WRIS_3524 | GR304012 | 1 | 467 | small nuclear ribonucleo protein (*C. neoformans*) | 6.00E-37 |
| WRIS_3567 | GR304030 | 1 | 666 | 40S ribosomal protein S9 *(U. maydis)* | 1.00E-79 |
| WRIS_3617 | GR304047 | 1 | 597 | vacuolar multi-drug resistance ABC transporter (*Candida albicans*) | 2.00E-27 |
| WRIS_3631 | GR304056 | 1 | 481 | hydrogen-transporting ATP synthase (*C. neoformans*) | 1.00E-20 |
| WRIS_3640 | GR304058 | 1 | 517 | DNA-directed RNA polymerase II protein (*S. pombe*) | 8.00E-20 |
| WRIS_3665 | GR304070 | 1 | 431 | G-protein beta subunit GPB1 (*Filobasidiella neoformans*) | 2.00E-63 |
| WRIS_3702 | GR304083 | 1 | 309 | glutamine synthetase (*Paxillus involutus*) | 1.00E-09 |
| WRIS_3740 | GR304095 | 1 | 651 | phosphotyrosyl phosphatase activator (*C. neoformans*) | 5.00E-51 |
| WRIS_3865 | GR304134 | 1 | 417 | mitochondrion protein (*C. neoformans*) | 2.00E-31 |
| WRIS_3941 | GR304154 | 1 | 445 | protein-vacuolar targeting protein (*C. neoformans*) | 1.00E-17 |
| WRIS_3950 | GR304158 | 1 | 598 | ntra-Golgi transport-related protein (*C. neoformans*) | 3.00E-29 |
| WRIS_4003 | GR304167 | 1 | 699 | Ras2 (*C. neoformans*) | 1.00E-74 |
| WRIS_3158 | GR303861 | 1 | 556 | glutamine synthetase (*Hebeloma cylindrosporum*) | 3.00E-57 |
| WRIS_4154 | GR304223 | 1 | 661 | exosome complex exonuclease (*C. neoformans*) | 3.00E-55 |
| WRIS_4212 | GR304243 | 1 | 500 | mitochondrial inner membrane protein (*C. neoformans*) | 0 |
| WRIS_4309 | GR304275 | 1 | 550 | DNA primase catalytic subunit (*Coprinopsis cinerea*) | 0 |
| WRIS_4318 | GR304280 | 1 | 524 | HSS1 (*P. graminis*) | 0 |
| WRIS_4357 | GR304295 | 1 | 593 | 60S RIBOSOMAL PROTEIN L15 (*Neurospora crassa*) | 0 |
| WRIS_4468 | GR304336 | 1 | 658 | glyoxal oxidase precursor (*C. neoformans*) | 0 |
| WRIS_4478 | GR304340 | 1 | 572 | 3-deoxy-7-phosphoheptulonate synthase (*Paxillus involutus*) | 4.00E-29 |
| WRIS_4517 | GR304357 | 1 | 347 | ATP-binding cassette transporter ABC2 (*Venturia inaequalis*) | 2.00E-30 |
| WRIS_4530 | GR304364 | 1 | 658 | deacetylase (*C. neoformans*) | 1.00E-23 |
| WRIS_4768 | GR304461 | 1 | 409 | 3-ketoacyl-CoA thiolase (*Aspergillus terreus*) | 1.00E-17 |
| WRIS_4812 | GR304485 | 1 | 412 | Cro r II (*Cronartium ribicola*) | 7.00E-16 |
| WRIS_4864 | GR304515 | 1 | 647 | cytoplasm protein (*C. neoformans*) | 6.00E-73 |
| WRIS_4887 | GR304526 | 1 | 623 | cytochrome P450 (*Phanerochaete chrysosporium*) | 2.00E-36 |
| WRIS_4926 | GR304552 | 1 | 669 | MGC81460 protein (*Xenopus laevis*) | 5.00E-12 |
| WRIS_5090 | GR304620 | 1 | 657 | putative regulator of purine operon, putative translationinitiation inhibitor (*Bacillus licheniformis*) | 2.00E-31 |
| WRIS_5102 | GR304626 | 1 | 631 | fructose-bisphosphatase (*C. neoformans*) | 5.00E-70 |
| WRIS_5105 | GR304627 | 1 | 362 | pre-mRNA splicing factor (*C. neoformans*) | 1.00E-28 |
| WRIS_5113 | GR304632 | 1 | 431 | cytochrome c oxidase family protein (*Aspergillus fumigatus*) | 2.00E-10 |
| WRIS_5190 | GR304665 | 1 | 265 | glutamine synthetase (*Amanita muscaria*) | 8.00E-36 |
| WRIS_5280 | GR304697 | 1 | 458 | 40S ribosomal protein S8 (*Schizophyllum commune*) | 8.00E-49 |
| WRIS_5378 | GR304735 | 1 | 806 | ARF small monomeric GTPase (*C. neoformans*) | 1.00E-72 |
| WRIS_5408 | GR304747 | 1 | 755 | 40S ribosomal protein S23-like protein (*Magnaporthe grisea*) | 5.00E-68 |
| WRIS_5426 | GR304754 | 1 | 667 | ribosomal large subunit biogenesis-related protein (*C. neoformans*) | 3.00E-51 |
| WRIS_5646 | GR304843 | 1 | 693 | nucleus protein (*C. neoformans*) | 8.00E-19 |
| WRIS_573 | GR304873 | 1 | 705 | aerobic respiration-related protein (*C. neoformans*) | 2.00E-24 |
| WRIS_574 | GR304877 | 1 | 707 | alcohol dehydrogenase (*P. triticina*) | 7.00E-82 |
| WRIS_5778 | GR304891 | 1 | 409 | DNA binding protein (*C. neoformans*) | 3.00E-39 |
| WRIS_5782 | GR304893 | 1 | 764 | DNA-binding protein (*C. neoformans*) | 1.00E-23 |
| WRIS_5792 | GR304898 | 1 | 600 | ribosomal protein S19 (*C. neoformans*) | 1.00E-57 |
| WRIS_5973 | GR304976 | 1 | 721 | ribosomal protein L4 (*C. neoformans*) | 2.00E-83 |
| WRIS_5978 | GR304978 | 1 | 412 | protein kinase Fuz7 (*U. maydis*) | 3.00E-17 |
| WRIS_5984 | GR304982 | 1 | 539 | LSM (like-Sm) domain-containing protein (*Dictyostelium discoideum*) | 7.00E-25 |
| WRIS_706 | GR305023 | 1 | 531 | alpha-galactosidase (*Streptomyces avermitilis*) | 0 |
| WRIS_753 | GR305034 | 1 | 540 | myosin, light chain 2, 20 kDa (*C. neoformans*) | 4.00E-32 |
| WRIS_773 | GR305040 | 1 | 658 | citrate synthase, mitochondrial precursor (*Aspergillus terreus*) | 4.00E-30 |
| WRIS_811 | GR305057 | 1 | 509 | mRNA processing-related protein (*C. neoformans*) | 4.00E-27 |
| WRIS_827 | GR305062 | 1 | 661 | mitochondrial import inner membrane translocase subunit (*C. neoformans*) | 6.00E-39 |
| WRIS_852 | GR305072 | 1 | 634 | similar to glutathione reductase (*Strongylocentrotus purpuratus*) | 3.00E-67 |
| WRIS_924 | GR305099 | 1 | 573 | homoserine kinase (*C. neoformans*) | 3.00E-42 |
| WRIS_928 | GR305100 | 1 | 424 | 60S ribosomal protein L38 (*Argas monolakensi*) | 3.00E-16 |
| WRIS_962 | GR305112 | 1 | 651 | vesicle-mediated transport-related protein (*C. neoformans*) | 2.00E-19 |
| WRIC_12 | GR302396 | 8 | 970 | hypothetical protein (*U. maydis*) | 1.00E-109 |
| WRIC_18 | GR302402 | 3 | 774 | hypothetical protein (*C. neoformans*) | 1.00E-14 |
| WRIC_26 | GR302410 | 3 | 886 | hypothetical protein (*U. maydis*) | 3.00E-86 |
| WRIC_29 | GR302413 | 3 | 627 | hypothetical protein (*U. maydis*) | 5.00E-50 |
| WRIC_32 | GR302416 | 7 | 597 | unnamed protein product (*Candida glabrata*) | 6.00E-58 |
| WRIC_40 | GR302424 | 30 | 365 | unnamed protein product (*Kluyveromyces lactis*) | 2.00E-11 |
| WRIC_53 | GR302437 | 2 | 697 | hypothetical protein (*U. maydis*) | 1.00E-45 |
| WRIC_61 | GR302445 | 2 | 565 | hypothetical protein (*U. maydis*) | 1.00E-08 |
| WRIC_67 | GR302451 | 2 | 572 | hypothetical protein (*U. maydis*) | 8.00E-36 |
| WRIC_75 | GR302459 | 23 | 1730 | hypothetical protein (*Candida albicans*) | 7.00E-27 |
| WRIC_79 | GR302463 | 3 | 1251 | hypothetical protein (*U. maydis*) | 1.00E-134 |
| WRIC_97 | GR302481 | 20 | 792 | hypothetical protein (*U. maydis*) | 4.00E-42 |
| WRIC_99 | GR302483 | 2 | 620 | hypothetical protein (*C. neoformans*) | 4.00E-15 |
| WRIC_107 | GR302491 | 4 | 740 | hypothetical protein (*Gibberella zeae*) | 3.00E-13 |
| WRIC_108 | GR302492 | 3 | 837 | unnamed protein product (*Aspergillus oryzae*) | 5.00E-49 |
| WRIC_125 | GR302509 | 2 | 736 | unnamed protein product (*Candida glabrata*) | 1.00E-47 |
| WRIC_136 | GR302520 | 5 | 743 | hypothetical protein (*C. neoformans*) | 1.00E-34 |
| WRIC_140 | GR302524 | 2 | 786 | conserved hypothetical protein (*Aspergillus terreus*) | 2.00E-20 |
| WRIC_147 | GR302531 | 3 | 816 | hypothetical protein (*C. neoformans*) | 1.00E-06 |
| WRIC_154 | GR302538 | 7 | 735 | hypothetical protein (*Magnaporthe grisea*) | 5.00E-68 |
| WRIC_162 | GR302546 | 15 | 680 | hypothetical protein (*C. neoformans*) | 2.00E-48 |
| WRIC_166 | GR302550 | 4 | 739 | unnamed protein product (*Kluyveromyces lactis*) | 4.00E-13 |
| WRIC_173 | GR302557 | 3 | 682 | hypothetical protein (*U. maydis*) | 4.00E-56 |
| WRIC_183 | GR302567 | 4 | 747 | hypothetical protein (*U. maydis*) | 7.00E-51 |
| WRIC_188 | GR302572 | 2 | 713 | hypothetical protein (*Phaeosphaeria nodorum*) | 1.00E-10 |
| WRIC_193 | GR302577 | 2 | 741 | hypothetical protein (*C. neoformans*) | 6.00E-13 |
| WRIC_201 | GR302585 | 2 | 692 | hypothetical protein (*U. maydis*) | 1.00E-44 |
| WRIC_202 | GR302586 | 75 | 476 | unnamed protein product (*Kluyveromyces lactis*) | 2.00E-33 |
| WRIC_206 | GR302590 | 8 | 479 | hypothetical protein (*U. maydis*) | 2.00E-25 |
| WRIC_223 | GR302607 | 24 | 325 | unnamed protein product (K. lactis) | 1.00E-11 |
| WRIC_228 | GR302612 | 3 | 622 | hypothetical protein (*Schizosaccharomyces pombe*) | 2.00E-32 |
| WRIC_231 | GR302615 | 2 | 717 | hypothetical protein (*U. maydis*) | 1.00E-52 |
| WRIC_234 | GR302618 | 2 | 655 | hypothetical protein (*Phaeosphaeria nodorum*) | 2.00E-36 |
| WRIC_241 | GR302625 | 3 | 472 | hypothetical protein (*Coccidioides immitis*) | 3.00E-36 |
| WRIC_246 | GR302630 | 3 | 739 | hypothetical protein (*M. grisea*) | 5.00E-68 |
| WRIC_262 | GR302646 | 3 | 765 | hypothetical protein (*U. maydis*) | 8.00E-18 |
| WRIC_264 | GR302648 | 3 | 1046 | hypothetical protein (*U. maydis*) | 1.00E-142 |
| WRIC_278 | GR302662 | 8 | 734 | hypothetical protein (*C. neoformans*) | 7.00E-72 |
| WRIC_279 | GR302663 | 6 | 750 | hypothetical protein (*P. nodorum*) | 3.00E-36 |
| WRIC_283 | GR302667 | 2 | 646 | hypothetical protein (*U. maydis*) | 3.00E-39 |
| WRIC_286 | GR302670 | 7 | 985 | hypothetical protein (*Tetrahymena thermophila*) | 2.00E-53 |
| WRIC_289 | GR302673 | 2 | 828 | hypothetical protein (*U. maydis*) | 4.00E-19 |
| WRIC_291 | GR302675 | 7 | 775 | hypothetical protein (*U. maydis*) | 8.00E-21 |
| WRIC_304 | GR302688 | 2 | 663 | hypothetical protein (*U. maydis*) | 3.00E-27 |
| WRIC_319 | GR302703 | 4 | 412 | hypothetical protein (Neurospora crassa) | 5.00E-22 |
| WRIC_325 | GR302709 | 2 | 1152 | hypothetical protein (*Aspergillus nidulans*) | 1.00E-100 |
| WRIC_327 | GR302711 | 2 | 1031 | hypothetical protein (*U. maydis*) | 5.00E-58 |
| WRIC_328 | GR302712 | 2 | 706 | hypothetical protein (*U. maydis*) | 5.00E-30 |
| WRIC_331 | GR302715 | 20 | 571 | hypothetical protein (*U. maydis*) | 2.00E-15 |
| WRIC_334 | GR302718 | 4 | 731 | unnamed protein product (Aspergillus oryzae) | 6.00E-34 |
| WRIC_344 | GR302728 | 6 | 873 | hypothetical protein (*C. neoformans*) | 2.00E-45 |
| WRIC_352 | GR302736 | 2 | 540 | hypothetical protein (*Neurospora crassa*) | 3.00E-14 |
| WRIC_355 | GR302739 | 6 | 1097 | hypothetical protein (*U. maydis*) | 2.00E-90 |
| WRIC_356 | GR302740 | 89 | 1395 | hypothetical protein (*Clostridium tetani*) | 1.00E-46 |
| WRIC_366 | GR302750 | 3 | 573 | hypothetical protein (*C. neoformans*) | 2.00E-08 |
| WRIC_370 | GR302754 | 3 | 513 | predicted protein (*Phaeosphaeria nodorum*) | 2.00E-16 |
| WRIC_372 | GR302756 | 2 | 586 | hypothetical protein (*P nodorum*) | 6.00E-48 |
| WRIC_377 | GR302761 | 9 | 594 | hypothetical protein (*Coccidioides immitis*) | 1.00E-25 |
| WRIC_389 | GR302773 | 3 | 1150 | predicted protein (*P. nodorum*) | 3.00E-20 |
| WRIC_390 | GR302774 | 2 | 636 | hypothetical protein (*Aspergillus terreus*) | 2.00E-69 |
| WRIC_391 | GR302775 | 2 | 747 | hypothetical protein (*U. maydis*) | 1.00E-113 |
| WRIC_393 | GR302777 | 7 | 564 | unnamed protein product (K. lactis) | 4.00E-32 |
| WRIC_399 | GR302783 | 3 | 716 | hypothetical protein (*C. neoformans*) | 1.00E-09 |
| WRIC_445 | GR302829 | 2 | 830 | hypothetical protein (*C. neoformans*) | 1.00E-83 |
| WRIC_444 | GR302828 | 14 | 613 | PRCDNA38 (*C. neoformans*) | 5.00E-27 |
| WRIC_447 | GR302831 | 14 | 406 | unnamed protein product (*K. lactis*) | 5.00E-09 |
| WRIC_459 | GR302843 | 9 | 635 | hypothetical protein (S. pombe) | 5.00E-37 |
| WRIC_488 | GR302872 | 5 | 694 | hypothetical protein (*Neurospora crassa*) | 3.00E-38 |
| WRIC_496 | GR302880 | 2 | 627 | hypothetical protein (*U. maydis*) | 1.00E-19 |
| WRIC_502 | GR302886 | 2 | 642 | hypothetical protein (*U. maydis*) | 5.00E-63 |
| WRIC_504 | GR302888 | 2 | 559 | PRCDNA38 (*C. neoformans*) | 4.00E-27 |
| WRIC_517 | GR302901 | 2 | 1051 | hypothetical protein (*U. maydis*) | 3.00E-52 |
| WRIC_532 | GR302916 | 5 | 882 | hypothetical protein (*U. maydis*) | 2.00E-77 |
| WRIC_539 | GR302923 | 2 | 869 | hypothetical protein (*U. maydis*) | 3.00E-70 |
| WRIC_542 | GR302926 | 15 | 755 | unnamed protein product (*K. lactis*) | 8.00E-23 |
| WRIC_549 | GR302933 | 4 | 570 | hypothetical protein (*U. maydis*) | 3.00E-46 |
| WRIC_554 | GR302938 | 2 | 454 | hypothetical protein (*U. maydis*) | 8.00E-07 |
| WRIC_556 | GR302940 | 2 | 725 | hypothetical protein (*C. neoformans*) | 2.00E-38 |
| WRIC_567 | GR302951 | 4 | 741 | PRCDNA87.(*C. neoformans*) | 4.00E-37 |
| WRIC_570 | GR302954 | 2 | 726 | hypothetical protein (*C. neoformans*) | 1.00E-51 |
| WRIC_577 | GR302961 | 2 | 830 | hypothetical protein (*C. neoformans*) | 4.00E-07 |
| WRIC_582 | GR302966 | 2 | 860 | hypothetical protein (*U. maydis*) | 1.00E-70 |
| WRIC_583 | GR302967 | 3 | 615 | hypothetical protein (*C. neoformans*) | 3.00E-12 |
| WRIS_1003 | GR302970 | 1 | 689 | hypothetical protein (*U. maydis*) | 2.00E-20 |
| WRIS_1025 | GR302979 | 1 | 682 | hypothetical protein (*Neurospora crassa*) | 2.00E-43 |
| WRIS_1072 | GR303000 | 1 | 665 | hypothetical protein (*U. maydis*) | 1.00E-62 |
| WRIS_1076 | GR303001 | 1 | 644 | hypothetical protein (*C. neoformans*) | 2.00E-39 |
| WRIS_1108 | GR303012 | 1 | 612 | hypothetical protein (*U. maydis*) | 5.00E-22 |
| WRIS_1109 | GR303013 | 1 | 657 | hypothetical protein (*Neurospora crassa*) | 4.00E-44 |
| WRIS_1118 | GR303018 | 1 | 590 | hypothetical protein (*C. neoformans*) | 6.00E-07 |
| WRIS_1166 | GR303038 | 1 | 671 | hypothetical protein (*U. maydis*) | 1.00E-87 |
| WRIS_1256 | GR303073 | 1 | 410 | hypothetical protein (*U. maydis*) | 3.00E-10 |
| WRIS_1268 | GR303078 | 1 | 516 | hypothetical protein (*C. neoformans*) | 4.00E-64 |
| WRIS_1291 | GR303088 | 1 | 653 | hypothetical protein (*C. neoformans*) | 2.00E-15 |
| WRIS_1308 | GR303094 | 1 | 480 | hypothetical protein (*C. neoformans*) | 2.00E-06 |
| WRIS_1313 | GR303096 | 1 | 577 | hypothetical protein (*Debaryomyces hansenii*) | 6.00E-66 |
| WRIS_1324 | GR303101 | 1 | 643 | hypothetical protein (*U. maydis*) | 2.00E-25 |
| WRIS_1349 | GR303112 | 1 | 613 | hypothetical protein (*U. maydis*) | 2.00E-37 |
| WRIS_1358 | GR303113 | 1 | 603 | hypothetical protein (*U. maydis*) | 4.00E-21 |
| WRIS_1360 | GR303114 | 1 | 651 | hypothetical protein (*C. neoformans*) | 2.00E-67 |
| WRIS_1369 | GR303118 | 1 | 628 | hypothetical protein (*Aspergillus fumigatus*) | 2.00E-48 |
| WRIS_137 | GR303123 | 1 | 598 | hypothetical protein (*U. maydis*) | 2.00E-31 |
| WRIS_1584 | GR303201 | 1 | 492 | hypothetical protein *(P nodorum)* | 6.00E-09 |
| WRIS_1596 | GR303205 | 1 | 690 | hypothetical protein (*U. maydis*) | 1.00E-53 |
| WRIS_159 | GR303207 | 1 | 489 | predicted protein (*Coccidioides immitis*) | 1.00E-26 |
| WRIS_1614 | GR303214 | 1 | 527 | hypothetical protein (*U. maydis*) | 2.00E-37 |
| WRIS_1621 | GR303219 | 1 | 436 | hypothetical protein (*U. maydis*) | 3.00E-52 |
| WRIS_1710 | GR303249 | 1 | 455 | hypothetical protein (*C. neoformans*) | 2.00E-08 |
| WRIS_1714 | GR303251 | 1 | 657 | hypothetical protein (*U. maydis*) | 2.00E-16 |
| WRIS_1723 | GR303255 | 1 | 507 | hypothetical protein (*U. maydis*) | 3.00E-33 |
| WRIS_1750 | GR303267 | 1 | 705 | hypothetical protein (*C. neoformans*) | 1.00E-19 |
| WRIS_1777 | GR303278 | 1 | 705 | hypothetical protein (*Aspergillus nidulans*) | 7.00E-39 |
| WRIS_1792 | GR303290 | 1 | 606 | hypothetical protein (*U. maydis*) | 1.00E-27 |
| WRIS_1784 | GR303285 | 1 | 645 | hypothetical protein (*C. neoformans*) | 5.00E-70 |
| WRIS_1823 | GR303302 | 1 | 717 | hypothetical protein (*C. neoformans*) | 2.00E-50 |
| WRIS_184 | GR303314 | 1 | 670 | hypothetical protein (*U. maydis*) | 3.00E-69 |
| WRIS_1889 | GR303333 | 1 | 525 | hypothetical protein (*C. neoformans*) | 4.00E-07 |
| WRIS_1892 | GR303335 | 1 | 649 | hypothetical protein (*C. neoformans*) | 4.00E-26 |
| WRIS_1894 | GR303336 | 1 | 696 | hypothetical protein (*U. maydis*) | 9.00E-29 |
| WRIS_1928 | GR303351 | 1 | 784 | hypothetical protein (*C. neoformans*) | 8.00E-56 |
| WRIS_1978 | GR303365 | 1 | 700 | hypothetical protein (*U. maydis*) | 1.00E-69 |
| WRIS_1968 | GR303362 | 1 | 748 | hypothetical protein (*Neurospora crassa*) | 5.00E-81 |
| WRIS_1996 | GR303372 | 1 | 281 | hypothetical protein (*U. maydis*) | 6.00E-18 |
| WRIS_2063 | GR303398 | 1 | 558 | hypothetical protein *(P nodorum)* | 4.00E-35 |
| WRIS_2081 | GR303405 | 1 | 707 | hypothetical protein (*U. maydis*) | 9.00E-58 |
| WRIS_2113 | GR303417 | 1 | 445 | hypothetical protein (*C. neoformans*) | 1.00E-24 |
| WRIS_2114 | GR303418 | 1 | 642 | hypothetical protein (*U. maydis*) | 1.00E-53 |
| WRIS_2186 | GR303445 | 1 | 698 | hypothetical protein (*U. maydis*) | 3.00E-21 |
| WRIS_2187 | GR303446 | 1 | 672 | hypothetical protein (*C. neoformans*) | 7.00E-39 |
| WRIS_2216 | GR303456 | 1 | 332 | hypothetical protein (*C. neoformans*) | 4.00E-19 |
| WRIS_2228 | GR303463 | 1 | 707 | hypothetical protein (*U. maydis*) | 8.00E-28 |
| WRIS_2317 | GR303499 | 1 | 723 | hypothetical protein (*U. maydis*) | 7.00E-16 |
| WRIS_2400 | GR303536 | 1 | 792 | hypothetical protein (*C. neoformans*) | 3.00E-18 |
| WRIS_2501 | GR303582 | 1 | 741 | hypothetical protein (*U. maydis*) | 4.00E-19 |
| WRIS_2582 | GR303612 | 1 | 646 | predicted protein (*P. nodorum*) | 3.00E-07 |
| WRIS_2599 | GR303617 | 1 | 567 | hypothetical protein (*C. neoformans*) | 1.00E-48 |
| WRIS_2615 | GR303623 | 1 | 669 | hypothetical protein (*U. maydis*) | 2.00E-17 |
| WRIS_2663 | GR303640 | 1 | 610 | hypothetical protein (*U. maydis*) | 3.00E-38 |
| WRIS_267 | GR303650 | 1 | 356 | hypothetical protein (*U. maydis*) | 2.00E-16 |
| WRIS_2715 | GR303666 | 1 | 634 | hypothetical protein (*U. maydis*) | 1.00E-46 |
| WRIS_2741 | GR303678 | 1 | 759 | hypothetical protein (*P. nodorum*) | 3.00E-53 |
| WRIS_2756 | GR303689 | 1 | 784 | hypothetical protein (*Aspergillus nidulans*) | 2.00E-19 |
| WRIS_290 | GR303750 | 1 | 472 | hypothetical protein (*C. neoformans*) | 1.00E-17 |
| WRIS_2942 | GR303762 | 1 | 601 | hypothetical protein (*Aspergillus nidulans*) | 2.00E-54 |
| WRIS_2988 | GR303785 | 1 | 268 | hypothetical protein (*Yarrowia lipolytica*) | 6.00E-15 |
| WRIS_2992 | GR303789 | 1 | 729 | conserved hypothetical protein (*Aspergillus terreus*) | 8.00E-20 |
| WRIS_3042 | GR303811 | 1 | 736 | hypothetical protein (*U. maydis*) | 6.00E-61 |
| WRIS_3101 | GR303837 | 1 | 475 | hypothetical protein (*Debaryomyces hansenii*) | 5.00E-08 |
| WRIS_3109 | GR303842 | 1 | 501 | hypothetical protein (*U. maydis*) | 8.00E-30 |
| WRIS_3138 | GR303855 | 1 | 435 | hypothetical protein (*U. maydis*) | 5.00E-49 |
| WRIS_3176 | GR303869 | 1 | 723 | hypothetical protein (*M. grisea*) | 5.00E-68 |
| WRIS_3206 | GR303884 | 1 | 683 | hypothetical protein (*Aspergillus nidulans*) | 2.00E-23 |
| WRIS_3286 | GR303913 | 1 | 350 | hypothetical protein (*Neurospora crassa*) | 3.00E-11 |
| WRIS_3300 | GR303921 | 1 | 687 | hypothetical protein (*P. nodorum*) | 6.00E-16 |
| WRIS_3315 | GR303926 | 1 | 760 | hypothetical protein (*U. maydis*) | 5.00E-25 |
| WRIS_3359 | GR303943 | 1 | 713 | hypothetical protein (*Gibberella zeae*) | 1.00E-35 |
| WRIS_3385 | GR303951 | 1 | 707 | hypothetical protein (*C. neoformans*) | 2.00E-10 |
| WRIS_3433 | GR303974 | 1 | 326 | hypothetical protein (*C. neoformans*) | 3.00E-13 |
| WRIS_3441 | GR303978 | 1 | 760 | hypothetical protein (*Coccidioides immitis*) | 7.00E-14 |
| WRIS_3453 | GR303984 | 1 | 222 | hypothetical protein (*Yarrowia lipolytica*) | 1.00E-25 |
| WRIS_3460 | GR303986 | 1 | 688 | hypothetical protein (*S. pombe*) | 3.00E-33 |
| WRIS_3549 | GR304022 | 1 | 775 | hypothetical protein (*U. maydis*) | 1.00E-46 |
| WRIS_3560 | GR304027 | 1 | 422 | hypothetical protein (*Yarrowia lipolytica*) | 7.00E-16 |
| WRIS_3562 | GR304029 | 1 | 635 | hypothetical protein (*Coccidioides immitis*) | 5.00E-35 |
| WRIS_3592 | GR304036 | 1 | 704 | hypothetical protein (*U. maydis*) | 1.00E-35 |
| WRIS_359 | GR304038 | 1 | 683 | hypothetical protein (*U. maydis*) | 6.00E-38 |
| WRIS_3622 | GR304050 | 1 | 651 | hypothetical protein (*U. maydis*) | 7.00E-33 |
| WRIS_3660 | GR304067 | 1 | 406 | hypothetical protein (*U. maydis*) | 3.00E-12 |
| WRIS_3679 | GR304073 | 1 | 686 | hypothetical protein (*U. maydis*) | 3.00E-19 |
| WRIS_3689 | GR304076 | 1 | 605 | hypothetical protein (*C. neoformans*) | 2.00E-07 |
| WRIS_3718 | GR304088 | 1 | 663 | hypothetical protein (*Aspergillus nidulans*) | 1.00E-06 |
| WRIS_3719 | GR304089 | 1 | 654 | hypothetical protein (*U. maydis*) | 3.00E-44 |
| WRIS_3752 | GR304101 | 1 | 525 | hypothetical protein (*U. maydis*) | 1.00E-27 |
| WRIS_375 | GR304103 | 1 | 419 | hypothetical protein (*U. maydis*) | 7.00E-08 |
| WRIS_3826 | GR304118 | 1 | 641 | hypothetical protein (*U. maydis*) | 3.00E-79 |
| WRIS_3837 | GR304123 | 1 | 365 | conserved hypothetical protein (*Aspergillus terreus*) | 5.00E-11 |
| WRIS_3893 | GR304144 | 1 | 465 | hypothetical protein (*U. maydis*) | 4.00E-40 |
| WRIS_3934 | GR304153 | 1 | 463 | hypothetical protein (*Neurospora crassa*) | 1.00E-11 |
| WRIS_4032 | GR304174 | 1 | 659 | hypothetical protein (*C. neoformans*) | 3.00E-38 |
| WRIS_4100 | GR304198 | 1 | 476 | hypothetical protein (*U. maydis*) | 3.00E-17 |
| WRIS_4110 | GR304204 | 1 | 494 | hypothetical protein (*U. maydis*) | 5.00E-37 |
| WRIS_4143 | GR304217 | 1 | 628 | hypothetical protein (*U. maydis*) | 3.00E-52 |
| WRIS_4151 | GR304221 | 1 | 676 | hypothetical protein (*C. neoformans*) | 1.00E-15 |
| WRIS_4157 | GR304224 | 1 | 555 | hypothetical protein (*Neurospora crassa*) | 2.00E-35 |
| WRIS_4228 | GR304245 | 1 | 511 | hypothetical protein (*U. maydis*) | 8.00E-30 |
| WRIS_422 | GR304246 | 1 | 716 | hypothetical protein (*Gibberella zeae*) | 5.00E-06 |
| WRIS_4243 | GR304251 | 1 | 601 | hypothetical protein (*P. nodorum*) | 9.00E-20 |
| WRIS_4252 | GR304255 | 1 | 559 | hypothetical protein (*U. maydis*) | 5.00E-30 |
| WRIS_4276 | GR304263 | 1 | 619 | hypothetical protein (*Yarrowia lipolytica*) | 3.00E-11 |
| WRIS_4280 | GR304265 | 1 | 655 | hypothetical protein (*C. neoformans*) | 1.00E-10 |
| WRIS_4278 | GR304264 | 1 | 609 | hypothetical protein (*U. maydis*) | 1.00E-72 |
| WRIS_4320 | GR304282 | 1 | 719 | hypothetical protein (*U. maydis*) | 1.00E-48 |
| WRIS_4329 | GR304287 | 1 | 576 | hypothetical protein (*S. pombe*) | 1.00E-36 |
| WRIS_4346 | GR304292 | 1 | 519 | hypothetical protein (*C. neoformans*) | 9.00E-38 |
| WRIS_4374 | GR304302 | 1 | 709 | hypothetical protein (*U. maydis*) | 9.00E-45 |
| WRIS_4388 | GR304307 | 1 | 625 | hypothetical protein (*C. neoformans*) | 3.00E-06 |
| WRIS_4435 | GR304325 | 1 | 662 | hypothetical protein (*Coccidioides immitis*) | 9.00E-12 |
| WRIS_4448 | GR304330 | 1 | 669 | hypothetical protein (*C. neoformans*) | 2.00E-16 |
| WRIS_4453 | GR304332 | 1 | 594 | hypothetical protein (*Gibberella zeae*) | 3.00E-06 |
| WRIS_4470 | GR304338 | 1 | 687 | hypothetical protein (*C. neoformans*) | 1.00E-24 |
| WRIS_4474 | GR304339 | 1 | 550 | hypothetical protein (*Gibberella zeae*) | 6.00E-22 |
| WRIS_4479 | GR304341 | 1 | 627 | hypothetical protein (*U. maydis*) | 3.00E-48 |
| WRIS_4536 | GR304368 | 1 | 788 | hypothetical protein (*U. maydis*) | 6.00E-40 |
| WRIS_4565 | GR304384 | 1 | 443 | hypothetical protein (*U. maydis*) | 1.00E-52 |
| WRIS_4681 | GR304422 | 1 | 418 | hypothetical protein (*U. maydis*) | 7.00E-35 |
| WRIS_4716 | GR304435 | 1 | 527 | hypothetical protein (*U. maydis*) | 9.00E-51 |
| WRIS_4726 | GR304442 | 1 | 439 | hypothetical protein (*Dictyostelium discoideum*) | 2.00E-08 |
| WRIS_4729 | GR304444 | 1 | 638 | hypothetical protein (*C. neoformans*) | 3.00E-31 |
| WRIS_4733 | GR304448 | 1 | 506 | hypothetical protein (*U. maydis*) | 5.00E-30 |
| WRIS_4736 | GR304451 | 1 | 673 | hypothetical protein (*U. maydis*) | 2.00E-70 |
| WRIS_4811 | GR304484 | 1 | 635 | hypothetical protein (*U. maydis*) | 1.00E-46 |
| WRIS_4813 | GR304486 | 1 | 637 | hypothetical protein (*Coccidioides immitis*) | 1.00E-54 |
| WRIS_4819 | GR304488 | 1 | 670 | unnamed protein product (*Aspergillus oryzae*) | 2.00E-50 |
| WRIS_4899 | GR304533 | 1 | 658 | hypothetical protein (*U. maydis*) | 7.00E-88 |
| WRIS_4900 | GR304535 | 1 | 669 | hypothetical protein (*U. maydis*) | 5.00E-74 |
| WRIS_4921 | GR304548 | 1 | 682 | hypothetical protein (*C. neoformans*) | 1.00E-24 |
| WRIS_4925 | GR304551 | 1 | 661 | unnamed protein product (*Aspergillus oryzae*) | 2.00E-39 |
| WRIS_492 | GR304553 | 1 | 678 | hypothetical protein (*U. maydis*) | 8.00E-59 |
| WRIS_4945 | GR304557 | 1 | 412 | hypothetical protein (*U. maydis*) | 1.00E-27 |
| WRIS_4964 | GR304566 | 1 | 713 | hypothetical protein (*U. maydis*) | 2.00E-59 |
| WRIS_5032 | GR304598 | 1 | 666 | hypothetical protein (*U. maydis*) | 7.00E-33 |
| WRIS_508 | GR304619 | 1 | 737 | hypothetical protein (*U. maydis*) | 6.00E-07 |
| WRIS_4781 | GR304467 | 1 | 428 | hypothetical protein (*U. maydis*) | 7.00E-19 |
| WRIS_5100 | GR304624 | 1 | 486 | conserved hypothetical protein (*Aspergillus terreus*) | 1.00E-10 |
| WRIS_5116 | GR304634 | 1 | 727 | hypothetical protein (*U. maydis*) | 2.00E-54 |
| WRIS_5122 | GR304638 | 1 | 727 | hypothetical protein (*U. maydis*) | 4.00E-33 |
| WRIS_5149 | GR304644 | 1 | 712 | hypothetical protein (*C. neoformans*) | 1.00E-25 |
| WRIS_5170 | GR304657 | 1 | 731 | hypothetical protein (*Magnaporthe grisea*) | 4.00E-25 |
| WRIS_5183 | GR304663 | 1 | 703 | hypothetical protein (*Chaetomium globosum*) | 1.00E-06 |
| WRIS_5188 | GR304664 | 1 | 567 | hypothetical protein (*U. maydis*) | 3.00E-15 |
| WRIS_5207 | GR304672 | 1 | 732 | hypothetical protein (*Magnaporthe grisea*) | 1.00E-06 |
| WRIS_5225 | GR304678 | 1 | 732 | hypothetical protein (*U. maydis*) | 4.00E-65 |
| WRIS_5242 | GR304683 | 1 | 421 | unnamed protein product (*Aspergillus oryzae*) | 3.00E-16 |
| WRIS_5273 | GR304695 | 1 | 718 | hypothetical protein (*C. neoformans*) | 7.00E-32 |
| WRIS_5291 | GR304701 | 1 | 605 | hypothetical protein (*U. maydis*) | 2.00E-59 |
| WRIS_5333 | GR304715 | 1 | 556 | hypothetical protein (*U. maydis*) | 6.00E-17 |
| WRIS_5348 | GR304720 | 1 | 636 | hypothetical protein (*U. maydis*) | 8.00E-47 |
| WRIS_5356 | GR304724 | 1 | 758 | hypothetical protein (*U. maydis*) | 6.00E-74 |
| WRIS_5370 | GR304730 | 1 | 529 | hypothetical protein (*C. neoformans*) | 1.00E-31 |
| WRIS_5377 | GR304734 | 1 | 738 | hypothetical protein (*C. neoformans*) | 1.00E-60 |
| WRIS_5382 | GR304737 | 1 | 578 | hypothetical protein (*U. maydis*) | 2.00E-41 |
| WRIS_5392 | GR304740 | 1 | 767 | hypothetical protein (*Aspergillus nidulans*) | 5.00E-20 |
| WRIS_539 | GR304743 | 1 | 578 | hypothetical protein (*Yarrowia lipolytica*) | 1.00E-24 |
| WRIS_53 | GR304744 | 1 | 717 | unnamed protein product (*Aspergillus oryzae*) | 1.00E-12 |
| WRIS_5470 | GR304769 | 1 | 596 | hypothetical protein (*U. maydis*) | 9.00E-07 |
| WRIS_5495 | GR304780 | 1 | 505 | hypothetical protein (*Neurospora crassa*) | 2.00E-19 |
| WRIS_5500 | GR304782 | 1 | 332 | unnamed protein product (*Podospora anserine*) | 4.00E-28 |
| WRIS_5521 | GR304793 | 1 | 720 | hypothetical protein (*U. maydis*) | 1.00E-114 |
| WRIS_5544 | GR304800 | 1 | 559 | hypothetical protein (*U. maydis*) | 3.00E-17 |
| WRIS_5545 | GR304801 | 1 | 751 | hypothetical protein (*Gibberella zeae*) | 7.00E-57 |
| WRIS_5549 | GR304804 | 1 | 716 | hypothetical protein (*U. maydis*) | 8.00E-14 |
| WRIS_5577 | GR304814 | 1 | 764 | hypothetical protein (*Debaryomyces hansenii*) | 1.00E-46 |
| WRIS_5580 | GR304815 | 1 | 741 | hypothetical protein (S. pombe) | 6.00E-32 |
| WRIS_5581 | GR304816 | 1 | 727 | hypothetical protein (*Aspergillus nidulans*) | 1.00E-16 |
| WRIS_5601 | GR304824 | 1 | 758 | hypothetical protein (*U. maydis*) | 1.00E-70 |
| WRIS_5624 | GR304833 | 1 | 689 | hypothetical protein (*Gibberella zeae*) | 4.00E-13 |
| WRIS_562 | GR304835 | 1 | 696 | hypothetical protein (*C. neoformans*) | 6.00E-64 |
| WRIS_5632 | GR304837 | 1 | 680 | hypothetical protein (*U. maydis*) | 2.00E-13 |
| WRIS_5665 | GR304851 | 1 | 694 | hypothetical protein (*Yarrowia lipolytica*) | 5.00E-19 |
| WRIS_5772 | GR304886 | 1 | 710 | unnamed protein product (*Aspergillus oryzae*) | 2.00E-60 |
| WRIS_5788 | GR304896 | 1 | 631 | hypothetical protein (*U. maydis*) | 1.00E-41 |
| WRIS_5826 | GR304912 | 1 | 733 | hypothetical protein (*U. maydis*) | 4.00E-70 |
| WRIS_5845 | GR304921 | 1 | 682 | hypothetical protein (*C. neoformans*) | 8.00E-74 |
| WRIS_5875 | GR304933 | 1 | 562 | hypothetical protein (*Coccidioides immitis*) | 8.00E-06 |
| WRIS_5884 | GR304939 | 1 | 583 | hypothetical protein (*C. neoformans*) | 5.00E-13 |
| WRIS_5913 | GR304955 | 1 | 724 | hypothetical protein (*Yarrowia lipolytica*) | 1.00E-22 |
| WRIS_5935 | GR304961 | 1 | 651 | hypothetical protein (*C. neoformans*) | 7.00E-11 |
| WRIS_5948 | GR304965 | 1 | 753 | hypothetical protein (*C. neoformans*) | 4.00E-33 |
| WRIS_598 | GR304985 | 1 | 674 | hypothetical protein (*U. maydis*) | 1.00E-26 |
| WRIS_5990 | GR304986 | 1 | 707 | hypothetical protein (*C. neoformans*) | 2.00E-48 |
| WRIS_608 | GR304992 | 1 | 648 | hypothetical protein (*U. maydis*) | 3.00E-74 |
| WRIS_64 | GR305004 | 1 | 479 | hypothetical protein (*C. neoformans*) | 1.00E-31 |
| WRIS_71 | GR305030 | 1 | 596 | hypothetical protein (*Gibberella zeae*) | 3.00E-75 |
| WRIS_762 | GR305036 | 1 | 415 | Hypothetical protein (*Thermoanaero*) | 6.00E-23 |
| WRIS_787 | GR305045 | 1 | 603 | hypothetical protein (*S. pombe*) | 3.00E-06 |
| WRIS_824 | GR305060 | 1 | 606 | hypothetical protein (*U. maydis*) | 2.00E-15 |
| WRIS_836 | GR305066 | 1 | 590 | hypothetical protein (*U. maydis*) | 1.00E-61 |
| WRIS_849 | GR305070 | 1 | 715 | hypothetical protein (*Debaryomyces hansenii*) | 3.00E-31 |
| WRIS_864 | GR305079 | 1 | 531 | hypothetical protein (*U. maydis*) | 2.00E-35 |
| WRIS_865 | GR305080 | 1 | 516 | hypothetical protein (*U. maydis*) | 4.00E-36 |
| WRIS_883 | GR305084 | 1 | 670 | hypothetical protein (*Gibberella zeae*) | 2.00E-17 |
| WRIS_892 | GR305087 | 1 | 690 | hypothetical protein (*U. maydis*) | 9.00E-42 |
| WRIS_899 | GR305089 | 1 | 744 | hypothetical protein (*C. neoformans*) | 4.00E-16 |
| WRIS_959 | GR305110 | 1 | 326 | hypothetical protein (*U. maydis*) | 2.00E-22 |
| WRIS_971 | GR305114 | 1 | 663 | hypothetical protein (*Gibberella zeae*) | 5.00E-17 |
| WRIS_986 | GR305121 | 1 | 285 | hypothetical protein (*U. maydis*) | 3.00E-06 |
| WRIS_987 | GR305122 | 1 | 718 | hypothetical protein (*U. maydis*) | 5.00E-70 |
| WRIS_5787 | GR304895 | 1 | 504 | hypothetical protein (*C. neoformans*) | 4.00E-46 |
