# Supplementary material for: Identification of expressed genes during compatible interaction between stripe rust (Puccinia striiformis) and wheat using a cDNA library
Source: BMC Genomics. 2009 Dec 8;10:586. doi: 10.1186/1471-2164-10-586 (PMC3087560; doi:10.1186/1471-2164-10-586)
Supplement: Additional file 3 — Unisequences that do not have any hits in the GenBank databases. These data provides the ESTs that do not have hits in the GenBank databases. [file 1471-2164-10-586-S3.DOC]

**Additional file 3: Unisequences without significant hits to public databases.**

| **User ID** | **Accession No.** | **Copy no.** | **Length** |
| --- | --- | --- | --- |
| WRIC_2 | GR302386 | 2 | 667 |
| WRIC_5 | GR302389 | 2 | 684 |
| WRIC_8 | GR302392 | 3 | 926 |
| WRIC_15 | GR302399 | 2 | 798 |
| WRIC_19 | GR302403 | 3 | 465 |
| WRIC_25 | GR302409 | 2 | 204 |
| WRIC_31 | GR302415 | 2 | 536 |
| WRIC_33 | GR302417 | 2 | 974 |
| WRIC_47 | GR302431 | 18 | 696 |
| WRIC_50 | GR302434 | 2 | 376 |
| WRIC_60 | GR302444 | 2 | 348 |
| WRIC_74 | GR302458 | 4 | 592 |
| WRIC_88 | GR302472 | 2 | 827 |
| WRIC_90 | GR302474 | 2 | 126 |
| WRIC_93 | GR302477 | 3 | 1009 |
| WRIC_103 | GR302487 | 2 | 219 |
| WRIC_114 | GR302498 | 8 | 543 |
| WRIC_117 | GR302501 | 2 | 380 |
| WRIC_124 | GR302508 | 7 | 604 |
| WRIC_145 | GR302529 | 2 | 355 |
| WRIC_150 | GR302534 | 4 | 581 |
| WRIC_153 | GR302537 | 2 | 754 |
| WRIC_157 | GR302541 | 5 | 387 |
| WRIC_172 | GR302556 | 2 | 264 |
| WRIC_176 | GR302560 | 12 | 642 |
| WRIC_184 | GR302568 | 2 | 275 |
| WRIC_194 | GR302578 | 7 | 538 |
| WRIC_199 | GR302583 | 2 | 306 |
| WRIC_200 | GR302584 | 6 | 1048 |
| WRIC_203 | GR302587 | 2 | 567 |
| WRIC_205 | GR302589 | 4 | 531 |
| WRIC_207 | GR302591 | 3 | 672 |
| WRIC_211 | GR302595 | 8 | 1348 |
| WRIC_214 | GR302598 | 2 | 954 |
| WRIC_219 | GR302603 | 2 | 427 |
| WRIC_221 | GR302605 | 3 | 746 |
| WRIC_230 | GR302614 | 2 | 695 |
| WRIC_242 | GR302626 | 2 | 414 |
| WRIC_256 | GR302640 | 3 | 475 |
| WRIC_258 | GR302642 | 3 | 703 |
| WRIC_274 | GR302658 | 2 | 285 |
| WRIC_276 | GR302660 | 4 | 711 |
| WRIC_290 | GR302674 | 3 | 634 |
| WRIC_295 | GR302679 | 3 | 442 |
| WRIC_296 | GR302680 | 5 | 596 |
| WRIC_305 | GR302689 | 2 | 562 |
| WRIC_308 | GR302692 | 2 | 468 |
| WRIC_315 | GR302699 | 2 | 517 |
| WRIC_318 | GR302702 | 2 | 592 |
| WRIC_320 | GR302704 | 9 | 800 |
| WRIC_321 | GR302705 | 2 | 496 |
| WRIC_332 | GR302716 | 4 | 855 |
| WRIC_335 | GR302719 | 2 | 558 |
| WRIC_336 | GR302720 | 4 | 718 |
| WRIC_338 | GR302722 | 2 | 767 |
| WRIC_340 | GR302724 | 2 | 648 |
| WRIC_349 | GR302733 | 2 | 533 |
| WRIC_353 | GR302737 | 2 | 417 |
| WRIC_358 | GR302742 | 2 | 714 |
| WRIC_362 | GR302746 | 2 | 523 |
| WRIC_364 | GR302748 | 2 | 292 |
| WRIC_373 | GR302757 | 2 | 619 |
| WRIC_380 | GR302764 | 2 | 644 |
| WRIC_395 | GR302779 | 2 | 679 |
| WRIC_408 | GR302792 | 4 | 652 |
| WRIC_411 | GR302795 | 2 | 333 |
| WRIC_412 | GR302796 | 3 | 495 |
| WRIC_415 | GR302799 | 2 | 632 |
| WRIC_421 | GR302805 | 3 | 805 |
| WRIC_425 | GR302809 | 2 | 453 |
| WRIC_430 | GR302814 | 3 | 604 |
| WRIC_433 | GR302817 | 8 | 644 |
| WRIC_437 | GR302821 | 2 | 446 |
| WRIC_440 | GR302824 | 6 | 646 |
| WRIC_448 | GR302832 | 2 | 460 |
| WRIC_453 | GR302837 | 3 | 577 |
| WRIC_457 | GR302841 | 2 | 701 |
| WRIC_462 | GR302846 | 2 | 533 |
| WRIC_463 | GR302847 | 2 | 289 |
| WRIC_469 | GR302853 | 2 | 537 |
| WRIC_473 | GR302857 | 2 | 700 |
| WRIC_474 | GR302858 | 2 | 556 |
| WRIC_478 | GR302862 | 2 | 800 |
| WRIC_479 | GR302863 | 5 | 773 |
| WRIC_486 | GR302870 | 3 | 443 |
| WRIC_487 | GR302871 | 5 | 675 |
| WRIC_494 | GR302878 | 4 | 603 |
| WRIC_507 | GR302891 | 2 | 302 |
| WRIC_510 | GR302894 | 2 | 600 |
| WRIC_525 | GR302909 | 2 | 517 |
| WRIC_526 | GR302910 | 2 | 553 |
| WRIC_530 | GR302914 | 2 | 698 |
| WRIC_534 | GR302918 | 2 | 638 |
| WRIC_548 | GR302932 | 4 | 1330 |
| WRIC_557 | GR302941 | 32 | 630 |
| WRIC_561 | GR302945 | 2 | 416 |
| WRIC_562 | GR302946 | 2 | 623 |
| WRIC_564 | GR302948 | 2 | 474 |
| WRIC_581 | GR302965 | 3 | 317 |
| WRIS_1007 | GR302972 | 1 | 427 |
| WRIS_1022 | GR302977 | 1 | 717 |
| WRIS_1024 | GR302978 | 1 | 660 |
| WRIS_1029 | GR302981 | 1 | 430 |
| WRIS_1043 | GR302986 | 1 | 575 |
| WRIS_1057 | GR302989 | 1 | 209 |
| WRIS_105 | GR302991 | 1 | 693 |
| WRIS_1062 | GR302993 | 1 | 112 |
| WRIS_1066 | GR302995 | 1 | 263 |
| WRIS_1069 | GR302997 | 1 | 273 |
| WRIS_1070 | GR302999 | 1 | 296 |
| WRIS_1077 | GR303002 | 1 | 409 |
| WRIS_1078 | GR303003 | 1 | 632 |
| WRIS_1098 | GR303007 | 1 | 331 |
| WRIS_10 | GR303008 | 1 | 462 |
| WRIS_1103 | GR303010 | 1 | 221 |
| WRIS_1121 | GR303020 | 1 | 345 |
| WRIS_1137 | GR303026 | 1 | 150 |
| WRIS_1138 | GR303027 | 1 | 393 |
| WRIS_1142 | GR303028 | 1 | 352 |
| WRIS_1146 | GR303031 | 1 | 670 |
| WRIS_1147 | GR303032 | 1 | 563 |
| WRIS_1152 | GR303033 | 1 | 385 |
| WRIS_1161 | GR303036 | 1 | 631 |
| WRIS_1173 | GR303042 | 1 | 641 |
| WRIS_1175 | GR303043 | 1 | 663 |
| WRIS_1178 | GR303044 | 1 | 289 |
| WRIS_1185 | GR303046 | 1 | 611 |
| WRIS_1186 | GR303047 | 1 | 608 |
| WRIS_118 | GR303049 | 1 | 339 |
| WRIS_120 | GR303055 | 1 | 315 |
| WRIS_1219 | GR303058 | 1 | 304 |
| WRIS_1231 | GR303061 | 1 | 273 |
| WRIS_1236 | GR303064 | 1 | 675 |
| WRIS_1244 | GR303067 | 1 | 268 |
| WRIS_1251 | GR303070 | 1 | 388 |
| WRIS_1257 | GR303074 | 1 | 239 |
| WRIS_1263 | GR303075 | 1 | 468 |
| WRIS_1266 | GR303077 | 1 | 239 |
| WRIS_1270 | GR303079 | 1 | 185 |
| WRIS_1276 | GR303083 | 1 | 627 |
| WRIS_1285 | GR303087 | 1 | 578 |
| WRIS_1299 | GR303090 | 1 | 239 |
| WRIS_1306 | GR303093 | 1 | 231 |
| WRIS_1309 | GR303095 | 1 | 125 |
| WRIS_1315 | GR303098 | 1 | 697 |
| WRIS_1319 | GR303099 | 1 | 656 |
| WRIS_1322 | GR303100 | 1 | 340 |
| WRIS_1333 | GR303105 | 1 | 392 |
| WRIS_1336 | GR303107 | 1 | 503 |
| WRIS_1341 | GR303109 | 1 | 136 |
| WRIS_1372 | GR303119 | 1 | 518 |
| WRIS_1378 | GR303122 | 1 | 621 |
| WRIS_1384 | GR303127 | 1 | 333 |
| WRIS_1387 | GR303129 | 1 | 377 |
| WRIS_138 | GR303130 | 1 | 667 |
| WRIS_1406 | GR303134 | 1 | 188 |
| WRIS_1411 | GR303138 | 1 | 235 |
| WRIS_1413 | GR303139 | 1 | 316 |
| WRIS_1425 | GR303143 | 1 | 210 |
| WRIS_144 | GR303148 | 1 | 338 |
| WRIS_1459 | GR303153 | 1 | 263 |
| WRIS_1462 | GR303155 | 1 | 676 |
| WRIS_1465 | GR303157 | 1 | 365 |
| WRIS_1471 | GR303159 | 1 | 480 |
| WRIS_1485 | GR303164 | 1 | 528 |
| WRIS_1491 | GR303166 | 1 | 197 |
| WRIS_1503 | GR303170 | 1 | 436 |
| WRIS_1509 | GR303171 | 1 | 339 |
| WRIS_1511 | GR303173 | 1 | 211 |
| WRIS_1512 | GR303174 | 1 | 445 |
| WRIS_1518 | GR303177 | 1 | 490 |
| WRIS_1524 | GR303178 | 1 | 211 |
| WRIS_1528 | GR303180 | 1 | 307 |
| WRIS_1541 | GR303185 | 1 | 640 |
| WRIS_1543 | GR303186 | 1 | 455 |
| WRIS_1544 | GR303187 | 1 | 597 |
| WRIS_1545 | GR303188 | 1 | 266 |
| WRIS_1548 | GR303190 | 1 | 184 |
| WRIS_1550 | GR303191 | 1 | 333 |
| WRIS_1553 | GR303192 | 1 | 402 |
| WRIS_1573 | GR303198 | 1 | 662 |
| WRIS_1602 | GR303209 | 1 | 252 |
| WRIS_1609 | GR303212 | 1 | 411 |
| WRIS_1615 | GR303215 | 1 | 328 |
| WRIS_1617 | GR303216 | 1 | 242 |
| WRIS_1628 | GR303222 | 1 | 387 |
| WRIS_163 | GR303228 | 1 | 698 |
| WRIS_1648 | GR303231 | 1 | 217 |
| WRIS_1659 | GR303232 | 1 | 383 |
| WRIS_1664 | GR303233 | 1 | 463 |
| WRIS_1682 | GR303237 | 1 | 446 |
| WRIS_1686 | GR303238 | 1 | 305 |
| WRIS_1688 | GR303239 | 1 | 300 |
| WRIS_1692 | GR303241 | 1 | 239 |
| WRIS_1697 | GR303244 | 1 | 127 |
| WRIS_1698 | GR303245 | 1 | 265 |
| WRIS_1716 | GR303252 | 1 | 742 |
| WRIS_1747 | GR303264 | 1 | 323 |
| WRIS_1767 | GR303273 | 1 | 534 |
| WRIS_1772 | GR303274 | 1 | 501 |
| WRIS_1779 | GR303280 | 1 | 500 |
| WRIS_1780 | GR303281 | 1 | 559 |
| WRIS_178 | GR303287 | 1 | 333 |
| WRIS_1808 | GR303294 | 1 | 588 |
| WRIS_1810 | GR303296 | 1 | 354 |
| WRIS_1817 | GR303298 | 1 | 346 |
| WRIS_1824 | GR303303 | 1 | 225 |
| WRIS_1827 | GR303305 | 1 | 326 |
| WRIS_1831 | GR303307 | 1 | 674 |
| WRIS_1844 | GR303311 | 1 | 477 |
| WRIS_1849 | GR303313 | 1 | 501 |
| WRIS_1851 | GR303315 | 1 | 363 |
| WRIS_1865 | GR303322 | 1 | 681 |
| WRIS_1866 | GR303323 | 1 | 711 |
| WRIS_1873 | GR303325 | 1 | 396 |
| WRIS_1877 | GR303328 | 1 | 496 |
| WRIS_1882 | GR303329 | 1 | 396 |
| WRIS_1908 | GR303340 | 1 | 675 |
| WRIS_1911 | GR303343 | 1 | 377 |
| WRIS_1923 | GR303348 | 1 | 411 |
| WRIS_1926 | GR303349 | 1 | 141 |
| WRIS_192 | GR303352 | 1 | 382 |
| WRIS_1942 | GR303354 | 1 | 528 |
| WRIS_1945 | GR303355 | 1 | 333 |
| WRIS_1947 | GR303357 | 1 | 421 |
| WRIS_1958 | GR303359 | 1 | 290 |
| WRIS_197 | GR303366 | 1 | 391 |
| WRIS_2002 | GR303374 | 1 | 257 |
| WRIS_2007 | GR303376 | 1 | 402 |
| WRIS_2010 | GR303378 | 1 | 537 |
| WRIS_2017 | GR303379 | 1 | 242 |
| WRIS_2019 | GR303381 | 1 | 154 |
| WRIS_2041 | GR303389 | 1 | 564 |
| WRIS_2045 | GR303390 | 1 | 622 |
| WRIS_2067 | GR303400 | 1 | 651 |
| WRIS_2085 | GR303407 | 1 | 707 |
| WRIS_2100 | GR303413 | 1 | 315 |
| WRIS_2109 | GR303415 | 1 | 325 |
| WRIS_2117 | GR303419 | 1 | 579 |
| WRIS_2119 | GR303420 | 1 | 591 |
| WRIS_2123 | GR303422 | 1 | 623 |
| WRIS_2126 | GR303423 | 1 | 454 |
| WRIS_2128 | GR303424 | 1 | 511 |
| WRIS_2130 | GR303426 | 1 | 146 |
| WRIS_2132 | GR303427 | 1 | 670 |
| WRIS_2134 | GR303428 | 1 | 643 |
| WRIS_2140 | GR303431 | 1 | 197 |
| WRIS_2144 | GR303433 | 1 | 419 |
| WRIS_2160 | GR303436 | 1 | 739 |
| WRIS_2175 | GR303439 | 1 | 141 |
| WRIS_2179 | GR303442 | 1 | 572 |
| WRIS_2196 | GR303448 | 1 | 257 |
| WRIS_2202 | GR303449 | 1 | 388 |
| WRIS_2214 | GR303455 | 1 | 195 |
| WRIS_2227 | GR303462 | 1 | 305 |
| WRIS_2229 | GR303464 | 1 | 443 |
| WRIS_2242 | GR303468 | 1 | 623 |
| WRIS_224 | GR303471 | 1 | 608 |
| WRIS_2267 | GR303477 | 1 | 683 |
| WRIS_2272 | GR303478 | 1 | 657 |
| WRIS_2273 | GR303479 | 1 | 240 |
| WRIS_2290 | GR303484 | 1 | 307 |
| WRIS_2291 | GR303485 | 1 | 605 |
| WRIS_2293 | GR303487 | 1 | 374 |
| WRIS_2295 | GR303488 | 1 | 380 |
| WRIS_22 | GR303491 | 1 | 340 |
| WRIS_2301 | GR303492 | 1 | 497 |
| WRIS_2314 | GR303498 | 1 | 331 |
| WRIS_232 | GR303504 | 1 | 457 |
| WRIS_2330 | GR303505 | 1 | 287 |
| WRIS_2343 | GR303511 | 1 | 482 |
| WRIS_2351 | GR303516 | 1 | 209 |
| WRIS_2354 | GR303517 | 1 | 369 |
| WRIS_2358 | GR303518 | 1 | 356 |
| WRIS_2361 | GR303519 | 1 | 326 |
| WRIS_2362 | GR303520 | 1 | 645 |
| WRIS_2372 | GR303525 | 1 | 367 |
| WRIS_2374 | GR303526 | 1 | 558 |
| WRIS_237 | GR303528 | 1 | 245 |
| WRIS_2386 | GR303529 | 1 | 266 |
| WRIS_2404 | GR303538 | 1 | 251 |
| WRIS_240 | GR303541 | 1 | 777 |
| WRIS_2410 | GR303542 | 1 | 189 |
| WRIS_2412 | GR303544 | 1 | 202 |
| WRIS_2415 | GR303546 | 1 | 665 |
| WRIS_2429 | GR303550 | 1 | 557 |
| WRIS_2431 | GR303552 | 1 | 421 |
| WRIS_2440 | GR303554 | 1 | 237 |
| WRIS_2455 | GR303560 | 1 | 521 |
| WRIS_2457 | GR303562 | 1 | 436 |
| WRIS_2461 | GR303564 | 1 | 456 |
| WRIS_2468 | GR303569 | 1 | 578 |
| WRIS_2486 | GR303573 | 1 | 182 |
| WRIS_2488 | GR303574 | 1 | 381 |
| WRIS_248 | GR303576 | 1 | 746 |
| WRIS_2492 | GR303577 | 1 | 335 |
| WRIS_2497 | GR303580 | 1 | 165 |
| WRIS_2507 | GR303585 | 1 | 623 |
| WRIS_2516 | GR303589 | 1 | 264 |
| WRIS_251 | GR303591 | 1 | 496 |
| WRIS_2521 | GR303592 | 1 | 708 |
| WRIS_2527 | GR303595 | 1 | 396 |
| WRIS_2534 | GR303597 | 1 | 728 |
| WRIS_2555 | GR303604 | 1 | 719 |
| WRIS_2558 | GR303605 | 1 | 212 |
| WRIS_2563 | GR303607 | 1 | 372 |
| WRIS_2577 | GR303611 | 1 | 330 |
| WRIS_2586 | GR303613 | 1 | 627 |
| WRIS_2589 | GR303614 | 1 | 680 |
| WRIS_25 | GR303618 | 1 | 718 |
| WRIS_2604 | GR303620 | 1 | 539 |
| WRIS_2608 | GR303622 | 1 | 667 |
| WRIS_2634 | GR303629 | 1 | 115 |
| WRIS_2635 | GR303630 | 1 | 239 |
| WRIS_2645 | GR303632 | 1 | 618 |
| WRIS_264 | GR303634 | 1 | 443 |
| WRIS_2655 | GR303635 | 1 | 197 |
| WRIS_2662 | GR303639 | 1 | 754 |
| WRIS_2669 | GR303644 | 1 | 719 |
| WRIS_266 | GR303645 | 1 | 258 |
| WRIS_2672 | GR303647 | 1 | 690 |
| WRIS_2678 | GR303649 | 1 | 225 |
| WRIS_2682 | GR303651 | 1 | 429 |
| WRIS_2687 | GR303653 | 1 | 178 |
| WRIS_2690 | GR303656 | 1 | 726 |
| WRIS_2694 | GR303658 | 1 | 666 |
| WRIS_2703 | GR303662 | 1 | 200 |
| WRIS_2710 | GR303664 | 1 | 238 |
| WRIS_2713 | GR303665 | 1 | 366 |
| WRIS_272 | GR303673 | 1 | 302 |
| WRIS_2730 | GR303674 | 1 | 627 |
| WRIS_2740 | GR303677 | 1 | 148 |
| WRIS_2746 | GR303681 | 1 | 379 |
| WRIS_2748 | GR303683 | 1 | 741 |
| WRIS_2749 | GR303684 | 1 | 636 |
| WRIS_274 | GR303685 | 1 | 240 |
| WRIS_2768 | GR303691 | 1 | 221 |
| WRIS_2770 | GR303692 | 1 | 195 |
| WRIS_2786 | GR303699 | 1 | 700 |
| WRIS_2790 | GR303701 | 1 | 356 |
| WRIS_2794 | GR303702 | 1 | 419 |
| WRIS_27 | GR303705 | 1 | 631 |
| WRIS_2804 | GR303707 | 1 | 269 |
| WRIS_2843 | GR303718 | 1 | 296 |
| WRIS_2867 | GR303724 | 1 | 462 |
| WRIS_2868 | GR303725 | 1 | 489 |
| WRIS_286 | GR303726 | 1 | 367 |
| WRIS_2874 | GR303729 | 1 | 339 |
| WRIS_2875 | GR303730 | 1 | 269 |
| WRIS_2878 | GR303732 | 1 | 655 |
| WRIS_2879 | GR303733 | 1 | 413 |
| WRIS_2880 | GR303735 | 1 | 483 |
| WRIS_2888 | GR303737 | 1 | 410 |
| WRIS_2889 | GR303738 | 1 | 237 |
| WRIS_2896 | GR303742 | 1 | 354 |
| WRIS_2898 | GR303743 | 1 | 469 |
| WRIS_289 | GR303745 | 1 | 251 |
| WRIS_2902 | GR303746 | 1 | 740 |
| WRIS_2903 | GR303747 | 1 | 274 |
| WRIS_2916 | GR303752 | 1 | 328 |
| WRIS_2924 | GR303757 | 1 | 377 |
| WRIS_292 | GR303758 | 1 | 421 |
| WRIS_2934 | GR303760 | 1 | 169 |
| WRIS_2941 | GR303761 | 1 | 671 |
| WRIS_2951 | GR303766 | 1 | 245 |
| WRIS_2957 | GR303769 | 1 | 348 |
| WRIS_295 | GR303772 | 1 | 505 |
| WRIS_2961 | GR303773 | 1 | 167 |
| WRIS_2978 | GR303777 | 1 | 282 |
| WRIS_2979 | GR303778 | 1 | 540 |
| WRIS_2981 | GR303780 | 1 | 217 |
| WRIS_2983 | GR303782 | 1 | 363 |
| WRIS_2986 | GR303784 | 1 | 327 |
| WRIS_2991 | GR303788 | 1 | 273 |
| WRIS_3003 | GR303793 | 1 | 528 |
| WRIS_3007 | GR303795 | 1 | 420 |
| WRIS_3010 | GR303796 | 1 | 787 |
| WRIS_3016 | GR303798 | 1 | 319 |
| WRIS_3017 | GR303799 | 1 | 362 |
| WRIS_3025 | GR303803 | 1 | 626 |
| WRIS_3031 | GR303805 | 1 | 342 |
| WRIS_3032 | GR303806 | 1 | 619 |
| WRIS_3041 | GR303810 | 1 | 432 |
| WRIS_3051 | GR303815 | 1 | 357 |
| WRIS_3052 | GR303816 | 1 | 474 |
| WRIS_3056 | GR303818 | 1 | 168 |
| WRIS_3062 | GR303819 | 1 | 377 |
| WRIS_3065 | GR303821 | 1 | 142 |
| WRIS_3087 | GR303830 | 1 | 243 |
| WRIS_3089 | GR303832 | 1 | 228 |
| WRIS_3092 | GR303834 | 1 | 555 |
| WRIS_3108 | GR303841 | 1 | 273 |
| WRIS_310 | GR303843 | 1 | 353 |
| WRIS_3113 | GR303844 | 1 | 438 |
| WRIS_3118 | GR303846 | 1 | 653 |
| WRIS_3134 | GR303854 | 1 | 212 |
| WRIS_3139 | GR303856 | 1 | 562 |
| WRIS_3142 | GR303857 | 1 | 181 |
| WRIS_3143 | GR303858 | 1 | 580 |
| WRIS_3156 | GR303860 | 1 | 323 |
| WRIS_3165 | GR303863 | 1 | 490 |
| WRIS_3169 | GR303864 | 1 | 708 |
| WRIS_3190 | GR303877 | 1 | 567 |
| WRIS_319 | GR303882 | 1 | 661 |
| WRIS_3209 | GR303885 | 1 | 438 |
| WRIS_3220 | GR303886 | 1 | 757 |
| WRIS_3227 | GR303888 | 1 | 710 |
| WRIS_3231 | GR303890 | 1 | 266 |
| WRIS_3238 | GR303892 | 1 | 446 |
| WRIS_3266 | GR303902 | 1 | 308 |
| WRIS_3267 | GR303903 | 1 | 312 |
| WRIS_3278 | GR303907 | 1 | 463 |
| WRIS_3281 | GR303909 | 1 | 282 |
| WRIS_3287 | GR303914 | 1 | 378 |
| WRIS_3290 | GR303915 | 1 | 365 |
| WRIS_3295 | GR303918 | 1 | 302 |
| WRIS_329 | GR303919 | 1 | 392 |
| WRIS_32 | GR303920 | 1 | 289 |
| WRIS_3304 | GR303922 | 1 | 764 |
| WRIS_3305 | GR303923 | 1 | 333 |
| WRIS_3323 | GR303928 | 1 | 309 |
| WRIS_3336 | GR303933 | 1 | 194 |
| WRIS_3337 | GR303934 | 1 | 259 |
| WRIS_3344 | GR303938 | 1 | 400 |
| WRIS_3358 | GR303942 | 1 | 356 |
| WRIS_3360 | GR303944 | 1 | 416 |
| WRIS_3376 | GR303947 | 1 | 340 |
| WRIS_3383 | GR303950 | 1 | 427 |
| WRIS_3386 | GR303952 | 1 | 621 |
| WRIS_3387 | GR303953 | 1 | 259 |
| WRIS_338 | GR303955 | 1 | 331 |
| WRIS_3391 | GR303957 | 1 | 237 |
| WRIS_3404 | GR303959 | 1 | 666 |
| WRIS_3408 | GR303960 | 1 | 230 |
| WRIS_3416 | GR303966 | 1 | 688 |
| WRIS_3430 | GR303973 | 1 | 654 |
| WRIS_3434 | GR303975 | 1 | 549 |
| WRIS_3445 | GR303979 | 1 | 500 |
| WRIS_3447 | GR303980 | 1 | 715 |
| WRIS_3449 | GR303981 | 1 | 675 |
| WRIS_3464 | GR303988 | 1 | 707 |
| WRIS_3469 | GR303990 | 1 | 327 |
| WRIS_3471 | GR303991 | 1 | 533 |
| WRIS_3489 | GR303996 | 1 | 538 |
| WRIS_3490 | GR303997 | 1 | 651 |
| WRIS_3505 | GR304002 | 1 | 206 |
| WRIS_3512 | GR304004 | 1 | 768 |
| WRIS_3519 | GR304008 | 1 | 388 |
| WRIS_3521 | GR304009 | 1 | 335 |
| WRIS_3522 | GR304010 | 1 | 470 |
| WRIS_3525 | GR304013 | 1 | 417 |
| WRIS_3526 | GR304014 | 1 | 351 |
| WRIS_3533 | GR304017 | 1 | 231 |
| WRIS_3542 | GR304018 | 1 | 235 |
| WRIS_3547 | GR304021 | 1 | 448 |
| WRIS_3553 | GR304023 | 1 | 345 |
| WRIS_3554 | GR304024 | 1 | 479 |
| WRIS_3555 | GR304025 | 1 | 330 |
| WRIS_3561 | GR304028 | 1 | 264 |
| WRIS_3578 | GR304033 | 1 | 410 |
| WRIS_3585 | GR304034 | 1 | 292 |
| WRIS_3596 | GR304037 | 1 | 433 |
| WRIS_3600 | GR304039 | 1 | 719 |
| WRIS_3603 | GR304041 | 1 | 478 |
| WRIS_3604 | GR304042 | 1 | 235 |
| WRIS_3623 | GR304051 | 1 | 270 |
| WRIS_3629 | GR304054 | 1 | 322 |
| WRIS_3630 | GR304055 | 1 | 313 |
| WRIS_3653 | GR304065 | 1 | 249 |
| WRIS_365 | GR304066 | 1 | 173 |
| WRIS_366 | GR304071 | 1 | 679 |
| WRIS_3681 | GR304074 | 1 | 479 |
| WRIS_3699 | GR304080 | 1 | 595 |
| WRIS_3700 | GR304081 | 1 | 554 |
| WRIS_3701 | GR304082 | 1 | 422 |
| WRIS_3716 | GR304087 | 1 | 369 |
| WRIS_3735 | GR304094 | 1 | 734 |
| WRIS_3741 | GR304096 | 1 | 224 |
| WRIS_3743 | GR304097 | 1 | 254 |
| WRIS_374 | GR304099 | 1 | 350 |
| WRIS_3760 | GR304104 | 1 | 366 |
| WRIS_3779 | GR304106 | 1 | 611 |
| WRIS_3787 | GR304108 | 1 | 472 |
| WRIS_3791 | GR304109 | 1 | 454 |
| WRIS_3795 | GR304110 | 1 | 519 |
| WRIS_3802 | GR304112 | 1 | 226 |
| WRIS_3807 | GR304113 | 1 | 576 |
| WRIS_3835 | GR304122 | 1 | 624 |
| WRIS_383 | GR304124 | 1 | 476 |
| WRIS_3848 | GR304127 | 1 | 359 |
| WRIS_3857 | GR304129 | 1 | 434 |
| WRIS_3859 | GR304130 | 1 | 361 |
| WRIS_3864 | GR304133 | 1 | 229 |
| WRIS_3870 | GR304136 | 1 | 361 |
| WRIS_3872 | GR304137 | 1 | 421 |
| WRIS_3884 | GR304140 | 1 | 281 |
| WRIS_3886 | GR304141 | 1 | 500 |
| WRIS_3896 | GR304145 | 1 | 522 |
| WRIS_3900 | GR304146 | 1 | 665 |
| WRIS_391 | GR304147 | 1 | 373 |
| WRIS_3923 | GR304149 | 1 | 597 |
| WRIS_392 | GR304151 | 1 | 285 |
| WRIS_3945 | GR304155 | 1 | 505 |
| WRIS_3947 | GR304156 | 1 | 513 |
| WRIS_3948 | GR304157 | 1 | 403 |
| WRIS_3984 | GR304162 | 1 | 525 |
| WRIS_3996 | GR304165 | 1 | 674 |
| WRIS_4008 | GR304169 | 1 | 298 |
| WRIS_4014 | GR304172 | 1 | 279 |
| WRIS_4066 | GR304183 | 1 | 347 |
| WRIS_406 | GR304185 | 1 | 110 |
| WRIS_4071 | GR304186 | 1 | 518 |
| WRIS_4084 | GR304191 | 1 | 371 |
| WRIS_4093 | GR304195 | 1 | 410 |
| WRIS_4109 | GR304203 | 1 | 157 |
| WRIS_4112 | GR304205 | 1 | 470 |
| WRIS_4132 | GR304212 | 1 | 486 |
| WRIS_4138 | GR304215 | 1 | 657 |
| WRIS_4139 | GR304216 | 1 | 320 |
| WRIS_4150 | GR304220 | 1 | 376 |
| WRIS_4159 | GR304225 | 1 | 611 |
| WRIS_4165 | GR304228 | 1 | 378 |
| WRIS_4167 | GR304229 | 1 | 646 |
| WRIS_4176 | GR304232 | 1 | 454 |
| WRIS_4187 | GR304236 | 1 | 443 |
| WRIS_4193 | GR304238 | 1 | 616 |
| WRIS_4201 | GR304240 | 1 | 145 |
| WRIS_4203 | GR304241 | 1 | 334 |
| WRIS_4225 | GR304244 | 1 | 323 |
| WRIS_4231 | GR304248 | 1 | 398 |
| WRIS_4268 | GR304261 | 1 | 441 |
| WRIS_4283 | GR304266 | 1 | 547 |
| WRIS_4289 | GR304268 | 1 | 375 |
| WRIS_4305 | GR304273 | 1 | 246 |
| WRIS_4311 | GR304277 | 1 | 599 |
| WRIS_4321 | GR304283 | 1 | 329 |
| WRIS_4339 | GR304290 | 1 | 463 |
| WRIS_4341 | GR304291 | 1 | 407 |
| WRIS_4359 | GR304296 | 1 | 594 |
| WRIS_4369 | GR304300 | 1 | 333 |
| WRIS_4392 | GR304310 | 1 | 679 |
| WRIS_440 | GR304317 | 1 | 651 |
| WRIS_4413 | GR304319 | 1 | 286 |
| WRIS_4419 | GR304321 | 1 | 133 |
| WRIS_4432 | GR304324 | 1 | 423 |
| WRIS_4442 | GR304327 | 1 | 293 |
| WRIS_4444 | GR304329 | 1 | 620 |
| WRIS_4458 | GR304333 | 1 | 391 |
| WRIS_4462 | GR304334 | 1 | 214 |
| WRIS_446 | GR304337 | 1 | 260 |
| WRIS_4481 | GR304342 | 1 | 591 |
| WRIS_4490 | GR304348 | 1 | 617 |
| WRIS_4507 | GR304356 | 1 | 241 |
| WRIS_4518 | GR304358 | 1 | 275 |
| WRIS_4519 | GR304359 | 1 | 701 |
| WRIS_4520 | GR304360 | 1 | 569 |
| WRIS_4521 | GR304361 | 1 | 289 |
| WRIS_452 | GR304363 | 1 | 447 |
| WRIS_4532 | GR304365 | 1 | 432 |
| WRIS_4539 | GR304369 | 1 | 730 |
| WRIS_4540 | GR304370 | 1 | 639 |
| WRIS_4543 | GR304371 | 1 | 223 |
| WRIS_4547 | GR304373 | 1 | 283 |
| WRIS_4558 | GR304379 | 1 | 631 |
| WRIS_4562 | GR304381 | 1 | 193 |
| WRIS_4569 | GR304385 | 1 | 284 |
| WRIS_4571 | GR304386 | 1 | 726 |
| WRIS_4585 | GR304391 | 1 | 337 |
| WRIS_4593 | GR304394 | 1 | 314 |
| WRIS_4595 | GR304395 | 1 | 646 |
| WRIS_4598 | GR304396 | 1 | 405 |
| WRIS_4603 | GR304400 | 1 | 303 |
| WRIS_4611 | GR304402 | 1 | 539 |
| WRIS_4613 | GR304403 | 1 | 658 |
| WRIS_4614 | GR304404 | 1 | 309 |
| WRIS_4624 | GR304406 | 1 | 441 |
| WRIS_4630 | GR304408 | 1 | 518 |
| WRIS_4638 | GR304410 | 1 | 653 |
| WRIS_4641 | GR304413 | 1 | 193 |
| WRIS_4642 | GR304414 | 1 | 608 |
| WRIS_4677 | GR304419 | 1 | 362 |
| WRIS_4680 | GR304421 | 1 | 569 |
| WRIS_4684 | GR304424 | 1 | 278 |
| WRIS_468 | GR304425 | 1 | 682 |
| WRIS_4692 | GR304427 | 1 | 571 |
| WRIS_4700 | GR304429 | 1 | 455 |
| WRIS_4712 | GR304433 | 1 | 265 |
| WRIS_4717 | GR304436 | 1 | 486 |
| WRIS_471 | GR304437 | 1 | 140 |
| WRIS_4720 | GR304438 | 1 | 320 |
| WRIS_4725 | GR304441 | 1 | 499 |
| WRIS_4732 | GR304447 | 1 | 161 |
| WRIS_4734 | GR304449 | 1 | 226 |
| WRIS_4738 | GR304452 | 1 | 385 |
| WRIS_4747 | GR304454 | 1 | 520 |
| WRIS_4749 | GR304455 | 1 | 338 |
| WRIS_4751 | GR304456 | 1 | 729 |
| WRIS_4752 | GR304457 | 1 | 188 |
| WRIS_4757 | GR304459 | 1 | 381 |
| WRIS_4765 | GR304460 | 1 | 306 |
| WRIS_4777 | GR304464 | 1 | 479 |
| WRIS_4782 | GR304468 | 1 | 681 |
| WRIS_4783 | GR304469 | 1 | 294 |
| WRIS_4784 | GR304470 | 1 | 563 |
| WRIS_4795 | GR304475 | 1 | 315 |
| WRIS_479 | GR304478 | 1 | 682 |
| WRIS_47 | GR304479 | 1 | 325 |
| WRIS_4801 | GR304480 | 1 | 270 |
| WRIS_4803 | GR304481 | 1 | 378 |
| WRIS_4810 | GR304483 | 1 | 337 |
| WRIS_4821 | GR304490 | 1 | 165 |
| WRIS_4831 | GR304495 | 1 | 720 |
| WRIS_4846 | GR304501 | 1 | 291 |
| WRIS_4851 | GR304504 | 1 | 731 |
| WRIS_4852 | GR304505 | 1 | 708 |
| WRIS_4854 | GR304506 | 1 | 624 |
| WRIS_4855 | GR304507 | 1 | 325 |
| WRIS_4857 | GR304509 | 1 | 589 |
| WRIS_485 | GR304512 | 1 | 306 |
| WRIS_4863 | GR304514 | 1 | 695 |
| WRIS_4867 | GR304518 | 1 | 632 |
| WRIS_4868 | GR304519 | 1 | 632 |
| WRIS_4877 | GR304523 | 1 | 402 |
| WRIS_4894 | GR304530 | 1 | 356 |
| WRIS_4901 | GR304536 | 1 | 468 |
| WRIS_4906 | GR304538 | 1 | 399 |
| WRIS_4907 | GR304539 | 1 | 282 |
| WRIS_4911 | GR304542 | 1 | 540 |
| WRIS_4913 | GR304544 | 1 | 316 |
| WRIS_4917 | GR304546 | 1 | 479 |
| WRIS_4923 | GR304549 | 1 | 372 |
| WRIS_4937 | GR304556 | 1 | 744 |
| WRIS_4950 | GR304559 | 1 | 561 |
| WRIS_4958 | GR304562 | 1 | 500 |
| WRIS_495 | GR304563 | 1 | 394 |
| WRIS_4963 | GR304565 | 1 | 377 |
| WRIS_4965 | GR304567 | 1 | 652 |
| WRIS_4972 | GR304571 | 1 | 639 |
| WRIS_4991 | GR304577 | 1 | 261 |
| WRIS_5004 | GR304583 | 1 | 305 |
| WRIS_5010 | GR304586 | 1 | 427 |
| WRIS_5018 | GR304589 | 1 | 456 |
| WRIS_501 | GR304590 | 1 | 715 |
| WRIS_5027 | GR304593 | 1 | 718 |
| WRIS_5028 | GR304594 | 1 | 472 |
| WRIS_502 | GR304596 | 1 | 625 |
| WRIS_5033 | GR304599 | 1 | 734 |
| WRIS_5041 | GR304602 | 1 | 317 |
| WRIS_5042 | GR304603 | 1 | 443 |
| WRIS_5045 | GR304604 | 1 | 196 |
| WRIS_5048 | GR304606 | 1 | 694 |
| WRIS_5053 | GR304607 | 1 | 301 |
| WRIS_5059 | GR304610 | 1 | 507 |
| WRIS_5072 | GR304613 | 1 | 234 |
| WRIS_5075 | GR304614 | 1 | 455 |
| WRIS_5081 | GR304616 | 1 | 493 |
| WRIS_5095 | GR304623 | 1 | 369 |
| WRIS_5101 | GR304625 | 1 | 254 |
| WRIS_5106 | GR304628 | 1 | 412 |
| WRIS_5107 | GR304629 | 1 | 494 |
| WRIS_5111 | GR304631 | 1 | 205 |
| WRIS_5118 | GR304635 | 1 | 182 |
| WRIS_5119 | GR304636 | 1 | 462 |
| WRIS_5127 | GR304639 | 1 | 239 |
| WRIS_5136 | GR304641 | 1 | 417 |
| WRIS_5150 | GR304645 | 1 | 228 |
| WRIS_5155 | GR304646 | 1 | 729 |
| WRIS_5157 | GR304648 | 1 | 179 |
| WRIS_5159 | GR304650 | 1 | 685 |
| WRIS_5163 | GR304652 | 1 | 216 |
| WRIS_5165 | GR304654 | 1 | 591 |
| WRIS_5168 | GR304655 | 1 | 352 |
| WRIS_516 | GR304656 | 1 | 238 |
| WRIS_5172 | GR304658 | 1 | 163 |
| WRIS_5182 | GR304662 | 1 | 728 |
| WRIS_5197 | GR304668 | 1 | 247 |
| WRIS_51 | GR304671 | 1 | 322 |
| WRIS_5235 | GR304680 | 1 | 288 |
| WRIS_5243 | GR304684 | 1 | 336 |
| WRIS_5245 | GR304685 | 1 | 692 |
| WRIS_5251 | GR304688 | 1 | 794 |
| WRIS_5252 | GR304689 | 1 | 551 |
| WRIS_5253 | GR304690 | 1 | 443 |
| WRIS_5258 | GR304691 | 1 | 187 |
| WRIS_5264 | GR304693 | 1 | 497 |
| WRIS_527 | GR304696 | 1 | 337 |
| WRIS_5284 | GR304698 | 1 | 566 |
| WRIS_5293 | GR304703 | 1 | 627 |
| WRIS_5300 | GR304705 | 1 | 346 |
| WRIS_5302 | GR304706 | 1 | 602 |
| WRIS_5307 | GR304708 | 1 | 468 |
| WRIS_5326 | GR304713 | 1 | 352 |
| WRIS_5338 | GR304716 | 1 | 770 |
| WRIS_5341 | GR304718 | 1 | 549 |
| WRIS_5355 | GR304723 | 1 | 318 |
| WRIS_5366 | GR304727 | 1 | 694 |
| WRIS_5389 | GR304738 | 1 | 413 |
| WRIS_5410 | GR304748 | 1 | 379 |
| WRIS_5417 | GR304751 | 1 | 370 |
| WRIS_5430 | GR304755 | 1 | 721 |
| WRIS_5436 | GR304758 | 1 | 345 |
| WRIS_5440 | GR304760 | 1 | 787 |
| WRIS_5452 | GR304763 | 1 | 469 |
| WRIS_5467 | GR304768 | 1 | 325 |
| WRIS_5472 | GR304770 | 1 | 399 |
| WRIS_5475 | GR304771 | 1 | 619 |
| WRIS_5478 | GR304772 | 1 | 414 |
| WRIS_5489 | GR304778 | 1 | 702 |
| WRIS_5503 | GR304783 | 1 | 596 |
| WRIS_5504 | GR304784 | 1 | 304 |
| WRIS_5508 | GR304787 | 1 | 641 |
| WRIS_5518 | GR304790 | 1 | 421 |
| WRIS_5525 | GR304794 | 1 | 254 |
| WRIS_5526 | GR304795 | 1 | 229 |
| WRIS_5528 | GR304796 | 1 | 371 |
| WRIS_5536 | GR304799 | 1 | 696 |
| WRIS_5559 | GR304807 | 1 | 665 |
| WRIS_5571 | GR304811 | 1 | 571 |
| WRIS_5576 | GR304813 | 1 | 285 |
| WRIS_5588 | GR304817 | 1 | 436 |
| WRIS_5591 | GR304819 | 1 | 281 |
| WRIS_5592 | GR304820 | 1 | 616 |
| WRIS_5609 | GR304825 | 1 | 377 |
| WRIS_5614 | GR304827 | 1 | 345 |
| WRIS_5619 | GR304831 | 1 | 301 |
| WRIS_5629 | GR304834 | 1 | 567 |
| WRIS_5640 | GR304838 | 1 | 693 |
| WRIS_5641 | GR304839 | 1 | 373 |
| WRIS_5642 | GR304840 | 1 | 671 |
| WRIS_5654 | GR304847 | 1 | 225 |
| WRIS_5680 | GR304856 | 1 | 597 |
| WRIS_5682 | GR304857 | 1 | 216 |
| WRIS_569 | GR304861 | 1 | 195 |
| WRIS_570 | GR304863 | 1 | 346 |
| WRIS_5719 | GR304865 | 1 | 358 |
| WRIS_5733 | GR304870 | 1 | 494 |
| WRIS_5747 | GR304876 | 1 | 669 |
| WRIS_5755 | GR304879 | 1 | 294 |
| WRIS_5759 | GR304882 | 1 | 318 |
| WRIS_5761 | GR304883 | 1 | 352 |
| WRIS_5776 | GR304889 | 1 | 647 |
| WRIS_5777 | GR304890 | 1 | 375 |
| WRIS_5781 | GR304892 | 1 | 395 |
| WRIS_5783 | GR304894 | 1 | 525 |
| WRIS_5794 | GR304899 | 1 | 661 |
| WRIS_5799 | GR304902 | 1 | 311 |
| WRIS_5801 | GR304905 | 1 | 261 |
| WRIS_5802 | GR304906 | 1 | 371 |
| WRIS_5808 | GR304907 | 1 | 671 |
| WRIS_5815 | GR304909 | 1 | 736 |
| WRIS_5833 | GR304913 | 1 | 770 |
| WRIS_5834 | GR304914 | 1 | 462 |
| WRIS_5837 | GR304916 | 1 | 519 |
| WRIS_5842 | GR304919 | 1 | 541 |
| WRIS_5844 | GR304920 | 1 | 264 |
| WRIS_5855 | GR304925 | 1 | 708 |
| WRIS_5859 | GR304927 | 1 | 495 |
| WRIS_587 | GR304936 | 1 | 369 |
| WRIS_5893 | GR304943 | 1 | 287 |
| WRIS_5903 | GR304952 | 1 | 291 |
| WRIS_5910 | GR304954 | 1 | 457 |
| WRIS_5914 | GR304956 | 1 | 546 |
| WRIS_5923 | GR304958 | 1 | 303 |
| WRIS_5934 | GR304960 | 1 | 470 |
| WRIS_5961 | GR304971 | 1 | 457 |
| WRIS_5974 | GR304977 | 1 | 257 |
| WRIS_5988 | GR304983 | 1 | 508 |
| WRIS_5992 | GR304987 | 1 | 787 |
| WRIS_604 | GR304990 | 1 | 191 |
| WRIS_609 | GR304993 | 1 | 621 |
| WRIS_61 | GR304998 | 1 | 588 |
| WRIS_62 | GR305001 | 1 | 282 |
| WRIS_653 | GR305006 | 1 | 410 |
| WRIS_654 | GR305007 | 1 | 709 |
| WRIS_657 | GR305008 | 1 | 485 |
| WRIS_661 | GR305010 | 1 | 448 |
| WRIS_673 | GR305012 | 1 | 456 |
| WRIS_675 | GR305014 | 1 | 379 |
| WRIS_67 | GR305015 | 1 | 627 |
| WRIS_682 | GR305017 | 1 | 388 |
| WRIS_686 | GR305018 | 1 | 600 |
| WRIS_712 | GR305026 | 1 | 527 |
| WRIS_727 | GR305031 | 1 | 359 |
| WRIS_764 | GR305037 | 1 | 178 |
| WRIS_774 | GR305041 | 1 | 378 |
| WRIS_779 | GR305043 | 1 | 112 |
| WRIS_786 | GR305044 | 1 | 222 |
| WRIS_78 | GR305046 | 1 | 216 |
| WRIS_796 | GR305049 | 1 | 648 |
| WRIS_803 | GR305052 | 1 | 139 |
| WRIS_806 | GR305053 | 1 | 342 |
| WRIS_81 | GR305058 | 1 | 263 |
| WRIS_825 | GR305061 | 1 | 311 |
| WRIS_83 | GR305067 | 1 | 342 |
| WRIS_840 | GR305068 | 1 | 527 |
| WRIS_84 | GR305071 | 1 | 282 |
| WRIS_859 | GR305076 | 1 | 235 |
| WRIS_88 | GR305085 | 1 | 618 |
| WRIS_909 | GR305091 | 1 | 453 |
| WRIS_90 | GR305092 | 1 | 219 |
| WRIS_911 | GR305093 | 1 | 559 |
| WRIS_913 | GR305094 | 1 | 293 |
| WRIS_931 | GR305101 | 1 | 107 |
| WRIS_941 | GR305103 | 1 | 748 |
| WRIS_944 | GR305104 | 1 | 214 |
| WRIS_948 | GR305105 | 1 | 699 |
| WRIS_95 | GR305111 | 1 | 505 |
| WRIS_974 | GR305115 | 1 | 658 |
| WRIS_979 | GR305117 | 1 | 212 |
| WRIS_97 | GR305118 | 1 | 264 |
| WRIS_990 | GR305123 | 1 | 235 |
| WRIS_993 | GR305125 | 1 | 372 |
